# Mendelian randomization analysis to elucidate the causal relationship between small molecule metabolites and ovarian cancer risk

**DOI:** 10.3389/fonc.2023.1291033

**Published:** 2023-11-28

**Authors:** Xin Chang, Shijia Liu, Lu Han

**Affiliations:** ^1^ Department of Gynecology, Dalian Women and Children’s Medical Group, Dalian, Liaoning, China; ^2^ Department of Graduate, Dalian Medical University, Dalian, Liaoning, China

**Keywords:** ovarian cancer, Mendelian randomization, single nucleotide polymorphisms, amino acids, biomarkers

## Abstract

**Background:**

Small molecule metabolites are potential biomarkers for ovarian cancer. However, the causal relationship between small molecule metabolites and ovarian cancer remains unclear.

**Methods:**

Single nucleotide polymorphisms (SNPs) correlated with 53 distinct small molecule metabolites were identified as instrumental variables (IVs) from comprehensive genome-wide association studies. Aggregate data encompassing 25,509 cases of ovarian cancer and 40,941 controls of European descent were procured from the Ovarian Cancer Association Consortium. To evaluate causative associations, four Mendelian randomization techniques—including inverse-variance weighted, weighted median, maximum likelihood, and MR-Egger regression—were employed.

**Results:**

In total, 242 SNPs were delineated as IVs for the small molecule metabolites under consideration. A significant association with the overarching risk of ovarian cancer was observed for six distinct metabolites. Hexadecenoylcarnitine and methioninesulfoxide were associated with a 32% and 31% reduced risk, respectively. Fifteen metabolites were linked to subtype ovarian cancers. For instance, both methionine sulfoxide and tetradecanoyl carnitine exhibited an inverse association with the risk of clear cell and high-grade serous ovarian cancers. Conversely, tryptophan demonstrated a 1.72-fold elevated risk for endometrioid ovarian cancer.

**Conclusion:**

This study identified several metabolites with putative causal effects on ovarian cancer risk using Mendelian randomization analysis. The findings provide insight into the etiological role of small molecule metabolites and highlight potential early detection biomarkers for ovarian cancer. Subsequent investigations are imperative to corroborate these findings and elucidate the underlying pathophysiological mechanisms.

## 1 Introduction

Cancer remains one of the most formidable adversaries in the realm of global health, contributing significantly to the burden of disease and mortality, with its impact felt acutely in developing countries. Within this broader context, ovarian cancer (OC) emerges as a predominant gynecological malignancy. Recent statistics from 2020 have underscored this reality, revealing an estimated 310,000 new cases of OC and a deeply concerning figure of 210,000 deaths associated with the condition. The trajectory of OC is particularly alarming, with projections suggesting that by the year 2040, we may witness the global incidence of this cancer soar to approximately 434,184 cases (1–4). The high mortality rate is largely attributed to the asymptomatic nature and late diagnosis of OC (5). In diseases with a significant burden, such as OC, the underlying etiology and pathogenesis remain largely elusive. Established risk factors for OC encompass age at menarche, age at natural menopause, and age at diagnosis of endometriosis (6). Elevated dietary consumption of fiber and soy has demonstrated potential prophylactic benefits against OC (7, 8). Furthermore, a deficiency in vitamin D levels has been associated with an augmented risk of OC (9). Identification of novel biomarkers that can detect OC at an early stage or predict susceptibility is urgently needed to reduce disease burden.

Emerging evidence suggests that metabolic reprogramming is implicated in ovarian tumorigenesis and progression (10). Metabolomics profiling has revealed aberrant levels of various small molecule metabolites, such as amino acids, biogenic amines, acylcarnitines, and carbohydrates, in OC (10–13). These small molecules are involved in multiple oncogenic signaling pathways and may serve as diagnostic biomarkers or therapeutic targets. Recent comprehensive genome-wide association studies (GWAS) have delineated single nucleotide polymorphisms (SNPs) correlated with metabolic phenotypes. These SNPs can be judiciously employed as instrumental variables to infer putative causal associations between specific metabolites and disease outcomes (14–16). Conversely, a limited number of studies have delved into the relationship between OC and the small molecular derivatives of metabolism.

Mendelian randomization (MR) analysis employs genetic variants as instrumental variables (IVs), enhancing the robustness of causal inference and mitigating biases stemming from reverse causation and confounding (17). This methodology has been extensively employed to assess the putative causal role of alterable exposures in carcinogenesis (18). However, to date, no investigation has probed the potential causal implications of small molecular metabolites on OC via Mendelian randomization. In this study, we performed a two-sample MR analysis to evaluate putative causal associations of genetically predicted small molecule metabolites with OC risk. Findings from this study could uncover novel etiological mechanisms and guide future development of metabotype-based biomarkers for OC.

## 2 Materials and methods

### 2.1 Study design

**Figure 1:**
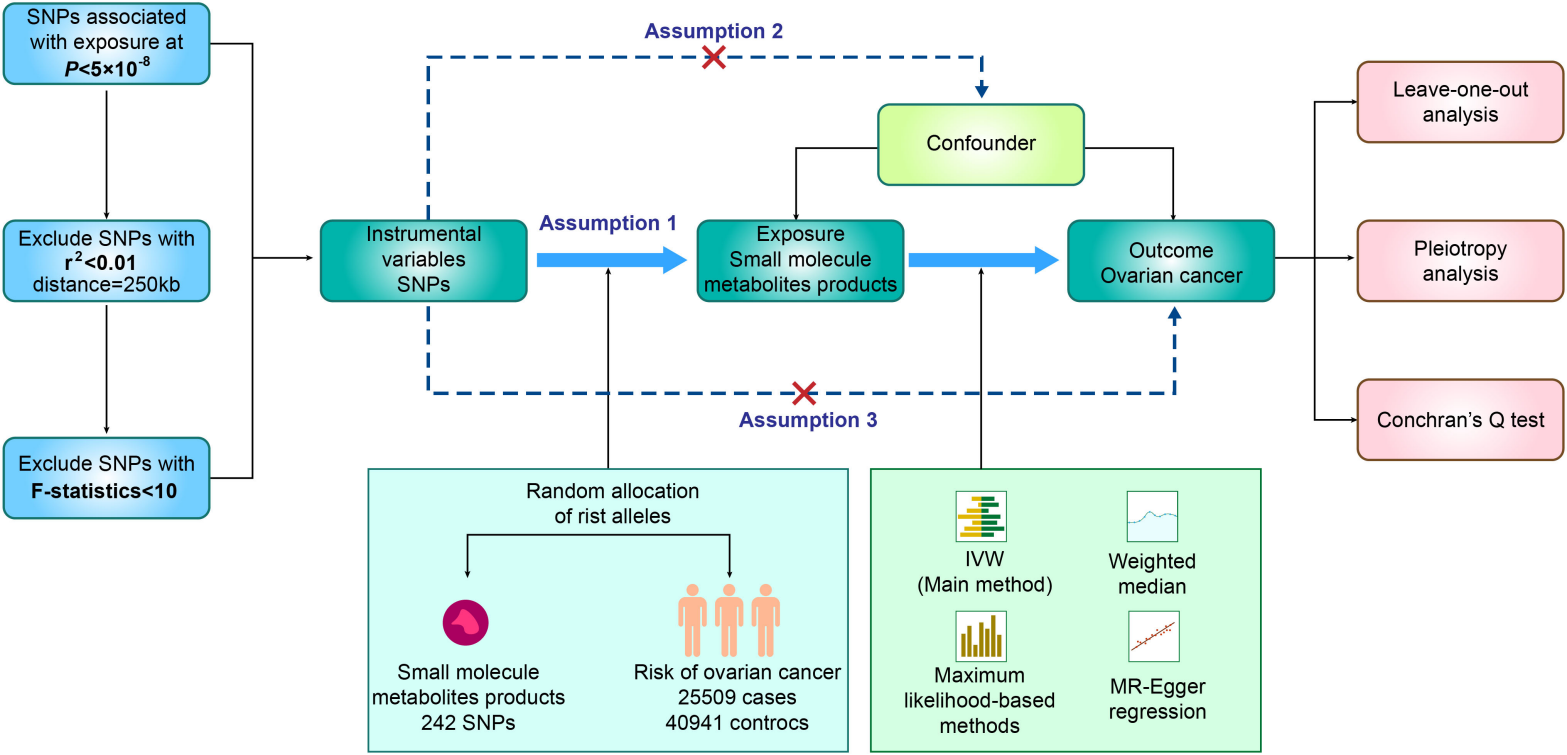
Flow chart of MR analysis and three assumptions in this study. GWAS, Genome-wide association study; MR, Mendelian randomization; SNP, single nucleotide polymorphism.

In our study, we utilized SNPs identified through GWAS as genetic instrumental variables (IVs), aiming to investigate the plausible causal connection between small molecule metabolic products and ovarian cancer. As presented in **Figure 1**, our two-sample MR study was built upon three principal assumptions (19). 1) Relevance assumption: The IVs had a strong connection to the exposure (19). 2) Independence assumption: There was no correlation between the IVs and any variables that affected both exposure and outcome (19). 3) Exclusion Restriction Assumption: The IVs exclusively influenced the exposure, without introducing any additional causal pathways that could impact the outcome (19). All the summary data utilized in our study were openly accessible to the general public (IEU OpenGWAS project). Additional data can be found in the **Supplementary Material** (**Supplementary Table 1**). As our research relied on publicly available GWAS data, no additional ethical approval was necessary.

### 2.2 Data source and study samples of ovarian cancer

This study considered a total of six frequently observed clinical phenotypes of ovarian cancer, specifically: OC, high-grade serous ovarian cancer (HGSOC), low malignant potential ovarian cancer (LMPOC), invasive mucinous ovarian cancer (IMOC), clear cell ovarian cancer (CCOC), and endometrioid ovarian cancer (EndoOC). The findings presented in this study are based on a genome-wide association studies conducted within the Ovarian Cancer Association Consortium (OCAC) (20). This thorough analysis was conducted using a dataset that encompassed 25,509 cases of ovarian cancer and 40,941 controls of European ancestry, enabling an exploration of the associations between genetic factors and ovarian cancer (21). The dataset encompasses 63 distinct genotyping project/case-control sets (21). Genomic information was acquired through direct genotyping utilizing an Illumina Custom Infinium array, known as OncoArray, featuring around 530,000 SNPs (21). To enhance the dataset’s comprehensiveness, imputation was executed utilizing the 1000 Genomes Project Phase 3 dataset as a reference (21). The cases encompassed the subsequent invasive epithelial ovarian cancer types: HGSOC (n = 13,037), LMPOC (n = 3,103), MOC (n = 1,417), CCOC (n = 1,366) and EndoOC (n = 2,810) (21). The majority of individuals were recruited from cancer genetics clinics, which also included some related individuals (21). More specific information regarding the cohorts, genotyping, quality control, and imputation can be viewed in previous studies (21).

### 2.3 Genetic instruments selection

The genetic components involving small molecule metabolic products, which encompass Acylcarnitines, Amino acids, Biogenic amines, and Hexose, were derived from a GWAS. This study involved a collective cohort of 86,507 adults with European heritage drawn from the Fenland cohort, with synergies established between the EPIC-Norfolk and INTERVAL studies (14). We first selected IVs for each small molecule products based on a strict cutoff of *P <*5×10^-8^. Independent SNPs (r2 < 0.01, distance = 250 kb) were preserved after calculating the linkage disequilibrium (LD) of related SNPs. Furthermore, the robustness of the genetic instruments was evaluated through F-statistics to mitigate potential biases from weak instruments. The F-statistics were computed using the formula: F-statistics = (Beta/Se) ^2^, with the mean serving as the comprehensive measure and an F-statistic > 10 signified substantial statistical potency (22, 23). Finally, a total of 242 SNPs associated with 53 small molecule products of metabolism were remained as the instrument variables (IVs). Detailed information of the IVs form small molecule products of metabolism were summarized in **Supplementary Table 2**, respectively.

### 2.4 Statistical analyses

Four methods including the inverse-variance-weighting (IVW), weighted median, maximum likelihood-based methods, and MR-Egger regression. maximum likelihood-based methods were performed to assess the causal association between small molecule products of metabolism and OC. The IVW method operates under the assumption of the validity of all instrumental variables, amalgamating their effects to produce an overall weighted outcome (24). Given the potential heterogeneity, the random effect and fixed effect IVW were both calculated and regarded as the main analyses (24). The weighted median estimator can generate resilient causal estimates, maintaining robustness even when up to 50% of instrumental variables may be invalid (25). Besides, under an assumption of a linear relationship between exposure and outcome, the maximum likelihood-based method offered normal bivariate distribution for the estimated causal association (26). Finally, the MR Egger method introduces an intercept term in the regression model to assess the directional pleiotropy (27). A substantially non-zero intercept term in statistical analysis signals the existence of pleiotropy and a breach in the fundamental Mendelian randomization assumption (27).

We employed the Cochran’s Q test to evaluate the heterogeneity among IVs (28). In case of notable heterogeneity being detected *(P* < 0.05), the random-effects model was employed; conversely, if heterogeneity was not significant (*P* > 0.05), the fixed-effects model was utilized (28). A leave-one-out analysis was conducted to pinpoint influential SNPs in the causal estimations. A threshold of statistical significance was set at *P* < 0.05 (two-sided). When the quantity of SNPs is fewer than four, the analysis is confined to using the IVW method. All analyses were performed using “TwoSampleMR”, and “gg-plot2” packages in R software (version 3.6.3, R Foundation for Statistical Computing, Vienna, Austria).

## 3 Results

### 3.1 Causal estimates of genetically predicted small molecule metabolic products on overall ovarian cancer

**Figure 2:**
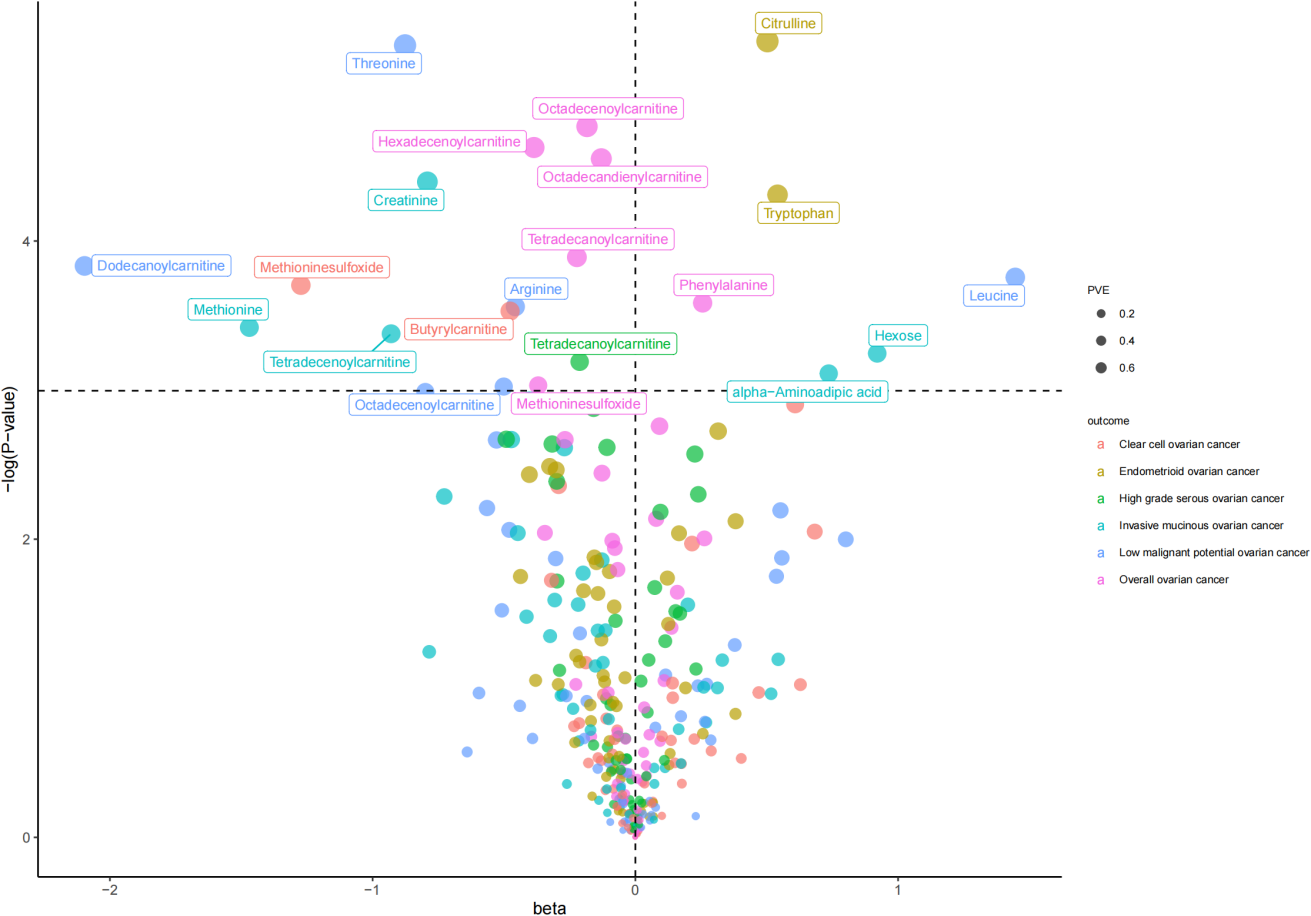
The volcano plot for inverse-variance-weighted method in the association between molecule metabolic products and ovarian cancer.

As shown in **Table 1** and **Figure 2**, we totally found six small molecule metabolic products were associated with overall OC. In brief, genetically predicted hexadecenoylcarnitine as well as methioninesulfoxide dropped a 32% (OR, 0.68; 95% CI =0.51-0.91, *P* = 0.010) and 31% (OR=0.69, 95% CI =0.48-1.00, *P* = 0.048) risk of overall OC by the Wald ratio method, respectively. This decreased risk was also observed in the association between octadecandienylcarnitine and overall OC, replicated by the Maximum likelihood method (OR = 0.88, 95% CI = 0.79-0.97, *P* =0.011 and weighted median approach (OR=0.86, 95% CI=0.77-0.96, *P*=0.007). Estimates in size were similar for the association of octadecenoylcarnitine and tetradecanoylcarnitine with overall OC (**Supplementary Table 3**). The heterogeneity test and pleiotropy test indicated that there no influence for the casual effect of octadecandienyl carnitine on overall ovarian cancer (*P*>0.05).

**Table 1:** The main result of small molecule metabolites and ovarian cancer risk.

Outcome	Exposure	Method	Number of SNP	OR	LCI	UCI	P-value	P for heterogeneity	P for pleiotropy
Overall ovarian cancer	Hexadecenoylcarnitine	Wald ratio	1	0.68	0.51	0.91	0.010	/	/
		Wald ratio	1	0.69	0.48	1.00	0.048	/	/
Overall ovarian cancer	Octadecandienylcarnitine	Inverse variance weighted (fixed effects)	4	0.88	0.80	0.97	0.011	0.426	
		Maximum likelihood	4	0.88	0.79	0.97	0.011		
		Simple median	4	0.88	0.76	1.01	0.075		
		Weighted median	4	0.86	0.77	0.96	0.007		
		MR Egger	4	0.84	0.64	1.11	0.341		0.766
Overall ovarian cancer	Octadecenoylcarnitine	Inverse variance weighted (fixed effects)	2	0.83	0.73	0.95	0.008	0.478	/
		Maximum likelihood	2	0.83	0.72	0.96	0.010		
Overall ovarian cancer	Phenylalanine	Inverse variance weighted (fixed effects)	4	1.29	1.03	1.62	0.028	0.894	
		Maximum likelihood	4	1.29	1.03	1.63	0.029		
		Simple median	4	1.32	1.00	1.74	0.050		
		Weighted median	4	1.32	1.02	1.71	0.038		
		MR Egger	4	1.21	0.44	3.32	0.748		0.907
Overall ovarian cancer	Tetradecanoylcarnitine	Inverse variance weighted (fixed effects)	2	0.80	0.66	0.97	0.020	0.168	/
		Maximum likelihood	2	0.80	0.66	0.97	0.022		
High grade serous ovarian cancer	Tetradecanoylcarnitine	Inverse variance weighted (fixed effects)	3	0.81	0.66	0.99	0.041	0.696	
		Maximum likelihood	3	0.81	0.66	0.99	0.042		
		Simple median	3	0.80	0.62	1.03	0.089		
		Weighted median	3	0.79	0.63	0.99	0.039		
		MR Egger	3	0.48	0.13	1.79	0.471		0.574
Low malignant potential ovarian cancer	Arginine	Inverse variance weighted (fixed effects)	7	0.63	0.42	0.95	0.028	0.871	
		Maximum likelihood	7	0.63	0.42	0.95	0.029		
		Simple median	7	0.69	0.39	1.22	0.201		
		Weighted median	7	0.57	0.34	0.93	0.025		
		MR Egger	7	0.53	0.27	1.04	0.124		0.537
Low malignant potential ovarian cancer	Dodecanoylcarnitine	Wald ratio	1	0.12	0.02	0.74	0.022	/	/
Low malignant potential ovarian cancer	Leucine	Inverse variance weighted (fixed effects)	4	4.25	1.22	14.83	0.023	0.064	
		Maximum likelihood	4	4.45	1.23	16.08	0.023		
		Simple median	4	4.84	0.91	25.83	0.065		
		Weighted median	4	4.35	0.82	23.06	0.084		
		MR Egger	4	0.19	0.00	99.92	0.659		0.416
Low malignant potential ovarian cancer	Octadecenoylcarnitine	Inverse variance weighted (fixed effects)	2	0.61	0.37	1.00	0.049	0.124	/
		Maximum likelihood	2	0.60	0.36	1.00	0.050		
Low malignant potential ovarian cancer	Threonine	Inverse variance weighted (fixed effects)	3	0.42	0.23	0.77	0.005	0.352	
		Maximum likelihood	3	0.41	0.22	0.77	0.005		
		Simple median	3	0.66	0.26	1.70	0.390		
		Weighted median	3	0.44	0.22	0.86	0.017		
		MR Egger	3	0.07	0.00	1.01	0.301		0.406
Invasive mucinous ovarian cancer	alpha-Aminoadipic acid	Inverse variance weighted (fixed effects)	2	2.09	1.02	4.28	0.045	0.811	/
		Maximum likelihood	2	2.09	1.00	4.35	0.049		
Invasive mucinous ovarian cancer	Creatinine	Inverse variance weighted (fixed effects)	12	0.45	0.24	0.84	0.012	0.666	
		Maximum likelihood	12	0.45	0.24	0.84	0.013		
		Simple median	12	0.41	0.18	0.95	0.038		
		Weighted median	12	0.43	0.19	0.99	0.046		
		MR Egger	12	0.05	0.00	1.00	0.079		0.175
Invasive mucinous ovarian cancer	Hexose	Wald ratio	1	2.51	1.05	6.02	0.039	/	/
Invasive mucinous ovarian cancer	Methionine	Wald ratio	1	0.23	0.06	0.89	0.033	/	/
Invasive mucinous ovarian cancer	Tetradecenoylcarnitine	Inverse variance weighted (fixed effects)	2	0.39	0.17	0.93	0.034	0.502	
		Maximum likelihood	2	0.39	0.16	0.95	0.038		
Clear cell ovarian cancer	Butyrylcarnitine	Inverse variance weighted (fixed effects)	2	0.62	0.40	0.95	0.029	0.116	/
		Maximum likelihood	2	0.62	0.40	0.95	0.030		
Clear cell ovarian cancer	Methioninesulfoxide	Wald ratio	1	0.28	0.09	0.85	0.025	/	/
Endometrioid ovarian cancer	Citrulline	Inverse variance weighted (fixed effects)	4	1.65	1.17	2.34	0.005	0.259	
		Maximum likelihood	4	1.66	1.17	2.38	0.005		
		Simple median	4	1.64	1.08	2.49	0.021		
		Weighted median	4	1.80	1.16	2.80	0.008		
		MR Egger	4	0.44	0.03	6.32	0.606		0.428
Endometrioid ovarian cancer	Tryptophan	Inverse variance weighted (fixed effects)	2	1.72	1.12	2.64	0.013	0.698	/
		Maximum likelihood	2	1.72	1.11	2.66	0.015		

### 3.2 Causal estimates of genetically predicted small molecule metabolic products on subtype ovarian cancers

**Figure 3:**
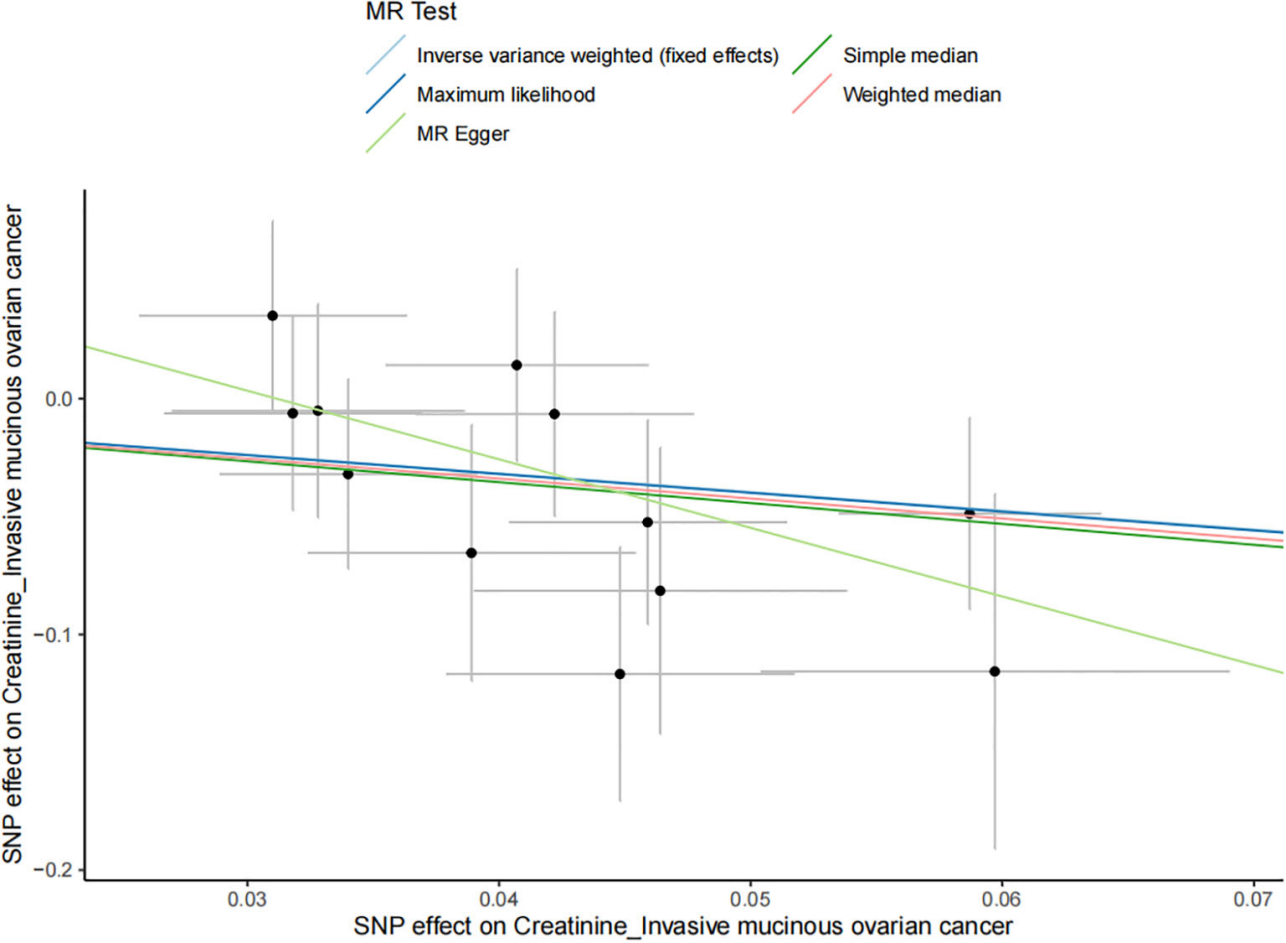
The negative effect of creatinine on the invasive mucinous ovarian cancer risk in the IVW analysis and it verified by Maximum likelihood approach and simple median method (all *P*<0.05).

**Figure 4:**
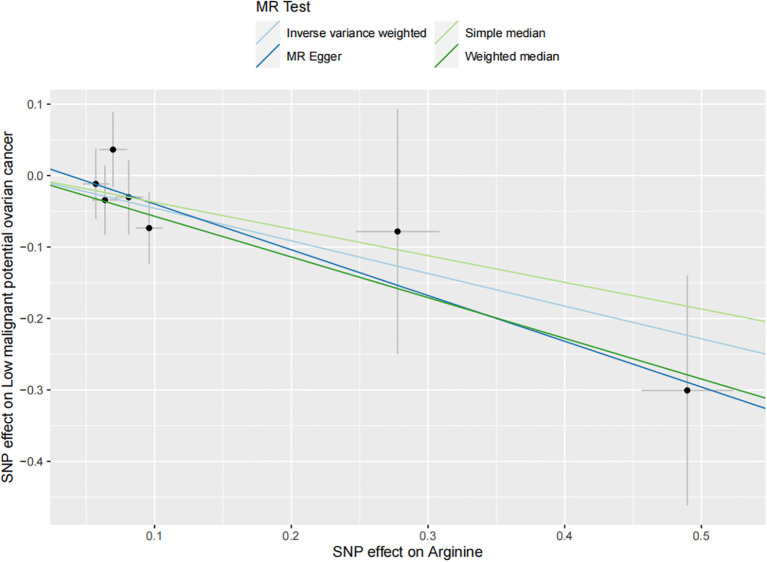
The inverse causal estimate between molecule metabolic products and the low malignant potential ovarian cancer (Increased arginine level may decrease the 46.7% risk of low malignant potential ovarian cancer. *P* =0.004).

**Figure 5:**
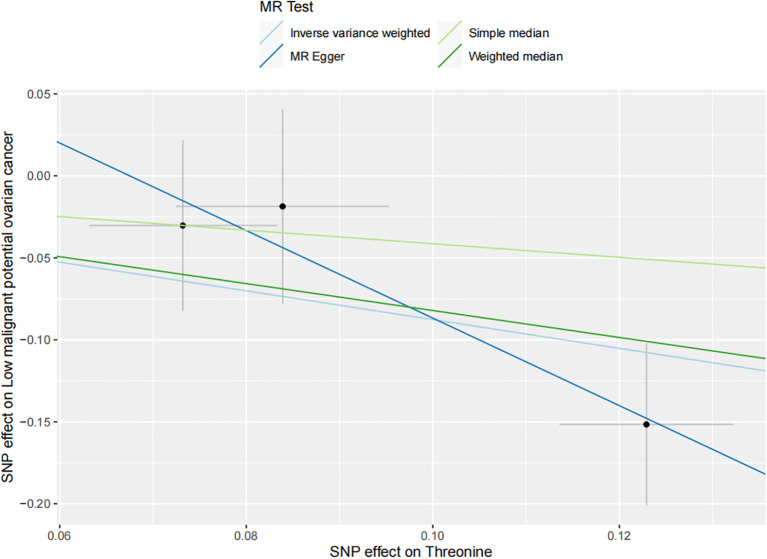
The inverse causal estimate between molecule metabolic products and the low malignant potential ovarian cancer. Threonine has a negative impact on the low malignant potential ovarian cancer (OR=0.42, 95% CI=0.23-0.77, *P* =0.004).

The results of all small molecule metabolic products on subtype ovarian cancers were presented in the **Supplementary Tables 4–8**. **Figure 2** presented the estimate from the MR analysis and suggested that a total of 15 small molecule metabolic products were related to the subtype ovarian cancers. Methionine sulfoxide was observed that associated clear cell ovarian cancer with dramatically reduced risk (OR=0.28, 95% CI=0.09-0.85, *P*=0.024). The similar causal association between tetradecanoylcarnitine and high grade serous ovarian cancer was detected. For endometrioid ovarian cancer, IVW method suggested genetically predicted Tryptophan would climb its 1.72-fold risk (95% CI=1.12-2.64, *P* =0.013). Five small molecule metabolic products were found that related to low malignant potential ovarian cancer and invasive mucinous ovarian cancer, respectively. For example, genetically predicted creatinine reduced the risk of invasive mucinous ovarian cancer, with estimates of IVW at 0.45 (95% CI=0.24-0.84, *P* =0.012; **Figure 3**). This causal relationship also was verified by Maximum likelihood approach and simple median method, while it did not attach a statistical significance in weighted median. Besides, arginine had a negative effect (OR=0.63, 95% CI=0.42-0.95, *P* =0.028; **Figure 4**) on low malignant potential ovarian cancer as well as threonine (OR=0.42, 95% CI=0.23-0.77, *P* =0.004; **Figure 5**). The pleiotropy test of Egger intercept suggested that there was no pleiotropy (*P*>0.05).

## 4 Discussion

OC remains one of the foremost gynecological malignancies with a significant global impact (5). Despite significant advancements in elucidating its etiology, the insidious onset of OC frequently results in advanced-stage diagnoses, underscoring the paramount importance of early detection biomarkers (5). This current study addresses this pressing clinical gap, harnessing the capabilities of Mendelian randomization to discern putative causal associations between small molecular metabolites and OC susceptibility. Significantly, this represents the inaugural effort of its nature to apply this methodology on comprehensive genetic datasets to assess these correlations. Overall, our findings provide novel insights into the complex metabolic underpinnings of OC.

In recent studies, several small-molecule metabolites have emerged as potential biomarkers for the early detection, risk stratification, and targeted prevention of OC (29).

In our comprehensive metabolic analysis, we identified a total of six metabolites significantly associated with the incidence of overall OC. Decreased risks were observed in association with the following metabolites: octadecandienylcarnitine, octadecenoylcarnitine, hexadecenoylcarnitine, tetradecanoylcarnitine, and methionine sulfoxide. Conversely, an elevated phenylalanine level was significantly associated with an augmented risk of overall OC. For HGSOC, tetradecanoylcarnitine was indicative of a reduced risk. In the context of LMPOC, the metabolites arginine, octadecenoylcarnitine, and threonine were inversely correlated with risk, while an increase in leucine levels showed a heightened risk. Regarding IMOC, a diminished risk was noted in conjunction with creatinine, decenoylcarnitine, methionine, and tetradecenoylcarnitine. Conversely, the levels of alpha-Aminoadipic acid and hexose were positively correlated with increased risk. For CCOC, butyrylcarnitine was a marker of reduced risk, whereas increased methioninesulfoxide levels were linked to heightened risk. In EndoOC, citrulline and tryptophan were indicative of a reduced risk. However, elevated glycine levels were observed to increase the risk.

Notably, an increase in genetically predicted levels of methionine sulfoxide correlated with a 31% reduction in the risk of overall OC. Methioninesulfoxide is generated via oxidation of methionine residues in proteins and serves as a biomarker of oxidative damage (30, 31). Accumulating evidence suggests that methioninesulfoxide reductases act as antioxidant repair enzymes to revert oxidized methionines and defend against oxidative stress (32). Our results indicate that methioninesulfoxide may confer protection against OC through antioxidant effects. Genetically elevated tryptophan levels were associated with a 1.72-fold increased risk of endometrioid OC in our analysis. Tryptophan is an essential amino acid and precursor for bioactive molecules like serotonin and melatonin (33). Previous studies have found that changes in tryptophan metabolism in tumors are often accompanied by abnormal expression of tryptophan-related enzyme genes. Among the observed alterations, variations in the expression of genes associated with indoleamine 2,3-dioxygenase and tryptophan 2,3-dioxygenase emerge as the most prevalent (34). In the human body, tryptophan has three metabolic pathways. Catabolism of tryptophan through the kynurenine pathway produces immunosuppressive metabolites and has been implicated in facilitating tumor immune evasion (35). We also found that higher predicted arginine and threonine were associated with 37% and 58% decreased risks of low malignant potential ovarian cancer, respectively. Threonine serves several functions. One of its primary roles is in the synthesis of mucin, a substance crucial for maintaining intestinal integrity and function (36). Additionally, threonine plays a significant part in immune function, contributing to the body’s defense mechanisms (36). It is also involved in the phosphorylation and glycosylation of proteins, processes that are essential for protein function and stability (36). Lastly, threonine in the synthesis of glycine, an amino acid that has numerous roles in the body (36). Arginine is a semi-essential amino acid and substrate for nitric oxide synthesis (37). Nitric oxide is a ubiquitous messenger molecule with dichotomous pro- and anti-tumorigenic actions (38). Further research should clarify the role of arginine metabolism in OC.

We found that higher genetically predicted levels of several acylcarnitine species (tetradecanoylcarnitine, hexadecenoylcarnitine, octadecenoylcarnitine, octadecadienyl carnitine) were associated with decreased risks of OC. Acylcarnitines are generated via esterification of fatty acids and shuttle lipids into the mitochondrial matrix for β-oxidation (39). Reduced acylcarnitine levels imply impaired fatty acid oxidation and mitochondrial dysfunction, which are implicated in OC (40, 41). Additionally, an association was observed between tetradecanoylcarnitine and a diminished risk of HGSOC. Carnitine palmitoyltransferase-1 (CPT-1), positioned on the outer membrane of mitochondria, principally catalyzes the conversion of long-chain fatty acyl-CoA and carnitine to fatty acyl carnitine. This conversion represents the preliminary rate-limiting phase in the mitochondrial oxidation of fatty acids (42). CPT-1 downregulation induces a glycolytic shift in cancer cells (43, 44). Targeting CPT-1 may thus restrain HGSOC growth by blocking fatty acid oxidation.

Regarding IMOC, a diminished risk was noted in conjunction with higher genetically predicted levels of creatinine, decenoylcarnitine, methionine, and tetradecenoylcarnitine. This suggests impairments in pathways related to these metabolites, such as fatty acid oxidation, antioxidant defenses, and nitrogen metabolism, may contribute to the development of this ovarian cancer subtype (45). Creatinine is a breakdown product of creatine phosphate in muscle and is usually produced at a fairly constant rate by the body (46). The lower creatinine levels associated with higher ovarian cancer risk may indicate impaired muscle metabolism or greater catabolism in this patient population. This is also consistent with previous studies (47). Decenoylcarnitine is a medium-chain fatty acid derivative involved in transporting fatty acids into the mitochondria for beta-oxidation. The reduced cancer risk with higher decenoylcarnitine hints at a possible role of improved fatty acid metabolism in protecting against ovarian carcinogenesis. Methionine is an essential amino acid that serves as a precursor for protein synthesis and other important biomolecules like cysteine and taurine (48). The inverse association between methionine and IMOC risk is consistent with its known functions in maintaining genomic stability and redox homeostasis through DNA methylation and antioxidant systems (49). Higher methionine levels may suppress ovarian tumorigenesis through these mechanisms. In contrast, elevated levels of alpha-aminoadipic acid and hexose were associated with increased IMOC risk. Alpha-aminoadipic acid is an intermediate in lysine degradation, while hexoses are simple sugars. The positive correlations indicate dysregulated lysine catabolism and carbohydrate metabolism could play pathogenic roles. Alpha-aminoadipic acid is an intermediate in lysine metabolism and a marker of oxidative stress that may accumulate with possible lysine deficiency or dysfunction in this pathway (50). Hexose represents the combined pool of six-carbon sugars including glucose and fructose (51). The increased ovarian cancer risk with higher hexose levels may stem from greater availability of glycolytic intermediates to fuel rapid tumor growth (52). This fits with existing evidence on the key role of glycolytic metabolism in ovarian cancer progression (53).

For CCOC, elevated levels of butyrylcarnitine were associated with a reduced risk. Butyrylcarnitine is involved in fatty acid metabolism, and previous studies have found fatty acid oxidation pathways to be downregulated in CCOC (42). The reduced butyrylcarnitine levels observed here likely reflect impairments in this metabolic pathway that may promote CCOC pathogenesis.

For EndoOC, lower citrulline and tryptophan levels were indicative of a reduced risk. Citrulline is a key intermediate in the urea cycle, while tryptophan is an essential amino acid. Past work indicates both these metabolites are involved in maintaining immune homeostasis (54). The decreased levels seen here imply EndoOC risk may rise when immune regulation is disrupted. Meanwhile, elevated glycine was tied to heightened EndoOC risk. Glycine serves as a precursor for glutathione, a key antioxidant. The increased glycine levels likely reflect a compensatory response to mitigate oxidative damage that otherwise enables EndoOC pathogenesis (55).

This study has several strengths. We employed mendelian randomization–a powerful genetic epidemiological tool. It utilizes SNPs closely tied to the exposure, serving as IVs to uncover potential causal links between the exposure and the outcome. Genotypes are believed to be randomly distributed during gametogenesis. Thus, using the IVs model addresses many confounding challenges in observational research, especially when biases arise from unmeasured confounders. Thanks to the inherent randomness of genotypic distribution, MR helps counter potential confounding and reverse causality. We drew from the most extensive GWAS dataset on OC, enhancing our statistical validity.

This study presents several limitations. Primarily, the cohort was confined to individuals of European descent, which minimizes potential bias from population stratification but may not adequately capture the diversity of SNP redundancy, particularly given the unavailability of the original dataset. Moreover, while our results suggest a potential causal linkage between small molecular metabolites and OC, the clarity of data presentation regarding the relationships between different metabolites could be improved for the reader’s comprehension and comparative analysis. Recognizing these issues, we assert the necessity for subsequent investigations, including experimental validation in a broader array of populations and in-depth exploration of the underlying biological mechanisms. Such research will not only corroborate our findings but also illuminate the complex metabolic interactions associated with OC, offering substantial contributions to the oncological community’s understanding of this disease.

## 5 Conclusion

In this MR analysis, we observed putatively causal associations between certain small molecule metabolites and the risk of OC. Our observations underscore the potential for metabolic profiling in risk stratification, early diagnosis, and individualized preventive strategies for OC. These findings not only enhance our etiological understanding but also pave the way for subsequent investigations into targeting anomalous metabolic pathways in OC.

## Data availability statement

The original contributions presented in the study are included in the article/**Supplementary Material**. Further inquiries can be directed to the corresponding author.

## Author contributions

XC: Conceptualization, Investigation, Methodology, Writing – original draft. SL: Formal analysis, Project administration, Validation, Visualization, Writing – original draft. LH: Formal analysis, Methodology, Project administration, Validation, Writing – original draft, Writing – review & editing.
