# Supplementary material for: Mendelian randomization analysis to elucidate the causal relationship between small molecule metabolites and ovarian cancer risk
Source: Front Oncol. 2023 Nov 28;13:1291033. doi: 10.3389/fonc.2023.1291033 (PMC10713731; doi:10.3389/fonc.2023.1291033)
Supplement: Supplementary file 1 [file Table_1.docx]

**Supplementary Material**

**Supplementary Table 1. Details of the genome-wide association studies and datasets used in this study.**

**Supplementary Table 2. Characteristics of the genetic variants associated with the Small Molecule Metabolites.**

**Supplementary Table 3. The association between exposure and overall ovarian cancer in the MR analysis.**

**Supplementary Table 4. The association between exposure and high grade serous ovarian cancer.**

**Supplementary Table 5. The association between exposure and Low malignant potential ovarian cancer.**

**Supplementary Table 6. The association between exposure and Invasive mucinous ovarian cancer.**

**Supplementary Table 7. The association between exposure and Clear cell ovarian cancer.**

**Supplementary Table 8. The association between exposure and Endometrioid ovarian cancer.**

| **Supplementary Table 1**. Details of the genome-wide association studies and datasets used in this study. | | | | | |
| --- | --- | --- | --- | --- | --- |
| Trait | ID | Case | Control | PMID | Link |
| Overall ovarian cancer | Ieu-a-1120 | 25,509 | 40,941 | 28346442 | <https://gwas.mrcieu.ac.uk/> |
| High grade serous ovarian cancer | Ieu-a-1121 | 13,037 | 40,941 |  |  |
| Low malignant potential ovarian cancer | Ieu-a-1122 | 3,103 | 40,941 |  |  |
| Invasive mucinous ovarian cancer | Ieu-a-1123 | 1,417 | 40,941 |  |  |
| Clear cell ovarian cancer | Ieu-a-1124 | 1,366 | 40,941 |  |  |
| Endometrioid ovarian cancer | Ieu-a-1125 | 2810 | 40,941 |  |  |

| **Supplementary Table** **2** Characteristics of the genetic variants associated with the Small Molecule Metabolites. | | | | | | | | | | |
| --- | --- | --- | --- | --- | --- | --- | --- | --- | --- | --- |
| Metabolite | Class | SNP | Chr | Position | Effect allele | EAF | Beta | StdErr | *P*-value | F-statistics |
| Acetylcarnitine | Acylcarnitines | rs1171617 | 10 | 61467182 | t | 0.7642 | 0.2724 | 0.0109 | 9.96E-139 | 624.54 |
| Acetylcarnitine | Acylcarnitines | rs662138 | 6 | 160564476 | c | 0.8162 | 0.101 | 0.0119 | 1.61E-17 | 72.04 |
| Acetylcarnitine | Acylcarnitines | rs272869 | 5 | 131677997 | a | 0.3713 | 0.1172 | 0.0095 | 4.89E-35 | 152.20 |
| Acetylcarnitine | Acylcarnitines | rs149235996 | 5 | 150719909 | a | 0.9893 | 0.39 | 0.0459 | 1.96E-17 | 72.19 |
| Acetylornithine | Biogenic amines | rs13538 | 2 | 73868328 | a | 0.7755 | 0.8457 | 0.0101 | 9.52E-1525 | 7011.16 |
| Acetylornithine | Biogenic amines | rs140139389 | 5 | 1163328 | t | 0.9948 | 0.7525 | 0.0668 | 1.93E-29 | 126.90 |
| Alanine | Amino acids | rs2638314 | 12 | 56866334 | a | 0.1827 | 0.0806 | 0.0072 | 2.85E-29 | 125.32 |
| Alanine | Amino acids | rs1780638 | 22 | 19177522 | g | 0.6334 | 0.0322 | 0.0057 | 2.13E-08 | 31.91 |
| Alanine | Amino acids | rs1440581 | 4 | 89226422 | t | 0.466 | 0.0332 | 0.0056 | 2.81E-09 | 35.15 |
| Alanine | Amino acids | rs1260326 | 2 | 27730940 | t | 0.3978 | 0.0523 | 0.0057 | 2.22E-20 | 84.19 |
| Alanine | Amino acids | rs7412 | 19 | 45412079 | t | 0.0822 | 0.0645 | 0.0101 | 1.93E-10 | 40.78 |
| Alanine | Amino acids | rs10211524 | 2 | 65208074 | g | 0.5709 | 0.0416 | 0.0057 | 3.82E-13 | 53.26 |
| Alanine | Amino acids | rs4554975 | 12 | 47201814 | a | 0.4399 | 0.0573 | 0.0057 | 1.65E-23 | 101.06 |
| Alanine | Amino acids | rs8061221 | 16 | 70078960 | a | 0.7434 | 0.0621 | 0.0064 | 1.71E-22 | 94.15 |
| Alanine | Amino acids | rs964184 | 11 | 116648917 | c | 0.8674 | 0.0477 | 0.0082 | 4.81E-09 | 33.84 |
| Alanine | Amino acids | rs2016939 | 1 | 207240289 | a | 0.59 | 0.053 | 0.0056 | 5.51E-21 | 89.57 |
| Alanine | Amino acids | rs17122585 | 12 | 59909653 | g | 0.115 | 0.0574 | 0.0088 | 5.45E-11 | 42.55 |
| Alanine | Amino acids | rs13232120 | 7 | 72983310 | t | 0.1214 | 0.0511 | 0.0085 | 1.76E-09 | 36.14 |
| Alanine | Amino acids | rs17712208 | 1 | 214150445 | a | 0.0351 | 0.1183 | 0.0155 | 2.53E-14 | 58.25 |
| Alanine | Amino acids | rs2168101 | 11 | 8255408 | c | 0.6878 | 0.0353 | 0.0064 | 3.49E-08 | 30.42 |
| Alanine | Amino acids | rs4237150 | 9 | 4290085 | c | 0.4035 | 0.0316 | 0.0057 | 2.60E-08 | 30.73 |
| Alanine | Amino acids | rs2306386 | 8 | 145742879 | t | 0.4982 | 0.0322 | 0.0055 | 6.63E-09 | 34.28 |
| alpha-Aminoadipic acid | Biogenic amines | rs75096272 | 5 | 36630209 | g | 0.9687 | 0.2352 | 0.0325 | 4.71E-13 | 52.37 |
| alpha-Aminoadipic acid | Biogenic amines | rs9424148 | 10 | 12146858 | a | 0.7905 | 0.0979 | 0.0141 | 3.59E-12 | 48.21 |
| Arginine | Amino acids | rs715 | 2 | 211543055 | t | 0.6877 | 0.0811 | 0.01 | 4.92E-16 | 65.77 |
| Arginine | Amino acids | rs7199750 | 16 | 68351859 | g | 0.7098 | 0.0599 | 0.0101 | 3.04E-09 | 35.17 |
| Arginine | Amino acids | rs17788484 | 6 | 131894358 | c | 0.9796 | 0.4896 | 0.0334 | 8.76E-49 | 214.88 |
| Arginine | Amino acids | rs17666239 | 12 | 47194757 | a | 0.0675 | 0.1037 | 0.0183 | 1.38E-08 | 32.11 |
| Arginine | Amino acids | rs11085824 | 19 | 13001547 | a | 0.6289 | 0.096 | 0.0096 | 1.08E-23 | 100.00 |
| Arginine | Amino acids | rs56335308 | 8 | 17419461 | g | 0.9764 | 0.2777 | 0.0303 | 4.65E-20 | 84.00 |
| Arginine | Amino acids | rs12499429 | 4 | 144811067 | a | 0.3108 | 0.0696 | 0.0099 | 2.15E-12 | 49.43 |
| Arginine | Amino acids | rs10189479 | 2 | 219287276 | a | 0.4247 | 0.057 | 0.0093 | 8.97E-10 | 37.57 |
| Arginine | Amino acids | rs592423 | 6 | 139840693 | a | 0.444 | 0.0637 | 0.0092 | 5.11E-12 | 47.94 |
| Asparagine | Amino acids | rs1406384 | 11 | 62083530 | t | 0.8834 | 0.1282 | 0.0142 | 2.31E-19 | 81.51 |
| Asparagine | Amino acids | rs12587599 | 14 | 104575130 | t | 0.1396 | 0.4851 | 0.0132 | 9.64E-295 | 1350.56 |
| Asparagine | Amino acids | rs13106192 | 4 | 178362122 | g | 0.3577 | 0.0563 | 0.0096 | 3.87E-09 | 34.39 |
| Asparagine | Amino acids | rs1260326 | 2 | 27730940 | c | 0.5993 | 0.0618 | 0.0093 | 2.89E-11 | 44.16 |
| Asparagine | Amino acids | rs17345286 | 7 | 97490022 | t | 0.726 | 0.0775 | 0.0103 | 5.34E-14 | 56.61 |
| Aspartate | Amino acids | rs715 | 2 | 211543055 | t | 0.6877 | 0.0698 | 0.01 | 3.36E-12 | 48.72 |
| Aspartate | Amino acids | rs4690522 | 4 | 178361051 | c | 0.6414 | 0.1721 | 0.0095 | 1.06E-72 | 328.18 |
| Aspartate | Amino acids | rs7964859 | 12 | 102220783 | c | 0.7146 | 0.1229 | 0.0102 | 2.31E-33 | 145.18 |
| Butyrylcarnitine | Acylcarnitines | rs3916 | 12 | 121177272 | c | 0.2557 | 0.8074 | 0.0097 | 2.68E-1500 | 6928.42 |
| Butyrylcarnitine | Acylcarnitines | rs1171617 | 10 | 61467182 | t | 0.7642 | 0.196 | 0.0109 | 4.85E-72 | 323.34 |
| Butyrylcarnitine | Acylcarnitines | rs662138 | 6 | 160564476 | c | 0.816 | 0.232 | 0.0119 | 5.76E-85 | 380.09 |
| Butyrylcarnitine | Acylcarnitines | rs272884 | 5 | 131668654 | g | 0.3786 | 0.0787 | 0.0095 | 1.16E-16 | 68.63 |
| Butyrylcarnitine | Acylcarnitines | rs2291449 | 15 | 76579234 | g | 0.0901 | 0.1556 | 0.0161 | 3.49E-22 | 93.40 |
| Butyrylcarnitine | Acylcarnitines | rs113488591 | 11 | 134128923 | g | 0.0259 | 0.2662 | 0.0291 | 6.05E-20 | 83.68 |
| Carnitine | Acylcarnitines | rs1169299 | 12 | 121429194 | c | 0.46 | 0.0563 | 0.009 | 3.86E-10 | 39.13 |
| Carnitine | Acylcarnitines | rs1171617 | 10 | 61467182 | t | 0.7642 | 0.4333 | 0.0105 | 6.53E-372 | 1702.94 |
| Carnitine | Acylcarnitines | rs274551 | 5 | 131727680 | c | 0.8349 | 0.1758 | 0.012 | 1.88E-48 | 214.62 |
| Carnitine | Acylcarnitines | rs77010315 | 5 | 150723155 | c | 0.9893 | 0.5839 | 0.0444 | 1.61E-39 | 172.95 |
| Carnitine | Acylcarnitines | rs10466245 | 10 | 46031747 | g | 0.7651 | 0.0883 | 0.0105 | 4.81E-17 | 70.72 |
| Carnitine | Acylcarnitines | rs853358 | 6 | 14131244 | t | 0.213 | 0.103 | 0.0111 | 1.48E-20 | 86.11 |
| Carnitine | Acylcarnitines | rs12715455 | 3 | 53035943 | t | 0.3608 | 0.0716 | 0.0097 | 1.24E-13 | 54.49 |
| Carnitine | Acylcarnitines | rs111653425 | 17 | 19474875 | t | 0.0111 | 0.2755 | 0.0435 | 2.31E-10 | 40.11 |
| Citrulline | Amino acids | rs1509820 | 2 | 211386484 | a | 0.5403 | 0.0824 | 0.009 | 7.66E-20 | 83.82 |
| Citrulline | Amino acids | rs612169 | 9 | 136143442 | a | 0.6743 | 0.0528 | 0.0096 | 3.85E-08 | 30.25 |
| Citrulline | Amino acids | rs11243372 | 9 | 133324400 | a | 0.54 | 0.0972 | 0.0092 | 3.25E-26 | 111.62 |
| Citrulline | Amino acids | rs17681684 | 17 | 9792768 | a | 0.3179 | 0.0842 | 0.0098 | 6.62E-18 | 73.82 |
| Citrulline | Amino acids | rs60837490 | 8 | 10579523 | a | 0.075 | 0.1325 | 0.0175 | 4.32E-14 | 57.33 |
| Creatinine | Biogenic amines | rs1047891 | 2 | 211540507 | a | 0.3153 | 0.0459 | 0.0055 | 3.99E-17 | 69.65 |
| Creatinine | Biogenic amines | rs3119311 | 6 | 160693107 | c | 0.1364 | 0.0464 | 0.0074 | 3.66E-10 | 39.32 |
| Creatinine | Biogenic amines | rs1260326 | 2 | 27730940 | c | 0.6024 | 0.0407 | 0.0052 | 3.01E-15 | 61.26 |
| Creatinine | Biogenic amines | rs1145093 | 15 | 45649813 | a | 0.3736 | 0.0587 | 0.0052 | 2.98E-29 | 127.43 |
| Creatinine | Biogenic amines | rs1065853 | 19 | 45413233 | t | 0.082 | 0.0597 | 0.0093 | 1.41E-10 | 41.21 |
| Creatinine | Biogenic amines | rs3812035 | 5 | 176817143 | t | 0.3191 | 0.0422 | 0.0055 | 1.03E-14 | 58.87 |
| Creatinine | Biogenic amines | rs11657044 | 17 | 59450105 | t | 0.1684 | 0.0448 | 0.0069 | 7.61E-11 | 42.16 |
| Creatinine | Biogenic amines | rs5020545 | 4 | 77414988 | t | 0.4458 | 0.034 | 0.0051 | 3.63E-11 | 44.44 |
| Creatinine | Biogenic amines | rs1014680 | 2 | 217672824 | a | 0.5685 | 0.031 | 0.0053 | 4.03E-09 | 34.21 |
| Creatinine | Biogenic amines | rs12922822 | 16 | 20367645 | c | 0.8171 | 0.0389 | 0.0065 | 2.49E-09 | 35.82 |
| Creatinine | Biogenic amines | rs4390625 | 17 | 37620347 | g | 0.7444 | 0.0328 | 0.0058 | 1.71E-08 | 31.98 |
| Creatinine | Biogenic amines | rs1544459 | 7 | 77417584 | c | 0.4555 | 0.0318 | 0.0051 | 3.74E-10 | 38.88 |
| Decanoylcarnitine | Acylcarnitines | rs7552404 | 1 | 76135946 | a | 0.692 | 0.2101 | 0.01 | 5.85E-99 | 441.42 |
| Decanoylcarnitine | Acylcarnitines | rs8396 | 4 | 159630817 | t | 0.6991 | 0.1888 | 0.01 | 1.54E-79 | 356.45 |
| Decanoylcarnitine | Acylcarnitines | rs924135 | 16 | 16123459 | t | 0.61 | 0.087 | 0.0095 | 4.62E-20 | 83.87 |
| Decenoylcarnitine | Acylcarnitines | rs7552404 | 1 | 76135946 | a | 0.699 | 0.1785 | 0.0159 | 2.25E-29 | 126.03 |
| Decenoylcarnitine | Acylcarnitines | rs8396 | 4 | 159630817 | t | 0.6978 | 0.1252 | 0.0158 | 2.60E-15 | 62.79 |
| Decenoylcarnitine | Acylcarnitines | rs11075289 | 16 | 16098368 | c | 0.68 | 0.0895 | 0.0156 | 9.74E-09 | 32.92 |
| Dodecanoylcarnitine | Acylcarnitines | rs2070630 | 4 | 159644494 | g | 0.6978 | 0.1085 | 0.01 | 1.97E-27 | 117.72 |
| Dodecanoylcarnitine | Acylcarnitines | rs156322 | 5 | 131653925 | c | 0.2962 | 0.058 | 0.01 | 6.19E-09 | 33.64 |
| Dodecanoylcarnitine | Acylcarnitines | rs924135 | 16 | 16123459 | t | 0.61 | 0.0922 | 0.0094 | 1.13E-22 | 96.21 |
| Dodecenoylcarnitine | Acylcarnitines | rs12921623 | 16 | 16096133 | c | 0.6133 | 0.1025 | 0.0152 | 1.51E-11 | 45.47 |
| Glutamine | Amino acids | rs2657879 | 12 | 56865338 | a | 0.8172 | 0.2226 | 0.0074 | 8.39E-198 | 904.87 |
| Glutamine | Amino acids | rs1260326 | 2 | 27730940 | c | 0.6022 | 0.0672 | 0.0058 | 1.37E-30 | 134.24 |
| Glutamine | Amino acids | rs7078003 | 10 | 99359412 | t | 0.1783 | 0.0818 | 0.0075 | 1.03E-27 | 118.96 |
| Glutamine | Amino acids | rs17602430 | 12 | 47185518 | t | 0.0682 | 0.1217 | 0.0113 | 7.17E-27 | 115.99 |
| Glutamine | Amino acids | rs7587672 | 2 | 191723275 | g | 0.748 | 0.0593 | 0.0066 | 3.46E-19 | 80.73 |
| Glutamine | Amino acids | rs13281892 | 8 | 17372697 | a | 0.6722 | 0.0473 | 0.0062 | 2.20E-14 | 58.20 |
| Glutamine | Amino acids | rs17558901 | 12 | 59915849 | a | 0.1147 | 0.0587 | 0.0091 | 8.95E-11 | 41.61 |
| Glutamine | Amino acids | rs34430945 | 7 | 73043665 | c | 0.1272 | 0.0712 | 0.0086 | 1.43E-16 | 68.54 |
| Glutamine | Amino acids | rs79687284 | 1 | 214150821 | c | 0.035 | 0.1273 | 0.0162 | 3.46E-15 | 61.75 |
| Glutamine | Amino acids | rs2168101 | 11 | 8255408 | c | 0.6878 | 0.0422 | 0.0066 | 1.87E-10 | 40.88 |
| Glycine | Amino acids | rs715 | 2 | 211543055 | c | 0.3123 | 0.4441 | 0.0062 | 1.17e-1107 | 5130.72 |
| Glycine | Amino acids | rs4646961 | 1 | 76217169 | a | 0.3039 | 0.0476 | 0.0063 | 2.86E-14 | 57.09 |
| Glycine | Amino acids | rs561931 | 1 | 120254506 | g | 0.5876 | 0.033 | 0.0059 | 1.66E-08 | 31.28 |
| Glycine | Amino acids | rs543159 | 6 | 160776017 | a | 0.4743 | 0.035 | 0.0058 | 1.25E-09 | 36.41 |
| Glycine | Amino acids | rs4947534 | 7 | 56079094 | c | 0.7504 | 0.0722 | 0.0067 | 1.83E-27 | 116.12 |
| Glycine | Amino acids | rs17591030 | 9 | 6550024 | c | 0.713 | 0.0805 | 0.0064 | 7.49E-36 | 158.21 |
| Glycine | Amino acids | rs9987289 | 8 | 9183358 | a | 0.0919 | 0.1237 | 0.01 | 2.60E-35 | 153.02 |
| Glycine | Amino acids | rs9923732 | 16 | 81110903 | a | 0.9218 | 0.1188 | 0.0107 | 1.65E-28 | 123.27 |
| Glycine | Amino acids | rs12297321 | 12 | 47109387 | t | 0.158 | 0.0478 | 0.0079 | 1.14E-09 | 36.61 |
| Glycine | Amino acids | rs2545801 | 5 | 176841339 | c | 0.7482 | 0.0423 | 0.0066 | 1.89E-10 | 41.08 |
| Glycine | Amino acids | rs676996 | 9 | 136146077 | t | 0.6911 | 0.0399 | 0.0063 | 1.75E-10 | 40.11 |
| Glycine | Amino acids | rs9862438 | 3 | 125910381 | t | 0.4178 | 0.0582 | 0.0059 | 3.23E-23 | 97.31 |
| Glycine | Amino acids | rs28601761 | 8 | 126500031 | g | 0.4159 | 0.0634 | 0.006 | 5.46E-26 | 111.65 |
| Glycine | Amino acids | rs10740134 | 10 | 65315433 | t | 0.5167 | 0.038 | 0.0057 | 3.59E-11 | 44.44 |
| Glycine | Amino acids | rs8078686 | 17 | 45735706 | c | 0.5046 | 0.0348 | 0.0058 | 1.53E-09 | 36.00 |
| Hexadecanoylcarnitine | Acylcarnitines | rs1171617 | 10 | 61467182 | t | 0.7642 | 0.0959 | 0.0108 | 4.72E-19 | 78.85 |
| Hexadecanoylcarnitine | Acylcarnitines | rs72939920 | 6 | 110762453 | a | 0.7601 | 0.2035 | 0.0106 | 1.97E-82 | 368.57 |
| Hexadecanoylcarnitine | Acylcarnitines | rs272883 | 5 | 131668698 | a | 0.2957 | 0.1222 | 0.0099 | 3.74E-35 | 152.36 |
| Hexadecenoylcarnitine | Acylcarnitines | rs12210538 | 6 | 110760008 | a | 0.7589 | 0.1043 | 0.0136 | 2.01E-14 | 58.82 |
| Hexanoylcarnitine | Acylcarnitines | rs1171615 | 10 | 61469090 | t | 0.7636 | 0.1592 | 0.0173 | 3.74E-20 | 84.68 |
| Hexanoylcarnitine | Acylcarnitines | rs77931234 | 1 | 76226846 | g | 0.0095 | 1.5271 | 0.0812 | 6.98E-79 | 353.69 |
| Hexanoylcarnitine | Acylcarnitines | rs17843929 | 4 | 159637113 | c | 0.7286 | 0.142 | 0.0164 | 4.65E-18 | 74.97 |
| Hexose | Hexose | rs10830963 | 11 | 92708710 | g | 0.2792 | 0.0987 | 0.0158 | 4.57E-10 | 39.02 |
| Hexose | Hexose | rs560887 | 2 | 169763148 | c | 0.7055 | 0.0996 | 0.0154 | 1.08E-10 | 41.83 |
| Histidine | Amino acids | rs715 | 2 | 211543055 | t | 0.6875 | 0.0538 | 0.0063 | 1.34E-17 | 72.93 |
| Histidine | Amino acids | rs61937878 | 12 | 96371731 | t | 0.0064 | 0.855 | 0.0369 | 7.43E-119 | 536.88 |
| Histidine | Amino acids | rs3733402 | 4 | 187158034 | a | 0.5111 | 0.0725 | 0.0058 | 4.09E-36 | 156.25 |
| Histidine | Amino acids | rs780093 | 2 | 27742603 | c | 0.6134 | 0.0463 | 0.0059 | 4.49E-15 | 61.58 |
| Histidine | Amino acids | rs2545801 | 5 | 176841339 | c | 0.7483 | 0.0711 | 0.0067 | 2.01E-26 | 112.61 |
| Histidine | Amino acids | rs635634 | 9 | 136155000 | c | 0.8171 | 0.0493 | 0.0075 | 3.93E-11 | 43.21 |
| Histidine | Amino acids | rs5030062 | 3 | 186454180 | c | 0.3731 | 0.0493 | 0.006 | 1.40E-16 | 67.51 |
| Histidine | Amino acids | rs35291299 | 14 | 21515620 | g | 0.1221 | 0.0633 | 0.0089 | 1.05E-12 | 50.59 |
| Isoleucine | Amino acids | rs7298123 | 12 | 56860073 | c | 0.1998 | 0.0348 | 0.0061 | 1.24E-08 | 32.55 |
| Isoleucine | Amino acids | rs1440581 | 4 | 89226422 | c | 0.5342 | 0.0437 | 0.0049 | 6.14E-19 | 79.54 |
| Isoleucine | Amino acids | rs12149660 | 16 | 70309237 | g | 0.8872 | 0.0644 | 0.0078 | 1.25E-16 | 68.17 |
| Isoleucine | Amino acids | rs493841 | 19 | 49301877 | t | 0.512 | 0.0352 | 0.005 | 1.94E-12 | 49.56 |
| Kynurenine | Biogenic amines | rs4843718 | 16 | 87878476 | a | 0.2518 | 0.197 | 0.0103 | 2.42E-81 | 365.81 |
| Kynurenine | Biogenic amines | rs653178 | 12 | 112007756 | c | 0.4768 | 0.1075 | 0.009 | 8.42E-33 | 142.67 |
| Kynurenine | Biogenic amines | rs10085935 | 8 | 39806267 | c | 0.6258 | 0.0887 | 0.0093 | 1.60E-21 | 90.97 |
| Kynurenine | Biogenic amines | rs61825638 | 1 | 241713402 | t | 0.2419 | 0.1127 | 0.0105 | 7.37E-27 | 115.20 |
| Leucine | Amino acids | rs7678928 | 4 | 89222827 | t | 0.4658 | 0.0483 | 0.005 | 7.09E-22 | 93.32 |
| Leucine | Amino acids | rs4253272 | 4 | 187163614 | t | 0.5103 | 0.0307 | 0.005 | 6.91E-10 | 37.70 |
| Leucine | Amino acids | rs1260326 | 2 | 27730940 | t | 0.3976 | 0.0285 | 0.0051 | 1.71E-08 | 31.23 |
| Leucine | Amino acids | rs12149660 | 16 | 70309237 | g | 0.8873 | 0.0668 | 0.0079 | 2.71E-17 | 71.50 |
| Leucine | Amino acids | rs964184 | 11 | 116648917 | c | 0.8674 | 0.0635 | 0.0073 | 3.11E-18 | 75.67 |
| Leucine | Amino acids | rs261334 | 15 | 58726744 | c | 0.7919 | 0.0463 | 0.0061 | 4.55E-14 | 57.61 |
| Lysine | Amino acids | rs2657880 | 12 | 56863770 | c | 0.1797 | 0.0728 | 0.0119 | 8.75E-10 | 37.43 |
| Lysine | Amino acids | rs8056893 | 16 | 68304392 | a | 0.7316 | 0.1945 | 0.0103 | 1.00E-79 | 356.59 |
| Lysine | Amino acids | rs2608920 | 6 | 131873703 | t | 0.7743 | 0.0707 | 0.011 | 1.15E-10 | 41.31 |
| Lysine | Amino acids | rs7005693 | 8 | 49987965 | c | 0.535 | 0.1107 | 0.0092 | 1.32E-33 | 144.78 |
| Lysine | Amino acids | rs138373837 | 5 | 36219710 | t | 0.0235 | 0.3005 | 0.0301 | 2.07E-23 | 99.67 |
| Lysine | Amino acids | rs2517237 | 8 | 17375959 | g | 0.1976 | 0.0977 | 0.0115 | 1.56E-17 | 72.18 |
| Lysine | Amino acids | rs1059263 | 14 | 100769367 | c | 0.6914 | 0.0667 | 0.0101 | 3.44E-11 | 43.61 |
| Lysine | Amino acids | rs11554714 | 22 | 37407109 | g | 0.9642 | 0.182 | 0.0246 | 1.41E-13 | 54.74 |
| Lysine | Amino acids | rs7658095 | 4 | 139927828 | g | 0.6901 | 0.0678 | 0.01 | 1.10E-11 | 45.97 |
| Methionine | Amino acids | rs6891672 | 5 | 90243899 | t | 0.2991 | 0.0648 | 0.0098 | 3.32E-11 | 43.72 |
| Methioninesulfoxide | Biogenic amines | rs10912810 | 1 | 171312216 | g | 0.3296 | 0.0775 | 0.0098 | 3.51E-15 | 62.54 |
| Methylglutarylcarnitine | Acylcarnitines | rs1047891 | 2 | 211540507 | a | 0.319 | 0.2078 | 0.01 | 8.60E-96 | 431.81 |
| Methylglutarylcarnitine | Acylcarnitines | rs12208357 | 6 | 160543148 | t | 0.0775 | 0.1918 | 0.0175 | 5.98E-28 | 120.12 |
| Methylglutarylcarnitine | Acylcarnitines | rs1658973 | 9 | 6665097 | a | 0.8623 | 0.2074 | 0.0135 | 2.73E-53 | 236.02 |
| Methylglutarylcarnitine | Acylcarnitines | rs12492717 | 3 | 67418250 | g | 0.1091 | 0.1216 | 0.015 | 5.79E-16 | 65.72 |
| Methylglutarylcarnitine | Acylcarnitines | rs2163015 | 2 | 216908923 | c | 0.8994 | 0.1178 | 0.0156 | 3.78E-14 | 57.02 |
| Methylglutarylcarnitine | Acylcarnitines | rs1799822 | 1 | 53679229 | a | 0.7784 | 0.083 | 0.0113 | 2.07E-13 | 53.95 |
| Nonaylcarnitine | Acylcarnitines | rs2286963 | 2 | 211060050 | g | 0.3517 | 0.2572 | 0.0155 | 4.62E-62 | 275.35 |
| Nonaylcarnitine | Acylcarnitines | rs16833668 | 1 | 151918164 | a | 0.314 | 0.1043 | 0.0158 | 4.12E-11 | 43.58 |
| Octadecandienylcarnitine | Acylcarnitines | rs1171614 | 10 | 61469538 | c | 0.765 | 0.0867 | 0.0109 | 2.00E-15 | 63.27 |
| Octadecandienylcarnitine | Acylcarnitines | rs12210538 | 6 | 110760008 | a | 0.7598 | 0.256 | 0.0106 | 3.67E-128 | 583.27 |
| Octadecandienylcarnitine | Acylcarnitines | rs270601 | 5 | 131656997 | t | 0.2958 | 0.115 | 0.01 | 6.57E-31 | 132.25 |
| Octadecandienylcarnitine | Acylcarnitines | rs12764652 | 10 | 45991978 | a | 0.0643 | 0.1367 | 0.0191 | 7.65E-13 | 51.22 |
| Octadecanoylcarnitine | Acylcarnitines | rs1171617 | 10 | 61467182 | t | 0.7645 | 0.0943 | 0.0108 | 1.98E-18 | 76.24 |
| Octadecanoylcarnitine | Acylcarnitines | rs72939920 | 6 | 110762453 | a | 0.7604 | 0.179 | 0.0106 | 8.59E-64 | 285.16 |
| Octadecanoylcarnitine | Acylcarnitines | rs270613 | 5 | 131640583 | a | 0.381 | 0.0987 | 0.0094 | 9.02E-26 | 110.25 |
| Octadecanoylcarnitine | Acylcarnitines | rs61781290 | 1 | 40393160 | g | 0.2649 | 0.0652 | 0.0103 | 2.77E-10 | 40.07 |
| Octadecanoylcarnitine | Acylcarnitines | rs603424 | 10 | 102075479 | a | 0.1825 | 0.1038 | 0.0118 | 1.72E-18 | 77.38 |
| Octadecanoylcarnitine | Acylcarnitines | rs10103229 | 8 | 87248954 | c | 0.2314 | 0.0723 | 0.0107 | 1.40E-11 | 45.66 |
| Octadecenoylcarnitine | Acylcarnitines | rs12210538 | 6 | 110760008 | a | 0.7605 | 0.1854 | 0.017 | 8.57E-28 | 118.94 |
| Octadecenoylcarnitine | Acylcarnitines | rs270601 | 5 | 131656997 | t | 0.301 | 0.1134 | 0.0159 | 1.14E-12 | 50.87 |
| Octanoylcarnitine | Acylcarnitines | rs1171617 | 10 | 61467182 | t | 0.7642 | 0.0713 | 0.0109 | 6.41E-11 | 42.79 |
| Octanoylcarnitine | Acylcarnitines | rs7552404 | 1 | 76135946 | a | 0.692 | 0.2786 | 0.0099 | 6.95E-174 | 791.94 |
| Octanoylcarnitine | Acylcarnitines | rs8396 | 4 | 159630817 | t | 0.6992 | 0.1879 | 0.01 | 2.30E-79 | 353.06 |
| Octanoylcarnitine | Acylcarnitines | rs2062541 | 16 | 16127235 | a | 0.6097 | 0.0833 | 0.0095 | 1.32E-18 | 76.89 |
| Octanoylcarnitine | Acylcarnitines | rs77010315 | 5 | 150723155 | c | 0.9893 | 0.3164 | 0.0456 | 3.97E-12 | 48.14 |
| Ornithine | Amino acids | rs3961283 | 16 | 68317636 | a | 0.7267 | 0.0775 | 0.0101 | 1.75E-14 | 58.88 |
| Ornithine | Amino acids | rs17788484 | 6 | 131894358 | t | 0.0205 | 0.277 | 0.0327 | 2.51E-17 | 71.76 |
| Ornithine | Amino acids | rs4587804 | 12 | 47217514 | g | 0.3848 | 0.066 | 0.0095 | 3.04E-12 | 48.27 |
| Ornithine | Amino acids | rs687289 | 9 | 136137106 | g | 0.6747 | 0.0624 | 0.0095 | 5.84E-11 | 43.14 |
| Ornithine | Amino acids | rs77440950 | 19 | 13026412 | t | 0.8588 | 0.1061 | 0.0132 | 8.36E-16 | 64.61 |
| Ornithine | Amino acids | rs10024263 | 4 | 144959327 | c | 0.6877 | 0.0633 | 0.0097 | 6.55E-11 | 42.59 |
| Ornithine | Amino acids | rs921968 | 2 | 219272294 | t | 0.5924 | 0.0594 | 0.0091 | 8.12E-11 | 42.61 |
| Phenylalanine | Amino acids | rs869916 | 12 | 103244013 | g | 0.3904 | 0.0783 | 0.0056 | 3.53E-44 | 195.50 |
| Phenylalanine | Amino acids | rs4253238 | 4 | 187148387 | t | 0.5107 | 0.0807 | 0.0054 | 1.17E-49 | 223.34 |
| Phenylalanine | Amino acids | rs1065853 | 19 | 45413233 | t | 0.0818 | 0.0636 | 0.01 | 2.44E-10 | 40.45 |
| Phenylalanine | Amino acids | rs2731672 | 5 | 176842474 | c | 0.7497 | 0.0614 | 0.0064 | 1.07E-21 | 92.04 |
| Phenylalanine | Amino acids | rs710446 | 3 | 186459927 | c | 0.4082 | 0.0468 | 0.0056 | 5.86E-17 | 69.84 |
| Phenylalanine | Amino acids | rs1800787 | 4 | 155484015 | t | 0.1965 | 0.05 | 0.0068 | 2.82E-13 | 54.07 |
| Phenylalanine | Amino acids | rs2649667 | 11 | 57270509 | g | 0.7457 | 0.0388 | 0.0063 | 6.05E-10 | 37.93 |
| Pimelylcarnitine | Acylcarnitines | rs12921623 | 16 | 16096133 | c | 0.6133 | 0.0948 | 0.015 | 2.54E-10 | 39.94 |
| Proline | Amino acids | rs3970551 | 22 | 18906839 | g | 0.1145 | 0.4796 | 0.0149 | 2.41E-226 | 1036.06 |
| Proline | Amino acids | rs17666239 | 12 | 47194757 | a | 0.0675 | 0.1129 | 0.0177 | 1.79E-10 | 40.69 |
| Proline | Amino acids | rs10882649 | 10 | 97426086 | g | 0.342 | 0.0544 | 0.0093 | 5.93E-09 | 34.22 |
| Propionylcarnitine | Acylcarnitines | rs1171615 | 10 | 61469090 | t | 0.7649 | 0.2738 | 0.0105 | 7.02E-149 | 679.97 |
| Propionylcarnitine | Acylcarnitines | rs662138 | 6 | 160564476 | c | 0.8164 | 0.1784 | 0.0114 | 9.82E-55 | 244.90 |
| Propionylcarnitine | Acylcarnitines | rs270605 | 5 | 131651811 | c | 0.3793 | 0.1294 | 0.0091 | 1.58E-45 | 202.20 |
| Propionylcarnitine | Acylcarnitines | rs77010315 | 5 | 150723155 | c | 0.9893 | 0.3536 | 0.0441 | 1.06E-15 | 64.29 |
| Propionylcarnitine | Acylcarnitines | rs61749895 | 13 | 101020733 | t | 0.0108 | 0.4687 | 0.0436 | 5.66E-27 | 115.56 |
| Propionylcarnitine | Acylcarnitines | rs9382940 | 6 | 14133573 | t | 0.1815 | 0.0875 | 0.0117 | 5.87E-14 | 55.93 |
| Sarcosine | Biogenic amines | rs111389997 | 9 | 136601769 | c | 0.8368 | 0.1542 | 0.0126 | 2.23E-34 | 149.77 |
| Sarcosine | Biogenic amines | rs6940814 | 6 | 42924932 | a | 0.4179 | 0.0593 | 0.0095 | 3.89E-10 | 38.96 |
| Serine | Amino acids | rs715 | 2 | 211543055 | c | 0.3124 | 0.1671 | 0.0099 | 3.32E-64 | 284.89 |
| Serine | Amino acids | rs477992 | 1 | 120257576 | g | 0.6821 | 0.1837 | 0.0097 | 2.01E-80 | 358.65 |
| Serine | Amino acids | rs4947534 | 7 | 56079094 | c | 0.749 | 0.1615 | 0.0105 | 1.01E-53 | 236.57 |
| Serine | Amino acids | rs1260326 | 2 | 27730940 | c | 0.5993 | 0.0851 | 0.0092 | 2.48E-20 | 85.56 |
| Serine | Amino acids | rs28601761 | 8 | 126500031 | g | 0.4135 | 0.0745 | 0.0095 | 4.17E-15 | 61.50 |
| Spermidine | Biogenic amines | rs3811444 | 1 | 248039451 | t | 0.3484 | 0.1828 | 0.0113 | 5.43E-59 | 261.70 |
| Spermidine | Biogenic amines | rs62143194 | 19 | 54319624 | g | 0.2331 | 0.1573 | 0.0134 | 5.63E-32 | 137.80 |
| Symmetric dimethylarginine | Biogenic amines | rs37369 | 5 | 35037115 | t | 0.0915 | 0.2078 | 0.0255 | 3.27E-16 | 66.41 |
| Taurine | Biogenic amines | rs4632248 | 19 | 54324995 | t | 0.2148 | 0.1711 | 0.0112 | 1.14E-52 | 233.38 |
| Tetradecadienylcarnitine | Acylcarnitines | rs924135 | 16 | 16123459 | t | 0.6056 | 0.0832 | 0.0149 | 2.34E-08 | 31.18 |
| Tetradecanoylcarnitine | Acylcarnitines | rs1171617 | 10 | 61467182 | t | 0.7642 | 0.0768 | 0.0109 | 1.48E-12 | 49.64 |
| Tetradecanoylcarnitine | Acylcarnitines | rs12210538 | 6 | 110760008 | a | 0.7602 | 0.1097 | 0.0107 | 1.44E-24 | 105.11 |
| Tetradecanoylcarnitine | Acylcarnitines | rs199537349 | 5 | 131664529 | t | 0.5411 | 0.1117 | 0.0104 | 1.07E-26 | 115.36 |
| Tetradecenoylcarnitine | Acylcarnitines | rs8396 | 4 | 159630817 | t | 0.6993 | 0.0692 | 0.01 | 4.30E-12 | 47.89 |
| Tetradecenoylcarnitine | Acylcarnitines | rs156322 | 5 | 131653925 | c | 0.2962 | 0.0704 | 0.01 | 2.26E-12 | 49.56 |
| Tetradecenoylcarnitine | Acylcarnitines | rs12921623 | 16 | 16096133 | c | 0.6162 | 0.0766 | 0.0096 | 1.24E-15 | 63.67 |
| Threonine | Amino acids | rs715 | 2 | 211543055 | t | 0.6877 | 0.0732 | 0.01 | 2.45E-13 | 53.58 |
| Threonine | Amino acids | rs2657879 | 12 | 56865338 | g | 0.1847 | 0.0917 | 0.0118 | 6.96E-15 | 60.39 |
| Threonine | Amino acids | rs1260326 | 2 | 27730940 | c | 0.5993 | 0.1229 | 0.0093 | 8.01E-40 | 174.64 |
| Threonine | Amino acids | rs2465216 | 12 | 47240162 | c | 0.3457 | 0.0768 | 0.0097 | 2.42E-15 | 62.69 |
| Threonine | Amino acids | rs72661853 | 8 | 74876293 | t | 0.2957 | 0.1039 | 0.0101 | 6.46E-25 | 105.83 |
| Threonine | Amino acids | rs74095612 | 12 | 59970332 | t | 0.1109 | 0.0923 | 0.0146 | 2.45E-10 | 39.97 |
| Threonine | Amino acids | rs1742425 | 16 | 1904524 | g | 0.7942 | 0.0839 | 0.0114 | 1.92E-13 | 54.16 |
| trans-Hydroxyproline | Biogenic amines | rs3761097 | 19 | 36290977 | t | 0.0526 | 0.1806 | 0.0219 | 1.60E-16 | 68.01 |
| Tryptophan | Amino acids | rs17756732 | 6 | 111580154 | c | 0.1468 | 0.1178 | 0.0126 | 1.14E-20 | 87.41 |
| Tryptophan | Amino acids | rs17314234 | 4 | 156810755 | c | 0.9002 | 0.1723 | 0.015 | 2.03E-30 | 131.94 |
| Tyrosine | Amino acids | rs2393775 | 12 | 121424574 | g | 0.3819 | 0.0375 | 0.0058 | 1.42E-10 | 41.80 |
| Tyrosine | Amino acids | rs2638314 | 12 | 56866334 | a | 0.1829 | 0.06 | 0.0073 | 2.84E-16 | 67.55 |
| Tyrosine | Amino acids | rs241768 | 6 | 111492119 | t | 0.2804 | 0.1247 | 0.0064 | 1.71E-85 | 379.64 |
| Tyrosine | Amino acids | rs117866491 | 16 | 71628046 | t | 0.0175 | 0.3326 | 0.0235 | 1.52E-45 | 200.31 |
| Tyrosine | Amino acids | rs17666239 | 12 | 47194757 | a | 0.0683 | 0.0794 | 0.0112 | 1.65E-12 | 50.26 |
| Tyrosine | Amino acids | rs1935 | 10 | 64927823 | g | 0.4773 | 0.0337 | 0.0057 | 2.86E-09 | 34.96 |
| Valerylcarnitine | Acylcarnitines | rs2014355 | 12 | 121175524 | c | 0.2546 | 0.1016 | 0.0154 | 4.55E-11 | 43.53 |
| Valerylcarnitine | Acylcarnitines | rs1171615 | 10 | 61469090 | t | 0.7636 | 0.1366 | 0.016 | 1.67E-17 | 72.89 |
| Valerylcarnitine | Acylcarnitines | rs274556 | 5 | 131722075 | g | 0.3005 | 0.1877 | 0.0148 | 5.63E-37 | 160.84 |
| Valerylcarnitine | Acylcarnitines | rs8033249 | 15 | 40721939 | a | 0.5888 | 0.0985 | 0.0137 | 5.83E-13 | 51.69 |
| Valine | Amino acids | rs7678928 | 4 | 89222827 | t | 0.4654 | 0.0802 | 0.0056 | 7.33E-46 | 205.10 |
| Valine | Amino acids | rs2422358 | 2 | 65231806 | t | 0.4462 | 0.062 | 0.0059 | 1.19E-25 | 110.43 |
| Valine | Amino acids | rs12149660 | 16 | 70309237 | g | 0.8873 | 0.0704 | 0.0089 | 2.29E-15 | 62.57 |
| Valine | Amino acids | rs964184 | 11 | 116648917 | c | 0.8674 | 0.0736 | 0.0082 | 2.57E-19 | 80.56 |
| Valine | Amino acids | rs4801776 | 19 | 49304215 | c | 0.7293 | 0.0561 | 0.0064 | 1.86E-18 | 76.84 |
| Valine | Amino acids | rs117643180 | 17 | 7185779 | c | 0.9738 | 0.1229 | 0.0179 | 6.01E-12 | 47.14 |

| **Supplementary Table 3.** The association between exposure and overall ovarian cancer in the MR analysis. | | | | | | | | | | | |
| --- | --- | --- | --- | --- | --- | --- | --- | --- | --- | --- | --- |
| Outcome | Exposure | Method | No. | beta | se | OR | LCI | UCI | *P*-value | *P* for heterogenity | *P* for pleiotropy |
| Overall ovarian cancer | Acetylcarnitine | Inverse variance weighted (fixed effects) | 2 | -0.07 | 0.10 | 0.93 | 0.77 | 1.14 | 0.496 | 0.794 |  |
| Overall ovarian cancer | Acetylcarnitine | Maximum likelihood | 2 | -0.07 | 0.10 | 0.93 | 0.77 | 1.14 | 0.496 |  |  |
| Overall ovarian cancer | Acetylornithine | Inverse variance weighted (fixed effects) | 2 | 0.01 | 0.02 | 1.01 | 0.97 | 1.05 | 0.676 | 0.074 |  |
| Overall ovarian cancer | Acetylornithine | Maximum likelihood | 2 | 0.01 | 0.02 | 1.01 | 0.97 | 1.05 | 0.675 |  |  |
| Overall ovarian cancer | Alanine | Inverse variance weighted (fixed effects) | 8 | -0.05 | 0.11 | 0.95 | 0.76 | 1.19 | 0.653 | 0.950 |  |
| Overall ovarian cancer | Alanine | Maximum likelihood | 8 | -0.05 | 0.11 | 0.95 | 0.76 | 1.19 | 0.651 |  |  |
| Overall ovarian cancer | Alanine | Simple median | 8 | -0.02 | 0.14 | 0.98 | 0.74 | 1.29 | 0.869 |  |  |
| Overall ovarian cancer | Alanine | Weighted median | 8 | -0.07 | 0.14 | 0.93 | 0.71 | 1.23 | 0.621 |  |  |
| Overall ovarian cancer | Alanine | MR Egger | 8 | -0.32 | 0.46 | 0.73 | 0.29 | 1.80 | 0.517 |  | 0.573 |
| Overall ovarian cancer | alpha-Aminoadipic acid | Inverse variance weighted (fixed effects) | 2 | 0.02 | 0.12 | 1.02 | 0.81 | 1.28 | 0.891 | 0.495 |  |
| Overall ovarian cancer | alpha-Aminoadipic acid | Maximum likelihood | 2 | 0.02 | 0.12 | 1.02 | 0.81 | 1.28 | 0.890 |  |  |
| Overall ovarian cancer | Arginine | Inverse variance weighted (fixed effects) | 7 | -0.09 | 0.06 | 0.92 | 0.82 | 1.03 | 0.137 | 0.438 |  |
| Overall ovarian cancer | Arginine | Maximum likelihood | 7 | -0.09 | 0.06 | 0.92 | 0.81 | 1.03 | 0.136 |  |  |
| Overall ovarian cancer | Arginine | Simple median | 7 | -0.07 | 0.08 | 0.93 | 0.79 | 1.09 | 0.380 |  |  |
| Overall ovarian cancer | Arginine | Weighted median | 7 | -0.12 | 0.08 | 0.89 | 0.76 | 1.03 | 0.113 |  |  |
| Overall ovarian cancer | Arginine | MR Egger | 7 | -0.13 | 0.11 | 0.88 | 0.71 | 1.08 | 0.268 |  | 0.632 |
| Overall ovarian cancer | Asparagine | Inverse variance weighted (fixed effects) | 4 | 0.03 | 0.04 | 1.03 | 0.95 | 1.12 | 0.418 | 0.782 |  |
| Overall ovarian cancer | Asparagine | Maximum likelihood | 4 | 0.03 | 0.04 | 1.03 | 0.95 | 1.12 | 0.418 |  |  |
| Overall ovarian cancer | Asparagine | Simple median | 4 | 0.06 | 0.08 | 1.06 | 0.91 | 1.25 | 0.446 |  |  |
| Overall ovarian cancer | Asparagine | Weighted median | 4 | 0.04 | 0.04 | 1.04 | 0.95 | 1.13 | 0.378 |  |  |
| Overall ovarian cancer | Asparagine | MR Egger | 4 | 0.04 | 0.06 | 1.04 | 0.93 | 1.16 | 0.591 |  | 0.948 |
| Overall ovarian cancer | Aspartate | Inverse variance weighted (fixed effects) | 2 | 0.05 | 0.08 | 1.05 | 0.90 | 1.23 | 0.502 | 0.471 |  |
| Overall ovarian cancer | Aspartate | Maximum likelihood | 2 | 0.05 | 0.08 | 1.05 | 0.90 | 1.23 | 0.502 |  |  |
| Overall ovarian cancer | Butyrylcarnitine | Wald ratio | 1 | -0.04 | 0.15 | 0.96 | 0.71 | 1.29 | 0.785 | NA | NA |
| Overall ovarian cancer | Carnitine | Inverse variance weighted (fixed effects) | 3 | -0.05 | 0.10 | 0.95 | 0.78 | 1.16 | 0.645 | 0.624 |  |
| Overall ovarian cancer | Carnitine | Maximum likelihood | 3 | -0.05 | 0.10 | 0.95 | 0.78 | 1.16 | 0.644 |  |  |
| Overall ovarian cancer | Carnitine | Simple median | 3 | -0.06 | 0.13 | 0.95 | 0.74 | 1.22 | 0.666 |  |  |
| Overall ovarian cancer | Carnitine | Weighted median | 3 | -0.02 | 0.10 | 0.98 | 0.80 | 1.20 | 0.827 |  |  |
| Overall ovarian cancer | Carnitine | MR Egger | 3 | -0.03 | 0.15 | 0.98 | 0.72 | 1.32 | 0.897 |  | 0.884 |
| Overall ovarian cancer | Citrulline | Inverse variance weighted (fixed effects) | 4 | 0.04 | 0.08 | 1.04 | 0.89 | 1.22 | 0.617 | 0.693 |  |
| Overall ovarian cancer | Citrulline | Maximum likelihood | 4 | 0.04 | 0.08 | 1.04 | 0.89 | 1.23 | 0.617 |  |  |
| Overall ovarian cancer | Citrulline | Simple median | 4 | 0.06 | 0.09 | 1.06 | 0.89 | 1.27 | 0.525 |  |  |
| Overall ovarian cancer | Citrulline | Weighted median | 4 | 0.06 | 0.09 | 1.07 | 0.89 | 1.28 | 0.500 |  |  |
| Overall ovarian cancer | Citrulline | MR Egger | 4 | -0.59 | 0.54 | 0.55 | 0.19 | 1.60 | 0.388 |  | 0.358 |
| Overall ovarian cancer | Creatinine | Inverse variance weighted (fixed effects) | 9 | 0.14 | 0.12 | 1.15 | 0.91 | 1.45 | 0.245 | 0.203 |  |
| Overall ovarian cancer | Creatinine | Maximum likelihood | 9 | 0.14 | 0.12 | 1.15 | 0.91 | 1.46 | 0.240 |  |  |
| Overall ovarian cancer | Creatinine | Simple median | 9 | 0.19 | 0.17 | 1.20 | 0.86 | 1.69 | 0.279 |  |  |
| Overall ovarian cancer | Creatinine | Weighted median | 9 | 0.20 | 0.15 | 1.22 | 0.91 | 1.63 | 0.180 |  |  |
| Overall ovarian cancer | Creatinine | MR Egger | 9 | 0.35 | 0.67 | 1.41 | 0.38 | 5.21 | 0.620 |  | 0.758 |
| Overall ovarian cancer | Decanoylcarnitine | Inverse variance weighted (multiplicative random effects) | 2 | 0.00 | 0.11 | 1.00 | 0.80 | 1.25 | 0.997 | 0.027 |  |
| Overall ovarian cancer | Decanoylcarnitine | Maximum likelihood | 2 | 0.00 | 0.05 | 1.00 | 0.90 | 1.11 | 0.993 |  |  |
| Overall ovarian cancer | Decenoylcarnitine | Inverse variance weighted (fixed effects) | 3 | 0.01 | 0.06 | 1.01 | 0.89 | 1.14 | 0.886 | 0.085 |  |
| Overall ovarian cancer | Decenoylcarnitine | Maximum likelihood | 3 | 0.01 | 0.06 | 1.01 | 0.89 | 1.14 | 0.885 |  |  |
| Overall ovarian cancer | Decenoylcarnitine | Simple median | 3 | -0.04 | 0.11 | 0.96 | 0.78 | 1.20 | 0.735 |  |  |
| Overall ovarian cancer | Decenoylcarnitine | Weighted median | 3 | 0.03 | 0.07 | 1.03 | 0.89 | 1.19 | 0.693 |  |  |
| Overall ovarian cancer | Decenoylcarnitine | MR Egger | 3 | 0.31 | 0.39 | 1.37 | 0.63 | 2.95 | 0.571 |  | 0.568 |
| Overall ovarian cancer | Dodecanoylcarnitine | Wald ratio | 1 | -0.23 | 0.25 | 0.80 | 0.49 | 1.29 | 0.358 | NA | NA |
| Overall ovarian cancer | Glutamine | Inverse variance weighted (fixed effects) | 5 | 0.11 | 0.12 | 1.12 | 0.89 | 1.40 | 0.349 | 0.796 |  |
| Overall ovarian cancer | Glutamine | Maximum likelihood | 5 | 0.11 | 0.12 | 1.12 | 0.89 | 1.40 | 0.349 |  |  |
| Overall ovarian cancer | Glutamine | Simple median | 5 | 0.12 | 0.15 | 1.13 | 0.84 | 1.52 | 0.430 |  |  |
| Overall ovarian cancer | Glutamine | Weighted median | 5 | 0.11 | 0.14 | 1.12 | 0.85 | 1.47 | 0.430 |  |  |
| Overall ovarian cancer | Glutamine | MR Egger | 5 | -0.26 | 0.50 | 0.77 | 0.29 | 2.06 | 0.639 |  | 0.504 |
| Overall ovarian cancer | Glycine | Inverse variance weighted (multiplicative random effects) | 10 | -0.02 | 0.05 | 0.98 | 0.90 | 1.07 | 0.651 | 0.018 |  |
| Overall ovarian cancer | Glycine | Maximum likelihood | 10 | -0.02 | 0.03 | 0.98 | 0.92 | 1.04 | 0.496 |  |  |
| Overall ovarian cancer | Glycine | Simple median | 10 | -0.14 | 0.13 | 0.87 | 0.68 | 1.12 | 0.285 |  |  |
| Overall ovarian cancer | Glycine | Weighted median | 10 | 0.01 | 0.03 | 1.01 | 0.95 | 1.08 | 0.773 |  |  |
| Overall ovarian cancer | Glycine | MR Egger | 10 | 0.02 | 0.05 | 1.02 | 0.92 | 1.14 | 0.727 |  | 0.242 |
| Overall ovarian cancer | Hexadecanoylcarnitine | Wald ratio | 1 | -0.10 | 0.12 | 0.90 | 0.72 | 1.13 | 0.379 | NA | NA |
| Overall ovarian cancer | Hexadecenoylcarnitine | Wald ratio | 1 | -0.39 | 0.15 | 0.68 | 0.51 | 0.91 | 0.010 | NA | NA |
| Overall ovarian cancer | Hexanoylcarnitine | Inverse variance weighted (fixed effects) | 2 | -0.13 | 0.07 | 0.88 | 0.76 | 1.02 | 0.087 | 0.745 |  |
| Overall ovarian cancer | Hexanoylcarnitine | Maximum likelihood | 2 | -0.13 | 0.07 | 0.88 | 0.76 | 1.02 | 0.090 |  |  |
| Overall ovarian cancer | Hexose | Wald ratio | 1 | 0.09 | 0.15 | 1.10 | 0.82 | 1.47 | 0.525 | NA | NA |
| Overall ovarian cancer | Histidine | Inverse variance weighted (multiplicative random effects) | 7 | -0.07 | 0.24 | 0.93 | 0.58 | 1.50 | 0.760 | 0.000 |  |
| Overall ovarian cancer | Histidine | Maximum likelihood | 7 | -0.08 | 0.10 | 0.92 | 0.76 | 1.12 | 0.423 |  |  |
| Overall ovarian cancer | Histidine | Simple median | 7 | -0.01 | 0.15 | 0.99 | 0.74 | 1.31 | 0.918 |  |  |
| Overall ovarian cancer | Histidine | Weighted median | 7 | 0.07 | 0.12 | 1.08 | 0.85 | 1.37 | 0.540 |  |  |
| Overall ovarian cancer | Histidine | MR Egger | 7 | 1.19 | 1.36 | 3.28 | 0.23 | 46.72 | 0.421 |  | 0.388 |
| Overall ovarian cancer | Isoleucine | Inverse variance weighted (fixed effects) | 2 | 0.02 | 0.26 | 1.02 | 0.61 | 1.69 | 0.943 | 0.405 |  |
| Overall ovarian cancer | Isoleucine | Maximum likelihood | 2 | 0.02 | 0.26 | 1.02 | 0.61 | 1.69 | 0.943 |  |  |
| Overall ovarian cancer | Kynurenine | Inverse variance weighted (fixed effects) | 2 | 0.03 | 0.07 | 1.03 | 0.90 | 1.17 | 0.689 | 0.526 |  |
| Overall ovarian cancer | Kynurenine | Maximum likelihood | 2 | 0.03 | 0.07 | 1.03 | 0.90 | 1.17 | 0.689 |  |  |
| Overall ovarian cancer | Leucine | Inverse variance weighted (fixed effects) | 4 | 0.26 | 0.18 | 1.30 | 0.92 | 1.84 | 0.135 | 0.462 |  |
| Overall ovarian cancer | Leucine | Maximum likelihood | 4 | 0.27 | 0.18 | 1.30 | 0.92 | 1.85 | 0.134 |  |  |
| Overall ovarian cancer | Leucine | Simple median | 4 | 0.25 | 0.20 | 1.28 | 0.86 | 1.91 | 0.222 |  |  |
| Overall ovarian cancer | Leucine | Weighted median | 4 | 0.26 | 0.21 | 1.30 | 0.85 | 1.98 | 0.225 |  |  |
| Overall ovarian cancer | Leucine | MR Egger | 4 | 0.27 | 0.64 | 1.31 | 0.37 | 4.63 | 0.712 |  | 0.988 |
| Overall ovarian cancer | Lysine | Inverse variance weighted (fixed effects) | 6 | -0.08 | 0.05 | 0.92 | 0.83 | 1.03 | 0.144 | 0.907 |  |
| Overall ovarian cancer | Lysine | Maximum likelihood | 6 | -0.08 | 0.05 | 0.92 | 0.83 | 1.03 | 0.144 |  |  |
| Overall ovarian cancer | Lysine | Simple median | 6 | -0.07 | 0.07 | 0.93 | 0.81 | 1.08 | 0.362 |  |  |
| Overall ovarian cancer | Lysine | Weighted median | 6 | -0.08 | 0.06 | 0.92 | 0.82 | 1.05 | 0.214 |  |  |
| Overall ovarian cancer | Lysine | MR Egger | 6 | -0.09 | 0.13 | 0.92 | 0.72 | 1.17 | 0.528 |  | 0.946 |
| Overall ovarian cancer | Methionine | Wald ratio | 1 | -0.34 | 0.23 | 0.71 | 0.45 | 1.11 | 0.130 | NA | NA |
| Overall ovarian cancer | Methioninesulfoxide | Wald ratio | 1 | -0.37 | 0.19 | 0.69 | 0.48 | 1.00 | 0.048 | NA | NA |
| Overall ovarian cancer | Methylglutarylcarnitine | Inverse variance weighted (fixed effects) | 4 | 0.00 | 0.06 | 1.00 | 0.90 | 1.12 | 0.959 | 0.576 |  |
| Overall ovarian cancer | Methylglutarylcarnitine | Maximum likelihood | 4 | 0.00 | 0.06 | 1.00 | 0.90 | 1.12 | 0.959 |  |  |
| Overall ovarian cancer | Methylglutarylcarnitine | Simple median | 4 | -0.06 | 0.08 | 0.95 | 0.80 | 1.11 | 0.503 |  |  |
| Overall ovarian cancer | Methylglutarylcarnitine | Weighted median | 4 | 0.02 | 0.06 | 1.02 | 0.90 | 1.15 | 0.745 |  |  |
| Overall ovarian cancer | Methylglutarylcarnitine | MR Egger | 4 | 0.09 | 0.16 | 1.10 | 0.80 | 1.51 | 0.617 |  | 0.607 |
| Overall ovarian cancer | Nonaylcarnitine | Inverse variance weighted (fixed effects) | 2 | 0.08 | 0.05 | 1.08 | 0.98 | 1.20 | 0.118 | 0.403 |  |
| Overall ovarian cancer | Nonaylcarnitine | Maximum likelihood | 2 | 0.08 | 0.05 | 1.08 | 0.98 | 1.20 | 0.119 |  |  |
| Overall ovarian cancer | Octadecandienylcarnitine | Inverse variance weighted (fixed effects) | 4 | -0.13 | 0.05 | 0.88 | 0.80 | 0.97 | 0.011 | 0.426 |  |
| Overall ovarian cancer | Octadecandienylcarnitine | Maximum likelihood | 4 | -0.13 | 0.05 | 0.88 | 0.79 | 0.97 | 0.011 |  |  |
| Overall ovarian cancer | Octadecandienylcarnitine | Simple median | 4 | -0.13 | 0.07 | 0.88 | 0.76 | 1.01 | 0.075 |  |  |
| Overall ovarian cancer | Octadecandienylcarnitine | Weighted median | 4 | -0.15 | 0.06 | 0.86 | 0.77 | 0.96 | 0.007 |  |  |
| Overall ovarian cancer | Octadecandienylcarnitine | MR Egger | 4 | -0.17 | 0.14 | 0.84 | 0.64 | 1.11 | 0.341 |  | 0.766 |
| Overall ovarian cancer | Octadecanoylcarnitine | Inverse variance weighted (fixed effects) | 4 | 0.02 | 0.09 | 1.02 | 0.86 | 1.21 | 0.859 | 0.551 |  |
| Overall ovarian cancer | Octadecanoylcarnitine | Maximum likelihood | 4 | 0.02 | 0.09 | 1.02 | 0.86 | 1.21 | 0.858 |  |  |
| Overall ovarian cancer | Octadecanoylcarnitine | Simple median | 4 | 0.06 | 0.11 | 1.07 | 0.86 | 1.32 | 0.560 |  |  |
| Overall ovarian cancer | Octadecanoylcarnitine | Weighted median | 4 | 0.00 | 0.11 | 1.00 | 0.81 | 1.25 | 0.968 |  |  |
| Overall ovarian cancer | Octadecanoylcarnitine | MR Egger | 4 | -0.47 | 0.46 | 0.63 | 0.25 | 1.55 | 0.419 |  | 0.400 |
| Overall ovarian cancer | Octadecenoylcarnitine | Inverse variance weighted (fixed effects) | 2 | -0.18 | 0.07 | 0.83 | 0.73 | 0.95 | 0.008 | 0.478 |  |
| Overall ovarian cancer | Octadecenoylcarnitine | Maximum likelihood | 2 | -0.18 | 0.07 | 0.83 | 0.72 | 0.96 | 0.010 |  |  |
| Overall ovarian cancer | Octanoylcarnitine | Inverse variance weighted (fixed effects) | 4 | 0.01 | 0.04 | 1.01 | 0.93 | 1.09 | 0.832 | 0.175 |  |
| Overall ovarian cancer | Octanoylcarnitine | Maximum likelihood | 4 | 0.01 | 0.04 | 1.01 | 0.93 | 1.09 | 0.831 |  |  |
| Overall ovarian cancer | Octanoylcarnitine | Simple median | 4 | -0.02 | 0.08 | 0.98 | 0.84 | 1.15 | 0.822 |  |  |
| Overall ovarian cancer | Octanoylcarnitine | Weighted median | 4 | 0.04 | 0.05 | 1.04 | 0.94 | 1.15 | 0.417 |  |  |
| Overall ovarian cancer | Octanoylcarnitine | MR Egger | 4 | 0.11 | 0.13 | 1.12 | 0.86 | 1.46 | 0.496 |  | 0.492 |
| Overall ovarian cancer | Ornithine | Inverse variance weighted (multiplicative random effects) | 5 | -0.17 | 0.25 | 0.85 | 0.52 | 1.39 | 0.508 | 0.000 |  |
| Overall ovarian cancer | Ornithine | Maximum likelihood | 5 | -0.19 | 0.10 | 0.83 | 0.68 | 1.00 | 0.051 |  |  |
| Overall ovarian cancer | Ornithine | Simple median | 5 | -0.24 | 0.16 | 0.79 | 0.58 | 1.07 | 0.130 |  |  |
| Overall ovarian cancer | Ornithine | Weighted median | 5 | -0.05 | 0.12 | 0.96 | 0.75 | 1.22 | 0.712 |  |  |
| Overall ovarian cancer | Ornithine | MR Egger | 5 | 0.45 | 0.57 | 1.56 | 0.51 | 4.78 | 0.492 |  | 0.322 |
| Overall ovarian cancer | Phenylalanine | Inverse variance weighted (fixed effects) | 4 | 0.26 | 0.12 | 1.29 | 1.03 | 1.62 | 0.028 | 0.894 |  |
| Overall ovarian cancer | Phenylalanine | Maximum likelihood | 4 | 0.26 | 0.12 | 1.29 | 1.03 | 1.63 | 0.029 |  |  |
| Overall ovarian cancer | Phenylalanine | Simple median | 4 | 0.28 | 0.14 | 1.32 | 1.00 | 1.74 | 0.050 |  |  |
| Overall ovarian cancer | Phenylalanine | Weighted median | 4 | 0.28 | 0.13 | 1.32 | 1.02 | 1.71 | 0.038 |  |  |
| Overall ovarian cancer | Phenylalanine | MR Egger | 4 | 0.19 | 0.52 | 1.21 | 0.44 | 3.32 | 0.748 |  | 0.907 |
| Overall ovarian cancer | Proline | Inverse variance weighted (fixed effects) | 2 | 0.09 | 0.05 | 1.10 | 0.99 | 1.21 | 0.063 | 0.850 |  |
| Overall ovarian cancer | Proline | Maximum likelihood | 2 | 0.09 | 0.05 | 1.10 | 0.99 | 1.21 | 0.064 |  |  |
| Overall ovarian cancer | Propionylcarnitine | Inverse variance weighted (fixed effects) | 4 | -0.07 | 0.05 | 0.94 | 0.85 | 1.03 | 0.166 | 0.967 |  |
| Overall ovarian cancer | Propionylcarnitine | Maximum likelihood | 4 | -0.07 | 0.05 | 0.94 | 0.85 | 1.03 | 0.166 |  |  |
| Overall ovarian cancer | Propionylcarnitine | Simple median | 4 | -0.07 | 0.07 | 0.94 | 0.81 | 1.08 | 0.364 |  |  |
| Overall ovarian cancer | Propionylcarnitine | Weighted median | 4 | -0.06 | 0.05 | 0.94 | 0.85 | 1.04 | 0.215 |  |  |
| Overall ovarian cancer | Propionylcarnitine | MR Egger | 4 | -0.03 | 0.11 | 0.97 | 0.77 | 1.21 | 0.795 |  | 0.774 |
| Overall ovarian cancer | Sarcosine | Inverse variance weighted (fixed effects) | 2 | -0.04 | 0.12 | 0.96 | 0.77 | 1.21 | 0.747 | 0.873 |  |
| Overall ovarian cancer | Sarcosine | Maximum likelihood | 2 | -0.04 | 0.12 | 0.96 | 0.77 | 1.21 | 0.747 |  |  |
| Overall ovarian cancer | Serine | Inverse variance weighted (fixed effects) | 3 | 0.03 | 0.05 | 1.03 | 0.93 | 1.15 | 0.565 | 0.896 |  |
| Overall ovarian cancer | Serine | Maximum likelihood | 3 | 0.03 | 0.05 | 1.03 | 0.93 | 1.15 | 0.566 |  |  |
| Overall ovarian cancer | Serine | Simple median | 3 | 0.04 | 0.07 | 1.04 | 0.91 | 1.18 | 0.601 |  |  |
| Overall ovarian cancer | Serine | Weighted median | 3 | 0.03 | 0.06 | 1.03 | 0.92 | 1.14 | 0.644 |  |  |
| Overall ovarian cancer | Serine | MR Egger | 3 | -0.05 | 0.19 | 0.95 | 0.66 | 1.37 | 0.838 |  | 0.732 |
| Overall ovarian cancer | Spermidine | Wald ratio | 1 | 0.00 | 0.08 | 1.00 | 0.86 | 1.17 | 0.971 | NA | NA |
| Overall ovarian cancer | Symmetric dimethylarginine | Wald ratio | 1 | 0.01 | 0.12 | 1.01 | 0.80 | 1.26 | 0.952 | NA | NA |
| Overall ovarian cancer | Tetradecanoylcarnitine | Inverse variance weighted (fixed effects) | 2 | -0.22 | 0.10 | 0.80 | 0.66 | 0.97 | 0.020 | 0.168 |  |
| Overall ovarian cancer | Tetradecanoylcarnitine | Maximum likelihood | 2 | -0.22 | 0.10 | 0.80 | 0.66 | 0.97 | 0.022 |  |  |
| Overall ovarian cancer | Tetradecenoylcarnitine | Inverse variance weighted (fixed effects) | 2 | -0.27 | 0.15 | 0.76 | 0.57 | 1.02 | 0.069 | 0.561 |  |
| Overall ovarian cancer | Tetradecenoylcarnitine | Maximum likelihood | 2 | -0.27 | 0.15 | 0.76 | 0.57 | 1.03 | 0.074 |  |  |
| Overall ovarian cancer | Threonine | Inverse variance weighted (fixed effects) | 3 | -0.05 | 0.09 | 0.96 | 0.81 | 1.13 | 0.599 | 0.162 |  |
| Overall ovarian cancer | Threonine | Maximum likelihood | 3 | -0.05 | 0.09 | 0.95 | 0.81 | 1.13 | 0.597 |  |  |
| Overall ovarian cancer | Threonine | Simple median | 3 | -0.08 | 0.14 | 0.92 | 0.70 | 1.22 | 0.570 |  |  |
| Overall ovarian cancer | Threonine | Weighted median | 3 | 0.00 | 0.10 | 1.00 | 0.82 | 1.21 | 0.978 |  |  |
| Overall ovarian cancer | Threonine | MR Egger | 3 | 0.45 | 0.51 | 1.57 | 0.57 | 4.30 | 0.542 |  | 0.503 |
| Overall ovarian cancer | Tryptophan | Inverse variance weighted (fixed effects) | 2 | 0.04 | 0.10 | 1.04 | 0.86 | 1.27 | 0.661 | 0.464 |  |
| Overall ovarian cancer | Tryptophan | Maximum likelihood | 2 | 0.04 | 0.10 | 1.04 | 0.86 | 1.27 | 0.661 |  |  |
| Overall ovarian cancer | Tyrosine | Wald ratio | 1 | -0.07 | 0.18 | 0.93 | 0.66 | 1.33 | 0.697 | NA | NA |
| Overall ovarian cancer | Valerylcarnitine | Inverse variance weighted (fixed effects) | 3 | -0.04 | 0.06 | 0.96 | 0.86 | 1.08 | 0.517 | 0.194 |  |
| Overall ovarian cancer | Valerylcarnitine | Maximum likelihood | 3 | -0.04 | 0.06 | 0.96 | 0.86 | 1.08 | 0.515 |  |  |
| Overall ovarian cancer | Valerylcarnitine | Simple median | 3 | -0.07 | 0.07 | 0.93 | 0.81 | 1.07 | 0.322 |  |  |
| Overall ovarian cancer | Valerylcarnitine | Weighted median | 3 | -0.07 | 0.07 | 0.93 | 0.82 | 1.05 | 0.252 |  |  |
| Overall ovarian cancer | Valerylcarnitine | MR Egger | 3 | -0.34 | 0.25 | 0.71 | 0.44 | 1.16 | 0.401 |  | 0.426 |
| Overall ovarian cancer | Valine | Inverse variance weighted (fixed effects) | 4 | 0.16 | 0.12 | 1.17 | 0.92 | 1.49 | 0.193 | 0.993 |  |
| Overall ovarian cancer | Valine | Maximum likelihood | 4 | 0.16 | 0.12 | 1.17 | 0.92 | 1.49 | 0.194 |  |  |
| Overall ovarian cancer | Valine | Simple median | 4 | 0.16 | 0.15 | 1.17 | 0.87 | 1.57 | 0.297 |  |  |
| Overall ovarian cancer | Valine | Weighted median | 4 | 0.17 | 0.13 | 1.19 | 0.91 | 1.54 | 0.198 |  |  |
| Overall ovarian cancer | Valine | MR Egger | 4 | 0.25 | 0.62 | 1.29 | 0.38 | 4.32 | 0.724 |  | 0.893 |

| **Supplementary Table 4.** The association between exposure and high grade serous ovarian cancer. | | | | | | | | | | | | |
| --- | --- | --- | --- | --- | --- | --- | --- | --- | --- | --- | --- | --- |
| Outcome | Exposure | Method | No. | | beta | se | OR | LCI | UCI | *P*-value | *P* for heterogenity | *P* for pleiotropy |
| High grade serous ovarian cancer | Acetylcarnitine | Inverse variance weighted (fixed effects) | 3 | -0.03 | | 0.06 | 0.97 | 0.86 | 1.09 | 0.591 | 0.714 |  |
| High grade serous ovarian cancer | Acetylcarnitine | Maximum likelihood | 3 | -0.03 | | 0.06 | 0.97 | 0.86 | 1.09 | 0.591 |  |  |
| High grade serous ovarian cancer | Acetylcarnitine | Simple median | 3 | -0.01 | | 0.09 | 0.99 | 0.83 | 1.19 | 0.926 |  |  |
| High grade serous ovarian cancer | Acetylcarnitine | Weighted median | 3 | -0.01 | | 0.06 | 0.99 | 0.87 | 1.12 | 0.896 |  |  |
| High grade serous ovarian cancer | Acetylcarnitine | MR Egger | 3 | 0.08 | | 0.15 | 1.08 | 0.81 | 1.46 | 0.690 |  | 0.566 |
| High grade serous ovarian cancer | Acetylornithine | Inverse variance weighted (fixed effects) | 2 | 0.02 | | 0.02 | 1.02 | 0.98 | 1.07 | 0.351 | 0.071 |  |
| High grade serous ovarian cancer | Acetylornithine | Maximum likelihood | 2 | 0.02 | | 0.02 | 1.02 | 0.98 | 1.07 | 0.350 |  |  |
| High grade serous ovarian cancer | Alanine | Inverse variance weighted (fixed effects) | 9 | -0.11 | | 0.13 | 0.90 | 0.69 | 1.15 | 0.394 | 0.659 |  |
| High grade serous ovarian cancer | Alanine | Maximum likelihood | 9 | -0.11 | | 0.13 | 0.90 | 0.69 | 1.16 | 0.397 |  |  |
| High grade serous ovarian cancer | Alanine | Simple median | 9 | 0.03 | | 0.18 | 1.03 | 0.72 | 1.45 | 0.888 |  |  |
| High grade serous ovarian cancer | Alanine | Weighted median | 9 | -0.01 | | 0.18 | 0.99 | 0.70 | 1.41 | 0.963 |  |  |
| High grade serous ovarian cancer | Alanine | MR Egger | 9 | -0.16 | | 0.51 | 0.85 | 0.31 | 2.34 | 0.766 |  | 0.926 |
| High grade serous ovarian cancer | alpha-Aminoadipic acid | Inverse variance weighted (fixed effects) | 2 | 0.01 | | 0.14 | 1.01 | 0.77 | 1.34 | 0.918 | 0.360 |  |
| High grade serous ovarian cancer | alpha-Aminoadipic acid | Maximum likelihood | 2 | 0.01 | | 0.14 | 1.01 | 0.77 | 1.34 | 0.918 |  |  |
| High grade serous ovarian cancer | Arginine | Inverse variance weighted (fixed effects) | 7 | -0.01 | | 0.07 | 0.99 | 0.86 | 1.13 | 0.851 | 0.312 |  |
| High grade serous ovarian cancer | Arginine | Maximum likelihood | 7 | -0.01 | | 0.07 | 0.99 | 0.86 | 1.13 | 0.849 |  |  |
| High grade serous ovarian cancer | Arginine | Simple median | 7 | 0.01 | | 0.10 | 1.01 | 0.83 | 1.23 | 0.919 |  |  |
| High grade serous ovarian cancer | Arginine | Weighted median | 7 | -0.02 | | 0.09 | 0.98 | 0.82 | 1.17 | 0.790 |  |  |
| High grade serous ovarian cancer | Arginine | MR Egger | 7 | 0.01 | | 0.14 | 1.01 | 0.77 | 1.34 | 0.930 |  | 0.827 |
| High grade serous ovarian cancer | Asparagine | Inverse variance weighted (fixed effects) | 4 | 0.05 | | 0.05 | 1.05 | 0.95 | 1.16 | 0.304 | 0.203 |  |
| High grade serous ovarian cancer | Asparagine | Maximum likelihood | 4 | 0.05 | | 0.05 | 1.05 | 0.95 | 1.16 | 0.304 |  |  |
| High grade serous ovarian cancer | Asparagine | Simple median | 4 | 0.04 | | 0.09 | 1.05 | 0.87 | 1.25 | 0.627 |  |  |
| High grade serous ovarian cancer | Asparagine | Weighted median | 4 | 0.05 | | 0.05 | 1.05 | 0.94 | 1.16 | 0.376 |  |  |
| High grade serous ovarian cancer | Asparagine | MR Egger | 4 | 0.03 | | 0.10 | 1.03 | 0.84 | 1.25 | 0.821 |  | 0.759 |
| High grade serous ovarian cancer | Aspartate | Inverse variance weighted (fixed effects) | 2 | 0.02 | | 0.09 | 1.02 | 0.85 | 1.23 | 0.794 | 0.818 |  |
| High grade serous ovarian cancer | Aspartate | Maximum likelihood | 2 | 0.02 | | 0.09 | 1.02 | 0.85 | 1.23 | 0.794 |  |  |
| High grade serous ovarian cancer | Butyrylcarnitine | Inverse variance weighted (fixed effects) | 2 | -0.01 | | 0.09 | 0.99 | 0.84 | 1.17 | 0.929 | 0.935 |  |
| High grade serous ovarian cancer | Butyrylcarnitine | Maximum likelihood | 2 | -0.01 | | 0.09 | 0.99 | 0.84 | 1.17 | 0.929 |  |  |
| High grade serous ovarian cancer | Carnitine | Inverse variance weighted (fixed effects) | 5 | -0.02 | | 0.04 | 0.98 | 0.91 | 1.06 | 0.678 | 0.740 |  |
| High grade serous ovarian cancer | Carnitine | Maximum likelihood | 5 | -0.02 | | 0.04 | 0.98 | 0.91 | 1.06 | 0.678 |  |  |
| High grade serous ovarian cancer | Carnitine | Simple median | 5 | -0.03 | | 0.08 | 0.97 | 0.83 | 1.14 | 0.733 |  |  |
| High grade serous ovarian cancer | Carnitine | Weighted median | 5 | -0.01 | | 0.04 | 0.99 | 0.92 | 1.08 | 0.858 |  |  |
| High grade serous ovarian cancer | Carnitine | MR Egger | 5 | 0.02 | | 0.07 | 1.02 | 0.89 | 1.17 | 0.803 |  | 0.593 |
| High grade serous ovarian cancer | Citrulline | Inverse variance weighted (fixed effects) | 4 | -0.06 | | 0.10 | 0.94 | 0.77 | 1.14 | 0.507 | 0.532 |  |
| High grade serous ovarian cancer | Citrulline | Maximum likelihood | 4 | -0.07 | | 0.10 | 0.94 | 0.77 | 1.14 | 0.506 |  |  |
| High grade serous ovarian cancer | Citrulline | Simple median | 4 | -0.07 | | 0.11 | 0.93 | 0.75 | 1.16 | 0.531 |  |  |
| High grade serous ovarian cancer | Citrulline | Weighted median | 4 | -0.07 | | 0.12 | 0.94 | 0.74 | 1.18 | 0.567 |  |  |
| High grade serous ovarian cancer | Citrulline | MR Egger | 4 | -0.85 | | 0.64 | 0.43 | 0.12 | 1.50 | 0.316 |  | 0.340 |
| High grade serous ovarian cancer | Creatinine | Inverse variance weighted (fixed effects) | 12 | 0.15 | | 0.12 | 1.17 | 0.91 | 1.49 | 0.220 | 0.424 |  |
| High grade serous ovarian cancer | Creatinine | Maximum likelihood | 12 | 0.16 | | 0.13 | 1.17 | 0.91 | 1.50 | 0.217 |  |  |
| High grade serous ovarian cancer | Creatinine | Simple median | 12 | -0.04 | | 0.18 | 0.96 | 0.67 | 1.38 | 0.823 |  |  |
| High grade serous ovarian cancer | Creatinine | Weighted median | 12 | 0.24 | | 0.17 | 1.27 | 0.91 | 1.78 | 0.164 |  |  |
| High grade serous ovarian cancer | Creatinine | MR Egger | 12 | 0.67 | | 0.60 | 1.95 | 0.60 | 6.29 | 0.289 |  | 0.398 |
| High grade serous ovarian cancer | Decanoylcarnitine | Inverse variance weighted (fixed effects) | 2 | -0.03 | | 0.06 | 0.97 | 0.86 | 1.09 | 0.590 | 0.102 |  |
| High grade serous ovarian cancer | Decanoylcarnitine | Maximum likelihood | 2 | -0.03 | | 0.06 | 0.97 | 0.86 | 1.09 | 0.590 |  |  |
| High grade serous ovarian cancer | Decenoylcarnitine | Inverse variance weighted (fixed effects) | 3 | -0.01 | | 0.07 | 0.99 | 0.86 | 1.15 | 0.922 | 0.196 |  |
| High grade serous ovarian cancer | Decenoylcarnitine | Maximum likelihood | 3 | -0.01 | | 0.07 | 0.99 | 0.86 | 1.15 | 0.921 |  |  |
| High grade serous ovarian cancer | Decenoylcarnitine | Simple median | 3 | 0.06 | | 0.11 | 1.07 | 0.86 | 1.32 | 0.546 |  |  |
| High grade serous ovarian cancer | Decenoylcarnitine | Weighted median | 3 | 0.03 | | 0.09 | 1.03 | 0.87 | 1.22 | 0.753 |  |  |
| High grade serous ovarian cancer | Decenoylcarnitine | MR Egger | 3 | 0.07 | | 0.48 | 1.08 | 0.42 | 2.75 | 0.903 |  | 0.890 |
| High grade serous ovarian cancer | Dodecanoylcarnitine | Wald ratio | 1 | -0.29 | | 0.29 | 0.75 | 0.42 | 1.33 | 0.326 | NA | NA |
| High grade serous ovarian cancer | Glutamine | Inverse variance weighted (fixed effects) | 5 | 0.17 | | 0.14 | 1.18 | 0.90 | 1.56 | 0.223 | 0.324 |  |
| High grade serous ovarian cancer | Glutamine | Maximum likelihood | 5 | 0.17 | | 0.14 | 1.19 | 0.90 | 1.56 | 0.221 |  |  |
| High grade serous ovarian cancer | Glutamine | Simple median | 5 | 0.25 | | 0.22 | 1.29 | 0.84 | 1.97 | 0.251 |  |  |
| High grade serous ovarian cancer | Glutamine | Weighted median | 5 | 0.27 | | 0.18 | 1.31 | 0.92 | 1.86 | 0.139 |  |  |
| High grade serous ovarian cancer | Glutamine | MR Egger | 5 | -0.23 | | 0.71 | 0.79 | 0.20 | 3.19 | 0.766 |  | 0.602 |
| High grade serous ovarian cancer | Glycine | Inverse variance weighted (fixed effects) | 12 | -0.01 | | 0.04 | 0.99 | 0.93 | 1.06 | 0.829 | 0.226 |  |
| High grade serous ovarian cancer | Glycine | Maximum likelihood | 12 | -0.01 | | 0.04 | 0.99 | 0.93 | 1.06 | 0.832 |  |  |
| High grade serous ovarian cancer | Glycine | Simple median | 12 | 0.05 | | 0.11 | 1.05 | 0.85 | 1.30 | 0.650 |  |  |
| High grade serous ovarian cancer | Glycine | Weighted median | 12 | 0.00 | | 0.04 | 1.00 | 0.93 | 1.08 | 0.916 |  |  |
| High grade serous ovarian cancer | Glycine | MR Egger | 12 | -0.01 | | 0.05 | 0.99 | 0.89 | 1.10 | 0.840 |  | 0.919 |
| High grade serous ovarian cancer | Hexadecanoylcarnitine | Inverse variance weighted (fixed effects) | 2 | -0.09 | | 0.11 | 0.91 | 0.73 | 1.14 | 0.411 | 0.667 |  |
| High grade serous ovarian cancer | Hexadecanoylcarnitine | Maximum likelihood | 2 | -0.09 | | 0.11 | 0.91 | 0.73 | 1.14 | 0.412 |  |  |
| High grade serous ovarian cancer | Hexadecenoylcarnitine | Wald ratio | 1 | -0.30 | | 0.18 | 0.74 | 0.52 | 1.05 | 0.092 | NA | NA |
| High grade serous ovarian cancer | Hexanoylcarnitine | Inverse variance weighted (fixed effects) | 3 | 0.02 | | 0.05 | 1.02 | 0.91 | 1.13 | 0.780 | 0.247 |  |
| High grade serous ovarian cancer | Hexanoylcarnitine | Maximum likelihood | 3 | 0.02 | | 0.05 | 1.02 | 0.91 | 1.13 | 0.779 |  |  |
| High grade serous ovarian cancer | Hexanoylcarnitine | Simple median | 3 | -0.01 | | 0.09 | 0.99 | 0.83 | 1.17 | 0.877 |  |  |
| High grade serous ovarian cancer | Hexanoylcarnitine | Weighted median | 3 | 0.04 | | 0.06 | 1.04 | 0.92 | 1.17 | 0.562 |  |  |
| High grade serous ovarian cancer | Hexanoylcarnitine | MR Egger | 3 | 0.10 | | 0.08 | 1.10 | 0.95 | 1.28 | 0.427 |  | 0.375 |
| High grade serous ovarian cancer | Hexose | Wald ratio | 1 | -0.11 | | 0.18 | 0.90 | 0.64 | 1.27 | 0.545 | NA | NA |
| High grade serous ovarian cancer | Histidine | Inverse variance weighted (multiplicative random effects) | 7 | -0.08 | | 0.33 | 0.92 | 0.48 | 1.76 | 0.801 | 0.000 |  |
| High grade serous ovarian cancer | Histidine | Maximum likelihood | 7 | -0.09 | | 0.12 | 0.91 | 0.72 | 1.16 | 0.450 |  |  |
| High grade serous ovarian cancer | Histidine | Simple median | 7 | -0.04 | | 0.17 | 0.96 | 0.69 | 1.34 | 0.831 |  |  |
| High grade serous ovarian cancer | Histidine | Weighted median | 7 | 0.14 | | 0.15 | 1.15 | 0.85 | 1.56 | 0.352 |  |  |
| High grade serous ovarian cancer | Histidine | MR Egger | 7 | 1.36 | | 1.88 | 3.89 | 0.10 | 155.88 | 0.503 |  | 0.472 |
| High grade serous ovarian cancer | Isoleucine | Inverse variance weighted (fixed effects) | 3 | 0.23 | | 0.23 | 1.26 | 0.80 | 1.99 | 0.323 | 0.619 |  |
| High grade serous ovarian cancer | Isoleucine | Maximum likelihood | 3 | 0.23 | | 0.23 | 1.26 | 0.80 | 2.00 | 0.323 |  |  |
| High grade serous ovarian cancer | Isoleucine | Simple median | 3 | 0.18 | | 0.31 | 1.20 | 0.65 | 2.22 | 0.559 |  |  |
| High grade serous ovarian cancer | Isoleucine | Weighted median | 3 | 0.22 | | 0.27 | 1.24 | 0.73 | 2.12 | 0.430 |  |  |
| High grade serous ovarian cancer | Isoleucine | MR Egger | 3 | -0.32 | | 1.05 | 0.72 | 0.09 | 5.70 | 0.810 |  | 0.685 |
| High grade serous ovarian cancer | Kynurenine | Inverse variance weighted (fixed effects) | 3 | -0.02 | | 0.07 | 0.98 | 0.85 | 1.13 | 0.777 | 0.844 |  |
| High grade serous ovarian cancer | Kynurenine | Maximum likelihood | 3 | -0.02 | | 0.07 | 0.98 | 0.85 | 1.13 | 0.777 |  |  |
| High grade serous ovarian cancer | Kynurenine | Simple median | 3 | -0.02 | | 0.10 | 0.98 | 0.80 | 1.19 | 0.826 |  |  |
| High grade serous ovarian cancer | Kynurenine | Weighted median | 3 | -0.04 | | 0.08 | 0.96 | 0.82 | 1.13 | 0.662 |  |  |
| High grade serous ovarian cancer | Kynurenine | MR Egger | 3 | -0.13 | | 0.22 | 0.88 | 0.57 | 1.34 | 0.657 |  | 0.688 |
| High grade serous ovarian cancer | Leucine | Inverse variance weighted (fixed effects) | 4 | 0.11 | | 0.21 | 1.12 | 0.74 | 1.68 | 0.596 | 0.083 |  |
| High grade serous ovarian cancer | Leucine | Maximum likelihood | 4 | 0.11 | | 0.21 | 1.12 | 0.74 | 1.70 | 0.591 |  |  |
| High grade serous ovarian cancer | Leucine | Simple median | 4 | 0.18 | | 0.25 | 1.19 | 0.73 | 1.95 | 0.480 |  |  |
| High grade serous ovarian cancer | Leucine | Weighted median | 4 | 0.24 | | 0.25 | 1.27 | 0.77 | 2.07 | 0.350 |  |  |
| High grade serous ovarian cancer | Leucine | MR Egger | 4 | 0.52 | | 1.19 | 1.69 | 0.16 | 17.41 | 0.703 |  | 0.750 |
| High grade serous ovarian cancer | Lysine | Inverse variance weighted (fixed effects) | 6 | -0.08 | | 0.06 | 0.93 | 0.82 | 1.05 | 0.234 | 0.715 |  |
| High grade serous ovarian cancer | Lysine | Maximum likelihood | 6 | -0.08 | | 0.06 | 0.93 | 0.82 | 1.05 | 0.233 |  |  |
| High grade serous ovarian cancer | Lysine | Simple median | 6 | -0.12 | | 0.09 | 0.89 | 0.75 | 1.06 | 0.183 |  |  |
| High grade serous ovarian cancer | Lysine | Weighted median | 6 | -0.12 | | 0.08 | 0.89 | 0.76 | 1.04 | 0.145 |  |  |
| High grade serous ovarian cancer | Lysine | MR Egger | 6 | -0.06 | | 0.15 | 0.94 | 0.71 | 1.26 | 0.722 |  | 0.895 |
| High grade serous ovarian cancer | Methionine | Wald ratio | 1 | -0.49 | | 0.27 | 0.61 | 0.36 | 1.04 | 0.069 | NA | NA |
| High grade serous ovarian cancer | Methioninesulfoxide | Wald ratio | 1 | -0.30 | | 0.22 | 0.74 | 0.48 | 1.15 | 0.179 | NA | NA |
| High grade serous ovarian cancer | Methylglutarylcarnitine | Inverse variance weighted (fixed effects) | 4 | 0.00 | | 0.07 | 1.00 | 0.88 | 1.13 | 0.948 | 0.708 |  |
| High grade serous ovarian cancer | Methylglutarylcarnitine | Maximum likelihood | 4 | 0.00 | | 0.07 | 1.00 | 0.88 | 1.13 | 0.948 |  |  |
| High grade serous ovarian cancer | Methylglutarylcarnitine | Simple median | 4 | -0.08 | | 0.10 | 0.93 | 0.77 | 1.12 | 0.428 |  |  |
| High grade serous ovarian cancer | Methylglutarylcarnitine | Weighted median | 4 | 0.00 | | 0.07 | 1.00 | 0.87 | 1.16 | 0.951 |  |  |
| High grade serous ovarian cancer | Methylglutarylcarnitine | MR Egger | 4 | 0.09 | | 0.19 | 1.10 | 0.75 | 1.60 | 0.673 |  | 0.641 |
| High grade serous ovarian cancer | Nonaylcarnitine | Inverse variance weighted (fixed effects) | 2 | 0.10 | | 0.06 | 1.10 | 0.98 | 1.24 | 0.113 | 0.786 |  |
| High grade serous ovarian cancer | Nonaylcarnitine | Maximum likelihood | 2 | 0.10 | | 0.06 | 1.10 | 0.98 | 1.24 | 0.114 |  |  |
| High grade serous ovarian cancer | Octadecandienylcarnitine | Inverse variance weighted (fixed effects) | 4 | -0.11 | | 0.06 | 0.90 | 0.80 | 1.01 | 0.073 | 0.922 |  |
| High grade serous ovarian cancer | Octadecandienylcarnitine | Maximum likelihood | 4 | -0.11 | | 0.06 | 0.90 | 0.80 | 1.01 | 0.074 |  |  |
| High grade serous ovarian cancer | Octadecandienylcarnitine | Simple median | 4 | -0.07 | | 0.09 | 0.93 | 0.77 | 1.11 | 0.417 |  |  |
| High grade serous ovarian cancer | Octadecandienylcarnitine | Weighted median | 4 | -0.12 | | 0.06 | 0.88 | 0.78 | 1.00 | 0.050 |  |  |
| High grade serous ovarian cancer | Octadecandienylcarnitine | MR Egger | 4 | -0.15 | | 0.14 | 0.86 | 0.65 | 1.14 | 0.401 |  | 0.766 |
| High grade serous ovarian cancer | Octadecanoylcarnitine | Inverse variance weighted (fixed effects) | 5 | -0.05 | | 0.09 | 0.95 | 0.79 | 1.14 | 0.591 | 0.439 |  |
| High grade serous ovarian cancer | Octadecanoylcarnitine | Maximum likelihood | 5 | -0.05 | | 0.09 | 0.95 | 0.79 | 1.14 | 0.590 |  |  |
| High grade serous ovarian cancer | Octadecanoylcarnitine | Simple median | 5 | -0.02 | | 0.13 | 0.98 | 0.75 | 1.26 | 0.857 |  |  |
| High grade serous ovarian cancer | Octadecanoylcarnitine | Weighted median | 5 | -0.12 | | 0.11 | 0.89 | 0.71 | 1.10 | 0.280 |  |  |
| High grade serous ovarian cancer | Octadecanoylcarnitine | MR Egger | 5 | -1.01 | | 0.54 | 0.36 | 0.13 | 1.04 | 0.155 |  | 0.166 |
| High grade serous ovarian cancer | Octadecenoylcarnitine | Inverse variance weighted (fixed effects) | 2 | -0.16 | | 0.08 | 0.85 | 0.72 | 1.00 | 0.056 | 0.863 |  |
| High grade serous ovarian cancer | Octadecenoylcarnitine | Maximum likelihood | 2 | -0.16 | | 0.08 | 0.85 | 0.72 | 1.01 | 0.059 |  |  |
| High grade serous ovarian cancer | Octanoylcarnitine | Inverse variance weighted (fixed effects) | 5 | -0.01 | | 0.05 | 0.99 | 0.90 | 1.09 | 0.802 | 0.564 |  |
| High grade serous ovarian cancer | Octanoylcarnitine | Maximum likelihood | 5 | -0.01 | | 0.05 | 0.99 | 0.90 | 1.09 | 0.802 |  |  |
| High grade serous ovarian cancer | Octanoylcarnitine | Simple median | 5 | 0.00 | | 0.09 | 1.00 | 0.83 | 1.21 | 0.963 |  |  |
| High grade serous ovarian cancer | Octanoylcarnitine | Weighted median | 5 | 0.03 | | 0.06 | 1.03 | 0.91 | 1.15 | 0.654 |  |  |
| High grade serous ovarian cancer | Octanoylcarnitine | MR Egger | 5 | 0.02 | | 0.10 | 1.02 | 0.83 | 1.24 | 0.888 |  | 0.777 |
| High grade serous ovarian cancer | Ornithine | Inverse variance weighted (multiplicative random effects) | 6 | -0.16 | | 0.26 | 0.85 | 0.51 | 1.42 | 0.538 | 0.000 |  |
| High grade serous ovarian cancer | Ornithine | Maximum likelihood | 6 | -0.18 | | 0.10 | 0.84 | 0.68 | 1.02 | 0.085 |  |  |
| High grade serous ovarian cancer | Ornithine | Simple median | 6 | -0.04 | | 0.13 | 0.96 | 0.74 | 1.25 | 0.770 |  |  |
| High grade serous ovarian cancer | Ornithine | Weighted median | 6 | 0.03 | | 0.14 | 1.03 | 0.79 | 1.34 | 0.852 |  |  |
| High grade serous ovarian cancer | Ornithine | MR Egger | 6 | 0.27 | | 0.68 | 1.31 | 0.35 | 4.95 | 0.709 |  | 0.525 |
| High grade serous ovarian cancer | Phenylalanine | Inverse variance weighted (fixed effects) | 6 | 0.23 | | 0.13 | 1.25 | 0.98 | 1.61 | 0.076 | 0.883 |  |
| High grade serous ovarian cancer | Phenylalanine | Maximum likelihood | 6 | 0.23 | | 0.13 | 1.26 | 0.98 | 1.61 | 0.077 |  |  |
| High grade serous ovarian cancer | Phenylalanine | Simple median | 6 | 0.16 | | 0.16 | 1.17 | 0.85 | 1.61 | 0.332 |  |  |
| High grade serous ovarian cancer | Phenylalanine | Weighted median | 6 | 0.23 | | 0.16 | 1.26 | 0.93 | 1.72 | 0.138 |  |  |
| High grade serous ovarian cancer | Phenylalanine | MR Egger | 6 | 0.42 | | 0.51 | 1.52 | 0.56 | 4.12 | 0.458 |  | 0.718 |
| High grade serous ovarian cancer | Proline | Inverse variance weighted (fixed effects) | 2 | 0.05 | | 0.06 | 1.05 | 0.93 | 1.18 | 0.432 | 0.294 |  |
| High grade serous ovarian cancer | Proline | Maximum likelihood | 2 | 0.05 | | 0.06 | 1.05 | 0.93 | 1.18 | 0.432 |  |  |
| High grade serous ovarian cancer | Propionylcarnitine | Inverse variance weighted (fixed effects) | 4 | -0.04 | | 0.06 | 0.96 | 0.86 | 1.08 | 0.515 | 0.700 |  |
| High grade serous ovarian cancer | Propionylcarnitine | Maximum likelihood | 4 | -0.04 | | 0.06 | 0.96 | 0.86 | 1.08 | 0.515 |  |  |
| High grade serous ovarian cancer | Propionylcarnitine | Simple median | 4 | 0.00 | | 0.09 | 1.00 | 0.83 | 1.20 | 0.983 |  |  |
| High grade serous ovarian cancer | Propionylcarnitine | Weighted median | 4 | -0.03 | | 0.06 | 0.97 | 0.86 | 1.10 | 0.672 |  |  |
| High grade serous ovarian cancer | Propionylcarnitine | MR Egger | 4 | 0.03 | | 0.13 | 1.03 | 0.79 | 1.34 | 0.857 |  | 0.647 |
| High grade serous ovarian cancer | Sarcosine | Inverse variance weighted (fixed effects) | 2 | -0.01 | | 0.14 | 0.99 | 0.75 | 1.30 | 0.945 | 0.514 |  |
| High grade serous ovarian cancer | Sarcosine | Maximum likelihood | 2 | -0.01 | | 0.14 | 0.99 | 0.75 | 1.30 | 0.945 |  |  |
| High grade serous ovarian cancer | Serine | Inverse variance weighted (fixed effects) | 4 | 0.07 | | 0.06 | 1.08 | 0.96 | 1.20 | 0.187 | 0.330 |  |
| High grade serous ovarian cancer | Serine | Maximum likelihood | 4 | 0.07 | | 0.06 | 1.08 | 0.96 | 1.20 | 0.187 |  |  |
| High grade serous ovarian cancer | Serine | Simple median | 4 | 0.07 | | 0.06 | 1.08 | 0.95 | 1.22 | 0.242 |  |  |
| High grade serous ovarian cancer | Serine | Weighted median | 4 | 0.02 | | 0.07 | 1.02 | 0.89 | 1.17 | 0.746 |  |  |
| High grade serous ovarian cancer | Serine | MR Egger | 4 | -0.28 | | 0.22 | 0.75 | 0.49 | 1.15 | 0.321 |  | 0.230 |
| High grade serous ovarian cancer | Spermidine | Wald ratio | 1 | 0.04 | | 0.10 | 1.04 | 0.86 | 1.26 | 0.664 | NA | NA |
| High grade serous ovarian cancer | Symmetric dimethylarginine | Wald ratio | 1 | -0.07 | | 0.14 | 0.93 | 0.71 | 1.22 | 0.597 | NA | NA |
| High grade serous ovarian cancer | Taurine | Wald ratio | 1 | -0.01 | | 0.12 | 0.99 | 0.79 | 1.25 | 0.949 | NA | NA |
| High grade serous ovarian cancer | Tetradecanoylcarnitine | Inverse variance weighted (fixed effects) | 3 | -0.21 | | 0.10 | 0.81 | 0.66 | 0.99 | 0.041 | 0.696 |  |
| High grade serous ovarian cancer | Tetradecanoylcarnitine | Maximum likelihood | 3 | -0.21 | | 0.10 | 0.81 | 0.66 | 0.99 | 0.042 |  |  |
| High grade serous ovarian cancer | Tetradecanoylcarnitine | Simple median | 3 | -0.22 | | 0.13 | 0.80 | 0.62 | 1.03 | 0.089 |  |  |
| High grade serous ovarian cancer | Tetradecanoylcarnitine | Weighted median | 3 | -0.24 | | 0.11 | 0.79 | 0.63 | 0.99 | 0.039 |  |  |
| High grade serous ovarian cancer | Tetradecanoylcarnitine | MR Egger | 3 | -0.74 | | 0.68 | 0.48 | 0.13 | 1.79 | 0.471 |  | 0.574 |
| High grade serous ovarian cancer | Tetradecenoylcarnitine | Inverse variance weighted (fixed effects) | 2 | -0.32 | | 0.18 | 0.73 | 0.52 | 1.03 | 0.071 | 0.636 |  |
| High grade serous ovarian cancer | Tetradecenoylcarnitine | Maximum likelihood | 2 | -0.32 | | 0.18 | 0.73 | 0.51 | 1.03 | 0.076 |  |  |
| High grade serous ovarian cancer | Threonine | Inverse variance weighted (fixed effects) | 3 | 0.11 | | 0.10 | 1.12 | 0.92 | 1.37 | 0.268 | 0.196 |  |
| High grade serous ovarian cancer | Threonine | Maximum likelihood | 3 | 0.12 | | 0.10 | 1.12 | 0.92 | 1.38 | 0.266 |  |  |
| High grade serous ovarian cancer | Threonine | Simple median | 3 | -0.03 | | 0.17 | 0.97 | 0.69 | 1.37 | 0.880 |  |  |
| High grade serous ovarian cancer | Threonine | Weighted median | 3 | 0.12 | | 0.11 | 1.13 | 0.90 | 1.41 | 0.301 |  |  |
| High grade serous ovarian cancer | Threonine | MR Egger | 3 | 0.80 | | 0.45 | 2.23 | 0.92 | 5.40 | 0.326 |  | 0.362 |
| High grade serous ovarian cancer | trans-Hydroxyproline | Wald ratio | 1 | -0.09 | | 0.20 | 0.91 | 0.62 | 1.35 | 0.643 | NA | NA |
| High grade serous ovarian cancer | Tryptophan | Inverse variance weighted (fixed effects) | 2 | -0.06 | | 0.12 | 0.95 | 0.75 | 1.19 | 0.636 | 0.368 |  |
| High grade serous ovarian cancer | Tryptophan | Maximum likelihood | 2 | -0.06 | | 0.12 | 0.95 | 0.75 | 1.19 | 0.636 |  |  |
| High grade serous ovarian cancer | Tyrosine | Inverse variance weighted (fixed effects) | 2 | -0.02 | | 0.12 | 0.98 | 0.78 | 1.23 | 0.851 | 0.927 |  |
| High grade serous ovarian cancer | Tyrosine | Maximum likelihood | 2 | -0.02 | | 0.12 | 0.98 | 0.78 | 1.23 | 0.851 |  |  |
| High grade serous ovarian cancer | Valerylcarnitine | Inverse variance weighted (fixed effects) | 3 | 0.01 | | 0.07 | 1.01 | 0.88 | 1.16 | 0.873 | 0.052 |  |
| High grade serous ovarian cancer | Valerylcarnitine | Maximum likelihood | 3 | 0.01 | | 0.07 | 1.01 | 0.88 | 1.16 | 0.872 |  |  |
| High grade serous ovarian cancer | Valerylcarnitine | Simple median | 3 | -0.02 | | 0.10 | 0.98 | 0.80 | 1.21 | 0.881 |  |  |
| High grade serous ovarian cancer | Valerylcarnitine | Weighted median | 3 | -0.05 | | 0.08 | 0.95 | 0.81 | 1.11 | 0.520 |  |  |
| High grade serous ovarian cancer | Valerylcarnitine | MR Egger | 3 | -0.58 | | 0.26 | 0.56 | 0.34 | 0.94 | 0.270 |  | 0.258 |
| High grade serous ovarian cancer | Valine | Inverse variance weighted (fixed effects) | 4 | 0.24 | | 0.15 | 1.27 | 0.96 | 1.69 | 0.100 | 0.781 |  |
| High grade serous ovarian cancer | Valine | Maximum likelihood | 4 | 0.24 | | 0.15 | 1.27 | 0.95 | 1.69 | 0.101 |  |  |
| High grade serous ovarian cancer | Valine | Simple median | 4 | 0.24 | | 0.18 | 1.27 | 0.90 | 1.80 | 0.169 |  |  |
| High grade serous ovarian cancer | Valine | Weighted median | 4 | 0.24 | | 0.16 | 1.27 | 0.93 | 1.74 | 0.133 |  |  |
| High grade serous ovarian cancer | Valine | MR Egger | 4 | -0.05 | | 0.74 | 0.95 | 0.22 | 4.02 | 0.950 |  | 0.725 |

| **Supplementary Table 5.** The association between exposure and Low malignant potential ovarian cancer. | | | | | | | | | | | |
| --- | --- | --- | --- | --- | --- | --- | --- | --- | --- | --- | --- |
| Outcome | Exposure | Method | No. | beta | se | OR | LCI | UCI | *P*-value | *P* for heterogenity | *P* for pleiotropy |
| Low malignant potential ovarian cancer | Acetylcarnitine | Inverse variance weighted (multiplicative random effects) | 3 | -0.04 | 0.33 | 0.96 | 0.50 | 1.83 | 0.899 | 0.043 |  |
| Low malignant potential ovarian cancer | Acetylcarnitine | Maximum likelihood | 3 | -0.04 | 0.19 | 0.96 | 0.67 | 1.38 | 0.821 |  |  |
| Low malignant potential ovarian cancer | Acetylcarnitine | Simple median | 3 | 0.12 | 0.29 | 1.13 | 0.65 | 1.98 | 0.670 |  |  |
| Low malignant potential ovarian cancer | Acetylcarnitine | Weighted median | 3 | -0.03 | 0.20 | 0.97 | 0.65 | 1.45 | 0.899 |  |  |
| Low malignant potential ovarian cancer | Acetylcarnitine | MR Egger | 3 | 1.01 | 0.47 | 2.75 | 1.10 | 6.88 | 0.277 |  | 0.247 |
| Low malignant potential ovarian cancer | Acetylornithine | Inverse variance weighted (fixed effects) | 2 | -0.03 | 0.07 | 0.97 | 0.85 | 1.11 | 0.649 | 0.818 |  |
| Low malignant potential ovarian cancer | Acetylornithine | Maximum likelihood | 2 | -0.03 | 0.07 | 0.97 | 0.85 | 1.11 | 0.649 |  |  |
| Low malignant potential ovarian cancer | Alanine | Inverse variance weighted (fixed effects) | 9 | 0.54 | 0.39 | 1.71 | 0.79 | 3.71 | 0.174 | 0.078 |  |
| Low malignant potential ovarian cancer | Alanine | Maximum likelihood | 9 | 0.55 | 0.40 | 1.74 | 0.79 | 3.82 | 0.168 |  |  |
| Low malignant potential ovarian cancer | Alanine | Simple median | 9 | 0.19 | 0.62 | 1.21 | 0.36 | 4.09 | 0.762 |  |  |
| Low malignant potential ovarian cancer | Alanine | Weighted median | 9 | 0.12 | 0.58 | 1.12 | 0.36 | 3.48 | 0.838 |  |  |
| Low malignant potential ovarian cancer | Alanine | MR Egger | 9 | -0.17 | 2.21 | 0.84 | 0.01 | 63.88 | 0.941 |  | 0.750 |
| Low malignant potential ovarian cancer | alpha-Aminoadipic acid | Inverse variance weighted (fixed effects) | 2 | -0.51 | 0.41 | 0.60 | 0.27 | 1.35 | 0.218 | 0.088 |  |
| Low malignant potential ovarian cancer | alpha-Aminoadipic acid | Maximum likelihood | 2 | -0.52 | 0.42 | 0.59 | 0.26 | 1.35 | 0.215 |  |  |
| Low malignant potential ovarian cancer | Arginine | Inverse variance weighted (fixed effects) | 7 | -0.46 | 0.21 | 0.63 | 0.42 | 0.95 | 0.028 | 0.871 |  |
| Low malignant potential ovarian cancer | Arginine | Maximum likelihood | 7 | -0.46 | 0.21 | 0.63 | 0.42 | 0.95 | 0.029 |  |  |
| Low malignant potential ovarian cancer | Arginine | Simple median | 7 | -0.37 | 0.29 | 0.69 | 0.39 | 1.22 | 0.201 |  |  |
| Low malignant potential ovarian cancer | Arginine | Weighted median | 7 | -0.57 | 0.25 | 0.57 | 0.34 | 0.93 | 0.025 |  |  |
| Low malignant potential ovarian cancer | Arginine | MR Egger | 7 | -0.64 | 0.35 | 0.53 | 0.27 | 1.04 | 0.124 |  | 0.537 |
| Low malignant potential ovarian cancer | Asparagine | Inverse variance weighted (multiplicative random effects) | 4 | -0.26 | 0.30 | 0.77 | 0.42 | 1.39 | 0.387 | 0.007 |  |
| Low malignant potential ovarian cancer | Asparagine | Maximum likelihood | 4 | -0.27 | 0.15 | 0.77 | 0.57 | 1.03 | 0.082 |  |  |
| Low malignant potential ovarian cancer | Asparagine | Simple median | 4 | -0.30 | 0.34 | 0.74 | 0.38 | 1.45 | 0.382 |  |  |
| Low malignant potential ovarian cancer | Asparagine | Weighted median | 4 | -0.26 | 0.16 | 0.77 | 0.57 | 1.05 | 0.096 |  |  |
| Low malignant potential ovarian cancer | Asparagine | MR Egger | 4 | -0.07 | 0.48 | 0.93 | 0.36 | 2.37 | 0.893 |  | 0.624 |
| Low malignant potential ovarian cancer | Aspartate | Inverse variance weighted (fixed effects) | 2 | 0.02 | 0.29 | 1.02 | 0.58 | 1.80 | 0.935 | 0.504 |  |
| Low malignant potential ovarian cancer | Aspartate | Maximum likelihood | 2 | 0.02 | 0.29 | 1.02 | 0.58 | 1.80 | 0.935 |  |  |
| Low malignant potential ovarian cancer | Butyrylcarnitine | Inverse variance weighted (fixed effects) | 2 | 0.24 | 0.26 | 1.27 | 0.76 | 2.12 | 0.362 | 0.634 |  |
| Low malignant potential ovarian cancer | Butyrylcarnitine | Maximum likelihood | 2 | 0.24 | 0.26 | 1.27 | 0.76 | 2.12 | 0.362 |  |  |
| Low malignant potential ovarian cancer | Carnitine | Inverse variance weighted (fixed effects) | 5 | 0.12 | 0.12 | 1.12 | 0.89 | 1.42 | 0.337 | 0.883 |  |
| Low malignant potential ovarian cancer | Carnitine | Maximum likelihood | 5 | 0.12 | 0.12 | 1.12 | 0.89 | 1.42 | 0.337 |  |  |
| Low malignant potential ovarian cancer | Carnitine | Simple median | 5 | 0.44 | 0.27 | 1.55 | 0.91 | 2.63 | 0.108 |  |  |
| Low malignant potential ovarian cancer | Carnitine | Weighted median | 5 | 0.08 | 0.13 | 1.08 | 0.84 | 1.39 | 0.549 |  |  |
| Low malignant potential ovarian cancer | Carnitine | MR Egger | 5 | 0.06 | 0.22 | 1.06 | 0.69 | 1.64 | 0.803 |  | 0.785 |
| Low malignant potential ovarian cancer | Citrulline | Inverse variance weighted (fixed effects) | 4 | -0.14 | 0.30 | 0.87 | 0.48 | 1.55 | 0.631 | 0.642 |  |
| Low malignant potential ovarian cancer | Citrulline | Maximum likelihood | 4 | -0.14 | 0.30 | 0.87 | 0.48 | 1.56 | 0.630 |  |  |
| Low malignant potential ovarian cancer | Citrulline | Simple median | 4 | -0.13 | 0.35 | 0.88 | 0.44 | 1.75 | 0.706 |  |  |
| Low malignant potential ovarian cancer | Citrulline | Weighted median | 4 | 0.03 | 0.36 | 1.03 | 0.51 | 2.11 | 0.926 |  |  |
| Low malignant potential ovarian cancer | Citrulline | MR Egger | 4 | -0.07 | 1.98 | 0.93 | 0.02 | 44.89 | 0.974 |  | 0.974 |
| Low malignant potential ovarian cancer | Creatinine | Inverse variance weighted (fixed effects) | 12 | -0.02 | 0.38 | 0.98 | 0.46 | 2.06 | 0.954 | 0.099 |  |
| Low malignant potential ovarian cancer | Creatinine | Maximum likelihood | 12 | -0.02 | 0.39 | 0.98 | 0.46 | 2.08 | 0.956 |  |  |
| Low malignant potential ovarian cancer | Creatinine | Simple median | 12 | 0.21 | 0.54 | 1.23 | 0.43 | 3.53 | 0.694 |  |  |
| Low malignant potential ovarian cancer | Creatinine | Weighted median | 12 | 0.19 | 0.52 | 1.21 | 0.43 | 3.39 | 0.713 |  |  |
| Low malignant potential ovarian cancer | Creatinine | MR Egger | 12 | 0.09 | 2.35 | 1.10 | 0.01 | 110.72 | 0.969 |  | 0.961 |
| Low malignant potential ovarian cancer | Decanoylcarnitine | Inverse variance weighted (fixed effects) | 2 | -0.05 | 0.18 | 0.95 | 0.66 | 1.37 | 0.788 | 0.278 |  |
| Low malignant potential ovarian cancer | Decanoylcarnitine | Maximum likelihood | 2 | -0.05 | 0.18 | 0.95 | 0.66 | 1.37 | 0.788 |  |  |
| Low malignant potential ovarian cancer | Decenoylcarnitine | Inverse variance weighted (fixed effects) | 3 | -0.19 | 0.22 | 0.83 | 0.54 | 1.28 | 0.401 | 0.355 |  |
| Low malignant potential ovarian cancer | Decenoylcarnitine | Maximum likelihood | 3 | -0.19 | 0.22 | 0.83 | 0.54 | 1.28 | 0.400 |  |  |
| Low malignant potential ovarian cancer | Decenoylcarnitine | Simple median | 3 | -0.26 | 0.28 | 0.77 | 0.45 | 1.32 | 0.338 |  |  |
| Low malignant potential ovarian cancer | Decenoylcarnitine | Weighted median | 3 | -0.18 | 0.25 | 0.83 | 0.51 | 1.36 | 0.464 |  |  |
| Low malignant potential ovarian cancer | Decenoylcarnitine | MR Egger | 3 | 0.06 | 1.14 | 1.06 | 0.11 | 9.95 | 0.968 |  | 0.862 |
| Low malignant potential ovarian cancer | Dodecanoylcarnitine | Wald ratio | 1 | -2.10 | 0.91 | 0.12 | 0.02 | 0.74 | 0.022 | NA | NA |
| Low malignant potential ovarian cancer | Glutamine | Inverse variance weighted (multiplicative random effects) | 5 | -0.10 | 0.78 | 0.91 | 0.20 | 4.18 | 0.902 | 0.007 |  |
| Low malignant potential ovarian cancer | Glutamine | Maximum likelihood | 5 | -0.10 | 0.42 | 0.91 | 0.40 | 2.08 | 0.817 |  |  |
| Low malignant potential ovarian cancer | Glutamine | Simple median | 5 | 0.85 | 0.63 | 2.35 | 0.68 | 8.09 | 0.175 |  |  |
| Low malignant potential ovarian cancer | Glutamine | Weighted median | 5 | 0.54 | 0.62 | 1.72 | 0.51 | 5.79 | 0.381 |  |  |
| Low malignant potential ovarian cancer | Glutamine | MR Egger | 5 | -2.64 | 3.57 | 0.07 | 0.00 | 79.08 | 0.514 |  | 0.518 |
| Low malignant potential ovarian cancer | Glycine | Inverse variance weighted (fixed effects) | 12 | 0.08 | 0.11 | 1.08 | 0.87 | 1.33 | 0.479 | 0.398 |  |
| Low malignant potential ovarian cancer | Glycine | Maximum likelihood | 12 | 0.08 | 0.11 | 1.08 | 0.87 | 1.33 | 0.474 |  |  |
| Low malignant potential ovarian cancer | Glycine | Simple median | 12 | 0.29 | 0.29 | 1.34 | 0.76 | 2.36 | 0.317 |  |  |
| Low malignant potential ovarian cancer | Glycine | Weighted median | 12 | 0.09 | 0.11 | 1.09 | 0.87 | 1.36 | 0.450 |  |  |
| Low malignant potential ovarian cancer | Glycine | MR Egger | 12 | 0.05 | 0.15 | 1.05 | 0.79 | 1.39 | 0.757 |  | 0.743 |
| Low malignant potential ovarian cancer | Hexadecanoylcarnitine | Inverse variance weighted (fixed effects) | 2 | -0.56 | 0.35 | 0.57 | 0.28 | 1.14 | 0.110 | 0.067 |  |
| Low malignant potential ovarian cancer | Hexadecanoylcarnitine | Maximum likelihood | 2 | -0.57 | 0.36 | 0.56 | 0.28 | 1.14 | 0.109 |  |  |
| Low malignant potential ovarian cancer | Hexadecenoylcarnitine | Wald ratio | 1 | -0.44 | 0.54 | 0.64 | 0.22 | 1.85 | 0.414 | NA | NA |
| Low malignant potential ovarian cancer | Hexanoylcarnitine | Inverse variance weighted (fixed effects) | 3 | 0.03 | 0.16 | 1.03 | 0.75 | 1.42 | 0.856 | 0.679 |  |
| Low malignant potential ovarian cancer | Hexanoylcarnitine | Maximum likelihood | 3 | 0.03 | 0.16 | 1.03 | 0.75 | 1.42 | 0.856 |  |  |
| Low malignant potential ovarian cancer | Hexanoylcarnitine | Simple median | 3 | 0.17 | 0.23 | 1.19 | 0.76 | 1.86 | 0.447 |  |  |
| Low malignant potential ovarian cancer | Hexanoylcarnitine | Weighted median | 3 | 0.03 | 0.18 | 1.04 | 0.73 | 1.47 | 0.845 |  |  |
| Low malignant potential ovarian cancer | Hexanoylcarnitine | MR Egger | 3 | -0.11 | 0.23 | 0.89 | 0.57 | 1.41 | 0.710 |  | 0.542 |
| Low malignant potential ovarian cancer | Hexose | Wald ratio | 1 | 0.80 | 0.54 | 2.23 | 0.78 | 6.38 | 0.135 | NA | NA |
| Low malignant potential ovarian cancer | Histidine | Inverse variance weighted (fixed effects) | 7 | 0.06 | 0.35 | 1.06 | 0.54 | 2.11 | 0.861 | 0.066 |  |
| Low malignant potential ovarian cancer | Histidine | Maximum likelihood | 7 | 0.06 | 0.35 | 1.07 | 0.53 | 2.13 | 0.858 |  |  |
| Low malignant potential ovarian cancer | Histidine | Simple median | 7 | 0.15 | 0.53 | 1.16 | 0.41 | 3.28 | 0.777 |  |  |
| Low malignant potential ovarian cancer | Histidine | Weighted median | 7 | 0.60 | 0.46 | 1.82 | 0.73 | 4.51 | 0.198 |  |  |
| Low malignant potential ovarian cancer | Histidine | MR Egger | 7 | 4.39 | 2.23 | 80.29 | 1.01 | 6390.22 | 0.107 |  | 0.106 |
| Low malignant potential ovarian cancer | Isoleucine | Inverse variance weighted (multiplicative random effects) | 3 | 0.23 | 1.38 | 1.26 | 0.08 | 18.64 | 0.867 | 0.024 |  |
| Low malignant potential ovarian cancer | Isoleucine | Maximum likelihood | 3 | 0.24 | 0.73 | 1.27 | 0.30 | 5.29 | 0.743 |  |  |
| Low malignant potential ovarian cancer | Isoleucine | Simple median | 3 | 1.05 | 0.95 | 2.86 | 0.45 | 18.35 | 0.267 |  |  |
| Low malignant potential ovarian cancer | Isoleucine | Weighted median | 3 | 1.14 | 0.86 | 3.12 | 0.58 | 16.86 | 0.186 |  |  |
| Low malignant potential ovarian cancer | Isoleucine | MR Egger | 3 | 7.51 | 4.62 | 1826.47 | 0.21 | 15713360.17 | 0.351 |  | 0.353 |
| Low malignant potential ovarian cancer | Kynurenine | Inverse variance weighted (fixed effects) | 3 | 0.17 | 0.23 | 1.19 | 0.76 | 1.86 | 0.444 | 0.828 |  |
| Low malignant potential ovarian cancer | Kynurenine | Maximum likelihood | 3 | 0.17 | 0.23 | 1.19 | 0.76 | 1.86 | 0.444 |  |  |
| Low malignant potential ovarian cancer | Kynurenine | Simple median | 3 | 0.16 | 0.28 | 1.17 | 0.67 | 2.04 | 0.579 |  |  |
| Low malignant potential ovarian cancer | Kynurenine | Weighted median | 3 | 0.16 | 0.25 | 1.17 | 0.72 | 1.90 | 0.523 |  |  |
| Low malignant potential ovarian cancer | Kynurenine | MR Egger | 3 | 0.22 | 0.66 | 1.24 | 0.34 | 4.56 | 0.800 |  | 0.957 |
| Low malignant potential ovarian cancer | Leucine | Inverse variance weighted (fixed effects) | 4 | 1.45 | 0.64 | 4.25 | 1.22 | 14.83 | 0.023 | 0.064 |  |
| Low malignant potential ovarian cancer | Leucine | Maximum likelihood | 4 | 1.49 | 0.66 | 4.45 | 1.23 | 16.08 | 0.023 |  |  |
| Low malignant potential ovarian cancer | Leucine | Simple median | 4 | 1.58 | 0.85 | 4.84 | 0.91 | 25.83 | 0.065 |  |  |
| Low malignant potential ovarian cancer | Leucine | Weighted median | 4 | 1.47 | 0.85 | 4.35 | 0.82 | 23.06 | 0.084 |  |  |
| Low malignant potential ovarian cancer | Leucine | MR Egger | 4 | -1.64 | 3.18 | 0.19 | 0.00 | 99.92 | 0.659 |  | 0.416 |
| Low malignant potential ovarian cancer | Lysine | Inverse variance weighted (fixed effects) | 6 | -0.10 | 0.20 | 0.90 | 0.62 | 1.33 | 0.605 | 0.217 |  |
| Low malignant potential ovarian cancer | Lysine | Maximum likelihood | 6 | -0.10 | 0.20 | 0.90 | 0.62 | 1.33 | 0.604 |  |  |
| Low malignant potential ovarian cancer | Lysine | Simple median | 6 | -0.42 | 0.32 | 0.65 | 0.35 | 1.23 | 0.190 |  |  |
| Low malignant potential ovarian cancer | Lysine | Weighted median | 6 | 0.04 | 0.25 | 1.04 | 0.64 | 1.68 | 0.885 |  |  |
| Low malignant potential ovarian cancer | Lysine | MR Egger | 6 | 0.53 | 0.50 | 1.70 | 0.64 | 4.48 | 0.345 |  | 0.231 |
| Low malignant potential ovarian cancer | Methionine | Wald ratio | 1 | -0.05 | 0.82 | 0.95 | 0.19 | 4.73 | 0.954 | NA | NA |
| Low malignant potential ovarian cancer | Methioninesulfoxide | Wald ratio | 1 | -0.60 | 0.68 | 0.55 | 0.15 | 2.08 | 0.380 | NA | NA |
| Low malignant potential ovarian cancer | Methylglutarylcarnitine | Inverse variance weighted (fixed effects) | 4 | 0.05 | 0.20 | 1.06 | 0.71 | 1.56 | 0.786 | 0.739 |  |
| Low malignant potential ovarian cancer | Methylglutarylcarnitine | Maximum likelihood | 4 | 0.05 | 0.20 | 1.06 | 0.71 | 1.56 | 0.785 |  |  |
| Low malignant potential ovarian cancer | Methylglutarylcarnitine | Simple median | 4 | -0.08 | 0.28 | 0.92 | 0.53 | 1.61 | 0.773 |  |  |
| Low malignant potential ovarian cancer | Methylglutarylcarnitine | Weighted median | 4 | 0.17 | 0.21 | 1.19 | 0.79 | 1.80 | 0.406 |  |  |
| Low malignant potential ovarian cancer | Methylglutarylcarnitine | MR Egger | 4 | 0.34 | 0.58 | 1.41 | 0.45 | 4.40 | 0.613 |  | 0.647 |
| Low malignant potential ovarian cancer | Nonaylcarnitine | Inverse variance weighted (fixed effects) | 2 | -0.21 | 0.19 | 0.81 | 0.56 | 1.16 | 0.255 | 0.392 |  |
| Low malignant potential ovarian cancer | Nonaylcarnitine | Maximum likelihood | 2 | -0.21 | 0.19 | 0.81 | 0.56 | 1.16 | 0.255 |  |  |
| Low malignant potential ovarian cancer | Octadecandienylcarnitine | Inverse variance weighted (multiplicative random effects) | 4 | -0.19 | 0.30 | 0.82 | 0.46 | 1.48 | 0.515 | 0.046 |  |
| Low malignant potential ovarian cancer | Octadecandienylcarnitine | Maximum likelihood | 4 | -0.20 | 0.18 | 0.82 | 0.57 | 1.18 | 0.286 |  |  |
| Low malignant potential ovarian cancer | Octadecandienylcarnitine | Simple median | 4 | 0.07 | 0.30 | 1.07 | 0.59 | 1.94 | 0.819 |  |  |
| Low malignant potential ovarian cancer | Octadecandienylcarnitine | Weighted median | 4 | -0.19 | 0.21 | 0.83 | 0.55 | 1.25 | 0.365 |  |  |
| Low malignant potential ovarian cancer | Octadecandienylcarnitine | MR Egger | 4 | -0.13 | 0.88 | 0.88 | 0.16 | 4.88 | 0.894 |  | 0.945 |
| Low malignant potential ovarian cancer | Octadecanoylcarnitine | Inverse variance weighted (fixed effects) | 5 | -0.03 | 0.28 | 0.97 | 0.56 | 1.69 | 0.910 | 0.096 |  |
| Low malignant potential ovarian cancer | Octadecanoylcarnitine | Maximum likelihood | 5 | -0.03 | 0.29 | 0.97 | 0.55 | 1.70 | 0.909 |  |  |
| Low malignant potential ovarian cancer | Octadecanoylcarnitine | Simple median | 5 | 0.35 | 0.43 | 1.42 | 0.62 | 3.29 | 0.410 |  |  |
| Low malignant potential ovarian cancer | Octadecanoylcarnitine | Weighted median | 5 | 0.26 | 0.38 | 1.30 | 0.62 | 2.74 | 0.487 |  |  |
| Low malignant potential ovarian cancer | Octadecanoylcarnitine | MR Egger | 5 | -1.78 | 2.43 | 0.17 | 0.00 | 19.72 | 0.516 |  | 0.518 |
| Low malignant potential ovarian cancer | Octadecenoylcarnitine | Inverse variance weighted (fixed effects) | 2 | -0.50 | 0.25 | 0.61 | 0.37 | 1.00 | 0.049 | 0.124 |  |
| Low malignant potential ovarian cancer | Octadecenoylcarnitine | Maximum likelihood | 2 | -0.51 | 0.26 | 0.60 | 0.36 | 1.00 | 0.050 |  |  |
| Low malignant potential ovarian cancer | Octanoylcarnitine | Inverse variance weighted (fixed effects) | 5 | -0.05 | 0.15 | 0.95 | 0.71 | 1.26 | 0.709 | 0.487 |  |
| Low malignant potential ovarian cancer | Octanoylcarnitine | Maximum likelihood | 5 | -0.05 | 0.15 | 0.95 | 0.71 | 1.26 | 0.708 |  |  |
| Low malignant potential ovarian cancer | Octanoylcarnitine | Simple median | 5 | 0.18 | 0.26 | 1.20 | 0.72 | 2.00 | 0.487 |  |  |
| Low malignant potential ovarian cancer | Octanoylcarnitine | Weighted median | 5 | -0.07 | 0.17 | 0.94 | 0.68 | 1.30 | 0.687 |  |  |
| Low malignant potential ovarian cancer | Octanoylcarnitine | MR Egger | 5 | -0.06 | 0.33 | 0.94 | 0.50 | 1.80 | 0.872 |  | 0.991 |
| Low malignant potential ovarian cancer | Ornithine | Inverse variance weighted (fixed effects) | 6 | 0.27 | 0.30 | 1.31 | 0.74 | 2.34 | 0.358 | 0.165 |  |
| Low malignant potential ovarian cancer | Ornithine | Maximum likelihood | 6 | 0.28 | 0.30 | 1.32 | 0.73 | 2.38 | 0.352 |  |  |
| Low malignant potential ovarian cancer | Ornithine | Simple median | 6 | 0.08 | 0.40 | 1.08 | 0.50 | 2.35 | 0.847 |  |  |
| Low malignant potential ovarian cancer | Ornithine | Weighted median | 6 | 0.79 | 0.41 | 2.21 | 0.98 | 4.97 | 0.055 |  |  |
| Low malignant potential ovarian cancer | Ornithine | MR Egger | 6 | 1.83 | 0.71 | 6.26 | 1.55 | 25.25 | 0.062 |  | 0.073 |
| Low malignant potential ovarian cancer | Phenylalanine | Inverse variance weighted (fixed effects) | 6 | 0.56 | 0.39 | 1.75 | 0.81 | 3.76 | 0.153 | 0.558 |  |
| Low malignant potential ovarian cancer | Phenylalanine | Maximum likelihood | 6 | 0.56 | 0.39 | 1.75 | 0.81 | 3.79 | 0.153 |  |  |
| Low malignant potential ovarian cancer | Phenylalanine | Simple median | 6 | 0.58 | 0.52 | 1.79 | 0.64 | 4.99 | 0.263 |  |  |
| Low malignant potential ovarian cancer | Phenylalanine | Weighted median | 6 | 0.67 | 0.48 | 1.96 | 0.77 | 5.03 | 0.159 |  |  |
| Low malignant potential ovarian cancer | Phenylalanine | MR Egger | 6 | 0.71 | 1.55 | 2.04 | 0.10 | 42.34 | 0.669 |  | 0.923 |
| Low malignant potential ovarian cancer | Proline | Inverse variance weighted (fixed effects) | 2 | -0.05 | 0.18 | 0.96 | 0.67 | 1.36 | 0.799 | 0.954 |  |
| Low malignant potential ovarian cancer | Proline | Maximum likelihood | 2 | -0.05 | 0.18 | 0.96 | 0.67 | 1.36 | 0.799 |  |  |
| Low malignant potential ovarian cancer | Propionylcarnitine | Inverse variance weighted (fixed effects) | 4 | -0.03 | 0.18 | 0.97 | 0.69 | 1.38 | 0.884 | 0.051 |  |
| Low malignant potential ovarian cancer | Propionylcarnitine | Maximum likelihood | 4 | -0.03 | 0.18 | 0.97 | 0.69 | 1.38 | 0.884 |  |  |
| Low malignant potential ovarian cancer | Propionylcarnitine | Simple median | 4 | 0.48 | 0.34 | 1.62 | 0.84 | 3.12 | 0.153 |  |  |
| Low malignant potential ovarian cancer | Propionylcarnitine | Weighted median | 4 | -0.02 | 0.21 | 0.98 | 0.66 | 1.47 | 0.938 |  |  |
| Low malignant potential ovarian cancer | Propionylcarnitine | MR Egger | 4 | 0.28 | 0.78 | 1.33 | 0.29 | 6.08 | 0.750 |  | 0.703 |
| Low malignant potential ovarian cancer | Sarcosine | Inverse variance weighted (fixed effects) | 2 | -0.80 | 0.41 | 0.45 | 0.20 | 1.00 | 0.050 | 0.577 |  |
| Low malignant potential ovarian cancer | Sarcosine | Maximum likelihood | 2 | -0.80 | 0.41 | 0.45 | 0.20 | 1.01 | 0.052 |  |  |
| Low malignant potential ovarian cancer | Serine | Inverse variance weighted (multiplicative random effects) | 4 | 0.08 | 0.33 | 1.08 | 0.56 | 2.08 | 0.818 | 0.009 |  |
| Low malignant potential ovarian cancer | Serine | Maximum likelihood | 4 | 0.08 | 0.17 | 1.08 | 0.77 | 1.51 | 0.650 |  |  |
| Low malignant potential ovarian cancer | Serine | Simple median | 4 | 0.21 | 0.19 | 1.24 | 0.85 | 1.80 | 0.261 |  |  |
| Low malignant potential ovarian cancer | Serine | Weighted median | 4 | 0.24 | 0.20 | 1.28 | 0.86 | 1.90 | 0.227 |  |  |
| Low malignant potential ovarian cancer | Serine | MR Egger | 4 | 2.22 | 0.66 | 9.20 | 2.53 | 33.42 | 0.078 |  | 0.078 |
| Low malignant potential ovarian cancer | Spermidine | Wald ratio | 1 | -0.53 | 0.29 | 0.59 | 0.33 | 1.04 | 0.070 | NA | NA |
| Low malignant potential ovarian cancer | Symmetric dimethylarginine | Wald ratio | 1 | 0.06 | 0.42 | 1.06 | 0.47 | 2.38 | 0.894 | NA | NA |
| Low malignant potential ovarian cancer | Taurine | Wald ratio | 1 | 0.38 | 0.35 | 1.46 | 0.74 | 2.88 | 0.275 | NA | NA |
| Low malignant potential ovarian cancer | Tetradecanoylcarnitine | Inverse variance weighted (fixed effects) | 3 | -0.48 | 0.31 | 0.62 | 0.33 | 1.15 | 0.127 | 0.337 |  |
| Low malignant potential ovarian cancer | Tetradecanoylcarnitine | Maximum likelihood | 3 | -0.48 | 0.32 | 0.62 | 0.33 | 1.15 | 0.127 |  |  |
| Low malignant potential ovarian cancer | Tetradecanoylcarnitine | Simple median | 3 | -0.42 | 0.40 | 0.66 | 0.30 | 1.43 | 0.292 |  |  |
| Low malignant potential ovarian cancer | Tetradecanoylcarnitine | Weighted median | 3 | -0.57 | 0.37 | 0.57 | 0.28 | 1.16 | 0.120 |  |  |
| Low malignant potential ovarian cancer | Tetradecanoylcarnitine | MR Egger | 3 | -3.21 | 2.05 | 0.04 | 0.00 | 2.25 | 0.362 |  | 0.406 |
| Low malignant potential ovarian cancer | Tetradecenoylcarnitine | Inverse variance weighted (multiplicative random effects) | 2 | -0.64 | 1.11 | 0.53 | 0.06 | 4.64 | 0.564 | 0.039 |  |
| Low malignant potential ovarian cancer | Tetradecenoylcarnitine | Maximum likelihood | 2 | -0.67 | 0.55 | 0.51 | 0.17 | 1.52 | 0.227 |  |  |
| Low malignant potential ovarian cancer | Threonine | Inverse variance weighted (fixed effects) | 3 | -0.88 | 0.31 | 0.42 | 0.23 | 0.77 | 0.005 | 0.352 |  |
| Low malignant potential ovarian cancer | Threonine | Maximum likelihood | 3 | -0.88 | 0.32 | 0.41 | 0.22 | 0.77 | 0.005 |  |  |
| Low malignant potential ovarian cancer | Threonine | Simple median | 3 | -0.41 | 0.48 | 0.66 | 0.26 | 1.70 | 0.390 |  |  |
| Low malignant potential ovarian cancer | Threonine | Weighted median | 3 | -0.82 | 0.34 | 0.44 | 0.22 | 0.86 | 0.017 |  |  |
| Low malignant potential ovarian cancer | Threonine | MR Egger | 3 | -2.67 | 1.37 | 0.07 | 0.00 | 1.01 | 0.301 |  | 0.406 |
| Low malignant potential ovarian cancer | trans-Hydroxyproline | Wald ratio | 1 | -0.39 | 0.60 | 0.68 | 0.21 | 2.20 | 0.515 | NA | NA |
| Low malignant potential ovarian cancer | Tryptophan | Inverse variance weighted (fixed effects) | 2 | 0.26 | 0.36 | 1.30 | 0.65 | 2.63 | 0.460 | 0.202 |  |
| Low malignant potential ovarian cancer | Tryptophan | Maximum likelihood | 2 | 0.27 | 0.36 | 1.31 | 0.64 | 2.65 | 0.459 |  |  |
| Low malignant potential ovarian cancer | Tyrosine | Inverse variance weighted (fixed effects) | 2 | 0.55 | 0.35 | 1.74 | 0.88 | 3.43 | 0.112 | 0.995 |  |
| Low malignant potential ovarian cancer | Tyrosine | Maximum likelihood | 2 | 0.55 | 0.35 | 1.74 | 0.88 | 3.44 | 0.112 |  |  |
| Low malignant potential ovarian cancer | Valerylcarnitine | Inverse variance weighted (fixed effects) | 3 | -0.30 | 0.21 | 0.74 | 0.49 | 1.12 | 0.154 | 0.139 |  |
| Low malignant potential ovarian cancer | Valerylcarnitine | Maximum likelihood | 3 | -0.31 | 0.22 | 0.74 | 0.48 | 1.12 | 0.153 |  |  |
| Low malignant potential ovarian cancer | Valerylcarnitine | Simple median | 3 | 0.15 | 0.35 | 1.17 | 0.58 | 2.32 | 0.663 |  |  |
| Low malignant potential ovarian cancer | Valerylcarnitine | Weighted median | 3 | -0.19 | 0.24 | 0.83 | 0.52 | 1.32 | 0.429 |  |  |
| Low malignant potential ovarian cancer | Valerylcarnitine | MR Egger | 3 | -1.61 | 0.86 | 0.20 | 0.04 | 1.07 | 0.311 |  | 0.359 |
| Low malignant potential ovarian cancer | Valine | Inverse variance weighted (fixed effects) | 4 | 0.29 | 0.45 | 1.33 | 0.56 | 3.20 | 0.521 | 0.681 |  |
| Low malignant potential ovarian cancer | Valine | Maximum likelihood | 4 | 0.29 | 0.45 | 1.33 | 0.55 | 3.21 | 0.520 |  |  |
| Low malignant potential ovarian cancer | Valine | Simple median | 4 | 0.18 | 0.60 | 1.20 | 0.37 | 3.85 | 0.762 |  |  |
| Low malignant potential ovarian cancer | Valine | Weighted median | 4 | 0.10 | 0.49 | 1.11 | 0.42 | 2.91 | 0.838 |  |  |
| Low malignant potential ovarian cancer | Valine | MR Egger | 4 | -0.22 | 2.28 | 0.80 | 0.01 | 69.94 | 0.931 |  | 0.840 |

| **Supplementary Table 6.** The association between exposure and Invasive mucinous ovarian cancer. | | | | | | | | | | | |
| --- | --- | --- | --- | --- | --- | --- | --- | --- | --- | --- | --- |
| Outcome | Exposure | Method | No. | beta | se | OR | LCI | UCI | *P*-value | *P* for heterogenity | *P* for pleiotropy |
| Invasive mucinous ovarian cancer | Acetylcarnitine | Inverse variance weighted (fixed effects) | 3 | -0.15 | 0.15 | 0.86 | 0.64 | 1.16 | 0.317 | 0.635 |  |
| Invasive mucinous ovarian cancer | Acetylcarnitine | Maximum likelihood | 3 | -0.15 | 0.15 | 0.86 | 0.64 | 1.16 | 0.317 |  |  |
| Invasive mucinous ovarian cancer | Acetylcarnitine | Simple median | 3 | -0.22 | 0.24 | 0.81 | 0.51 | 1.28 | 0.356 |  |  |
| Invasive mucinous ovarian cancer | Acetylcarnitine | Weighted median | 3 | -0.12 | 0.16 | 0.88 | 0.65 | 1.20 | 0.432 |  |  |
| Invasive mucinous ovarian cancer | Acetylcarnitine | MR Egger | 3 | -0.13 | 0.38 | 0.88 | 0.42 | 1.86 | 0.796 |  | 0.953 |
| Invasive mucinous ovarian cancer | Acetylornithine | Inverse variance weighted (fixed effects) | 2 | 0.01 | 0.06 | 1.01 | 0.90 | 1.13 | 0.925 | 0.318 |  |
| Invasive mucinous ovarian cancer | Acetylornithine | Maximum likelihood | 2 | 0.01 | 0.06 | 1.01 | 0.90 | 1.13 | 0.925 |  |  |
| Invasive mucinous ovarian cancer | Alanine | Inverse variance weighted (fixed effects) | 9 | -0.28 | 0.33 | 0.75 | 0.40 | 1.43 | 0.386 | 0.654 |  |
| Invasive mucinous ovarian cancer | Alanine | Maximum likelihood | 9 | -0.28 | 0.33 | 0.75 | 0.40 | 1.44 | 0.393 |  |  |
| Invasive mucinous ovarian cancer | Alanine | Simple median | 9 | -0.27 | 0.48 | 0.76 | 0.30 | 1.93 | 0.567 |  |  |
| Invasive mucinous ovarian cancer | Alanine | Weighted median | 9 | -0.27 | 0.44 | 0.76 | 0.32 | 1.80 | 0.539 |  |  |
| Invasive mucinous ovarian cancer | Alanine | MR Egger | 9 | -1.32 | 1.30 | 0.27 | 0.02 | 3.40 | 0.344 |  | 0.437 |
| Invasive mucinous ovarian cancer | alpha-Aminoadipic acid | Inverse variance weighted (fixed effects) | 2 | 0.74 | 0.37 | 2.09 | 1.02 | 4.28 | 0.045 | 0.811 |  |
| Invasive mucinous ovarian cancer | alpha-Aminoadipic acid | Maximum likelihood | 2 | 0.74 | 0.37 | 2.09 | 1.00 | 4.35 | 0.049 |  |  |
| Invasive mucinous ovarian cancer | Arginine | Inverse variance weighted (fixed effects) | 7 | -0.05 | 0.18 | 0.95 | 0.67 | 1.34 | 0.764 | 0.901 |  |
| Invasive mucinous ovarian cancer | Arginine | Maximum likelihood | 7 | -0.05 | 0.18 | 0.95 | 0.67 | 1.34 | 0.764 |  |  |
| Invasive mucinous ovarian cancer | Arginine | Simple median | 7 | 0.05 | 0.25 | 1.06 | 0.64 | 1.74 | 0.831 |  |  |
| Invasive mucinous ovarian cancer | Arginine | Weighted median | 7 | -0.20 | 0.22 | 0.82 | 0.54 | 1.26 | 0.367 |  |  |
| Invasive mucinous ovarian cancer | Arginine | MR Egger | 7 | -0.27 | 0.30 | 0.76 | 0.42 | 1.37 | 0.405 |  | 0.406 |
| Invasive mucinous ovarian cancer | Asparagine | Inverse variance weighted (fixed effects) | 4 | -0.14 | 0.12 | 0.87 | 0.68 | 1.11 | 0.250 | 0.517 |  |
| Invasive mucinous ovarian cancer | Asparagine | Maximum likelihood | 4 | -0.14 | 0.12 | 0.87 | 0.68 | 1.11 | 0.250 |  |  |
| Invasive mucinous ovarian cancer | Asparagine | Simple median | 4 | -0.36 | 0.25 | 0.70 | 0.43 | 1.14 | 0.155 |  |  |
| Invasive mucinous ovarian cancer | Asparagine | Weighted median | 4 | -0.14 | 0.13 | 0.87 | 0.67 | 1.13 | 0.302 |  |  |
| Invasive mucinous ovarian cancer | Asparagine | MR Egger | 4 | -0.07 | 0.17 | 0.93 | 0.66 | 1.30 | 0.711 |  | 0.623 |
| Invasive mucinous ovarian cancer | Aspartate | Inverse variance weighted (fixed effects) | 2 | 0.16 | 0.24 | 1.18 | 0.74 | 1.87 | 0.484 | 0.536 |  |
| Invasive mucinous ovarian cancer | Aspartate | Maximum likelihood | 2 | 0.17 | 0.24 | 1.18 | 0.74 | 1.87 | 0.484 |  |  |
| Invasive mucinous ovarian cancer | Butyrylcarnitine | Inverse variance weighted (fixed effects) | 2 | -0.06 | 0.22 | 0.94 | 0.62 | 1.43 | 0.773 | 0.615 |  |
| Invasive mucinous ovarian cancer | Butyrylcarnitine | Maximum likelihood | 2 | -0.06 | 0.22 | 0.94 | 0.62 | 1.43 | 0.773 |  |  |
| Invasive mucinous ovarian cancer | Carnitine | Inverse variance weighted (fixed effects) | 5 | -0.11 | 0.10 | 0.89 | 0.74 | 1.08 | 0.249 | 0.513 |  |
| Invasive mucinous ovarian cancer | Carnitine | Maximum likelihood | 5 | -0.11 | 0.10 | 0.89 | 0.74 | 1.08 | 0.249 |  |  |
| Invasive mucinous ovarian cancer | Carnitine | Simple median | 5 | -0.29 | 0.20 | 0.74 | 0.51 | 1.10 | 0.134 |  |  |
| Invasive mucinous ovarian cancer | Carnitine | Weighted median | 5 | -0.11 | 0.10 | 0.90 | 0.73 | 1.10 | 0.301 |  |  |
| Invasive mucinous ovarian cancer | Carnitine | MR Egger | 5 | 0.02 | 0.18 | 1.02 | 0.71 | 1.45 | 0.933 |  | 0.452 |
| Invasive mucinous ovarian cancer | Citrulline | Inverse variance weighted (fixed effects) | 4 | -0.17 | 0.25 | 0.84 | 0.52 | 1.36 | 0.487 | 0.692 |  |
| Invasive mucinous ovarian cancer | Citrulline | Maximum likelihood | 4 | -0.17 | 0.25 | 0.84 | 0.52 | 1.37 | 0.487 |  |  |
| Invasive mucinous ovarian cancer | Citrulline | Simple median | 4 | -0.08 | 0.29 | 0.92 | 0.53 | 1.61 | 0.777 |  |  |
| Invasive mucinous ovarian cancer | Citrulline | Weighted median | 4 | -0.09 | 0.29 | 0.92 | 0.52 | 1.63 | 0.772 |  |  |
| Invasive mucinous ovarian cancer | Citrulline | MR Egger | 4 | -0.78 | 1.62 | 0.46 | 0.02 | 11.01 | 0.677 |  | 0.739 |
| Invasive mucinous ovarian cancer | Creatinine | Inverse variance weighted (fixed effects) | 12 | -0.79 | 0.32 | 0.45 | 0.24 | 0.84 | 0.012 | 0.666 |  |
| Invasive mucinous ovarian cancer | Creatinine | Maximum likelihood | 12 | -0.80 | 0.32 | 0.45 | 0.24 | 0.84 | 0.013 |  |  |
| Invasive mucinous ovarian cancer | Creatinine | Simple median | 12 | -0.89 | 0.43 | 0.41 | 0.18 | 0.95 | 0.038 |  |  |
| Invasive mucinous ovarian cancer | Creatinine | Weighted median | 12 | -0.85 | 0.43 | 0.43 | 0.19 | 0.99 | 0.046 |  |  |
| Invasive mucinous ovarian cancer | Creatinine | MR Egger | 12 | -2.91 | 1.48 | 0.05 | 0.00 | 1.00 | 0.079 |  | 0.175 |
| Invasive mucinous ovarian cancer | Decanoylcarnitine | Inverse variance weighted (multiplicative random effects) | 2 | -0.11 | 0.31 | 0.90 | 0.49 | 1.64 | 0.723 | 0.047 |  |
| Invasive mucinous ovarian cancer | Decanoylcarnitine | Maximum likelihood | 2 | -0.11 | 0.15 | 0.90 | 0.66 | 1.21 | 0.481 |  |  |
| Invasive mucinous ovarian cancer | Decenoylcarnitine | Inverse variance weighted (multiplicative random effects) | 3 | -0.22 | 0.34 | 0.81 | 0.42 | 1.56 | 0.524 | 0.034 |  |
| Invasive mucinous ovarian cancer | Decenoylcarnitine | Maximum likelihood | 3 | -0.22 | 0.19 | 0.80 | 0.55 | 1.16 | 0.236 |  |  |
| Invasive mucinous ovarian cancer | Decenoylcarnitine | Simple median | 3 | -0.68 | 0.32 | 0.50 | 0.27 | 0.95 | 0.033 |  |  |
| Invasive mucinous ovarian cancer | Decenoylcarnitine | Weighted median | 3 | -0.23 | 0.20 | 0.80 | 0.54 | 1.18 | 0.261 |  |  |
| Invasive mucinous ovarian cancer | Decenoylcarnitine | MR Egger | 3 | 1.36 | 0.68 | 3.89 | 1.03 | 14.66 | 0.294 |  | 0.250 |
| Invasive mucinous ovarian cancer | Dodecanoylcarnitine | Wald ratio | 1 | -0.78 | 0.74 | 0.46 | 0.11 | 1.94 | 0.288 | NA | NA |
| Invasive mucinous ovarian cancer | Glutamine | Inverse variance weighted (fixed effects) | 5 | 0.31 | 0.35 | 1.37 | 0.69 | 2.70 | 0.367 | 0.414 |  |
| Invasive mucinous ovarian cancer | Glutamine | Maximum likelihood | 5 | 0.32 | 0.35 | 1.37 | 0.69 | 2.71 | 0.365 |  |  |
| Invasive mucinous ovarian cancer | Glutamine | Simple median | 5 | 0.21 | 0.48 | 1.24 | 0.48 | 3.19 | 0.661 |  |  |
| Invasive mucinous ovarian cancer | Glutamine | Weighted median | 5 | 0.27 | 0.43 | 1.31 | 0.56 | 3.08 | 0.529 |  |  |
| Invasive mucinous ovarian cancer | Glutamine | MR Egger | 5 | 0.98 | 1.66 | 2.67 | 0.10 | 68.77 | 0.595 |  | 0.706 |
| Invasive mucinous ovarian cancer | Glycine | Inverse variance weighted (fixed effects) | 12 | -0.13 | 0.09 | 0.88 | 0.74 | 1.05 | 0.156 | 0.602 |  |
| Invasive mucinous ovarian cancer | Glycine | Maximum likelihood | 12 | -0.13 | 0.09 | 0.88 | 0.74 | 1.05 | 0.155 |  |  |
| Invasive mucinous ovarian cancer | Glycine | Simple median | 12 | -0.42 | 0.26 | 0.66 | 0.40 | 1.09 | 0.102 |  |  |
| Invasive mucinous ovarian cancer | Glycine | Weighted median | 12 | -0.09 | 0.10 | 0.92 | 0.75 | 1.11 | 0.369 |  |  |
| Invasive mucinous ovarian cancer | Glycine | MR Egger | 12 | -0.08 | 0.11 | 0.92 | 0.74 | 1.15 | 0.480 |  | 0.538 |
| Invasive mucinous ovarian cancer | Hexadecanoylcarnitine | Inverse variance weighted (fixed effects) | 2 | -0.32 | 0.29 | 0.72 | 0.41 | 1.27 | 0.259 | 0.856 |  |
| Invasive mucinous ovarian cancer | Hexadecanoylcarnitine | Maximum likelihood | 2 | -0.32 | 0.29 | 0.72 | 0.41 | 1.27 | 0.261 |  |  |
| Invasive mucinous ovarian cancer | Hexadecenoylcarnitine | Wald ratio | 1 | -0.73 | 0.44 | 0.48 | 0.20 | 1.15 | 0.102 | NA | NA |
| Invasive mucinous ovarian cancer | Hexanoylcarnitine | Inverse variance weighted (fixed effects) | 3 | -0.10 | 0.13 | 0.90 | 0.69 | 1.18 | 0.453 | 0.116 |  |
| Invasive mucinous ovarian cancer | Hexanoylcarnitine | Maximum likelihood | 3 | -0.10 | 0.14 | 0.90 | 0.69 | 1.18 | 0.451 |  |  |
| Invasive mucinous ovarian cancer | Hexanoylcarnitine | Simple median | 3 | -0.18 | 0.23 | 0.84 | 0.53 | 1.31 | 0.439 |  |  |
| Invasive mucinous ovarian cancer | Hexanoylcarnitine | Weighted median | 3 | -0.03 | 0.15 | 0.97 | 0.73 | 1.29 | 0.824 |  |  |
| Invasive mucinous ovarian cancer | Hexanoylcarnitine | MR Egger | 3 | 0.14 | 0.19 | 1.16 | 0.80 | 1.67 | 0.585 |  | 0.317 |
| Invasive mucinous ovarian cancer | Hexose | Wald ratio | 1 | 0.92 | 0.45 | 2.51 | 1.05 | 6.02 | 0.039 | NA | NA |
| Invasive mucinous ovarian cancer | Histidine | Inverse variance weighted (fixed effects) | 7 | 0.26 | 0.29 | 1.30 | 0.74 | 2.28 | 0.365 | 0.062 |  |
| Invasive mucinous ovarian cancer | Histidine | Maximum likelihood | 7 | 0.27 | 0.29 | 1.31 | 0.74 | 2.31 | 0.360 |  |  |
| Invasive mucinous ovarian cancer | Histidine | Simple median | 7 | 0.67 | 0.41 | 1.96 | 0.87 | 4.41 | 0.103 |  |  |
| Invasive mucinous ovarian cancer | Histidine | Weighted median | 7 | 0.68 | 0.38 | 1.98 | 0.94 | 4.16 | 0.071 |  |  |
| Invasive mucinous ovarian cancer | Histidine | MR Egger | 7 | 1.26 | 2.42 | 3.51 | 0.03 | 405.90 | 0.626 |  | 0.693 |
| Invasive mucinous ovarian cancer | Isoleucine | Inverse variance weighted (fixed effects) | 3 | 0.52 | 0.59 | 1.68 | 0.53 | 5.33 | 0.382 | 0.932 |  |
| Invasive mucinous ovarian cancer | Isoleucine | Maximum likelihood | 3 | 0.52 | 0.59 | 1.68 | 0.53 | 5.35 | 0.382 |  |  |
| Invasive mucinous ovarian cancer | Isoleucine | Simple median | 3 | 0.45 | 0.71 | 1.57 | 0.39 | 6.29 | 0.526 |  |  |
| Invasive mucinous ovarian cancer | Isoleucine | Weighted median | 3 | 0.49 | 0.65 | 1.63 | 0.45 | 5.86 | 0.454 |  |  |
| Invasive mucinous ovarian cancer | Isoleucine | MR Egger | 3 | 1.49 | 2.67 | 4.43 | 0.02 | 829.08 | 0.676 |  | 0.773 |
| Invasive mucinous ovarian cancer | Kynurenine | Inverse variance weighted (fixed effects) | 3 | 0.07 | 0.19 | 1.08 | 0.74 | 1.55 | 0.699 | 0.457 |  |
| Invasive mucinous ovarian cancer | Kynurenine | Maximum likelihood | 3 | 0.07 | 0.19 | 1.08 | 0.74 | 1.55 | 0.698 |  |  |
| Invasive mucinous ovarian cancer | Kynurenine | Simple median | 3 | 0.28 | 0.31 | 1.33 | 0.73 | 2.42 | 0.356 |  |  |
| Invasive mucinous ovarian cancer | Kynurenine | Weighted median | 3 | 0.06 | 0.21 | 1.06 | 0.71 | 1.59 | 0.776 |  |  |
| Invasive mucinous ovarian cancer | Kynurenine | MR Egger | 3 | -0.47 | 0.55 | 0.62 | 0.21 | 1.82 | 0.547 |  | 0.482 |
| Invasive mucinous ovarian cancer | Leucine | Inverse variance weighted (fixed effects) | 4 | 0.54 | 0.53 | 1.72 | 0.61 | 4.85 | 0.303 | 0.746 |  |
| Invasive mucinous ovarian cancer | Leucine | Maximum likelihood | 4 | 0.55 | 0.53 | 1.73 | 0.61 | 4.89 | 0.303 |  |  |
| Invasive mucinous ovarian cancer | Leucine | Simple median | 4 | 0.55 | 0.62 | 1.74 | 0.52 | 5.82 | 0.371 |  |  |
| Invasive mucinous ovarian cancer | Leucine | Weighted median | 4 | 0.53 | 0.59 | 1.70 | 0.53 | 5.45 | 0.374 |  |  |
| Invasive mucinous ovarian cancer | Leucine | MR Egger | 4 | 0.74 | 1.71 | 2.10 | 0.07 | 59.66 | 0.706 |  | 0.914 |
| Invasive mucinous ovarian cancer | Lysine | Inverse variance weighted (fixed effects) | 6 | 0.20 | 0.16 | 1.22 | 0.89 | 1.67 | 0.210 | 0.557 |  |
| Invasive mucinous ovarian cancer | Lysine | Maximum likelihood | 6 | 0.20 | 0.16 | 1.22 | 0.89 | 1.67 | 0.210 |  |  |
| Invasive mucinous ovarian cancer | Lysine | Simple median | 6 | 0.33 | 0.23 | 1.39 | 0.88 | 2.19 | 0.159 |  |  |
| Invasive mucinous ovarian cancer | Lysine | Weighted median | 6 | 0.15 | 0.19 | 1.16 | 0.80 | 1.69 | 0.436 |  |  |
| Invasive mucinous ovarian cancer | Lysine | MR Egger | 6 | -0.31 | 0.37 | 0.73 | 0.35 | 1.52 | 0.454 |  | 0.206 |
| Invasive mucinous ovarian cancer | Methionine | Wald ratio | 1 | -1.47 | 0.69 | 0.23 | 0.06 | 0.89 | 0.033 | NA | NA |
| Invasive mucinous ovarian cancer | Methioninesulfoxide | Wald ratio | 1 | -0.11 | 0.56 | 0.90 | 0.30 | 2.68 | 0.848 | NA | NA |
| Invasive mucinous ovarian cancer | Methylglutarylcarnitine | Inverse variance weighted (multiplicative random effects) | 4 | -0.28 | 0.32 | 0.76 | 0.41 | 1.41 | 0.383 | 0.013 |  |
| Invasive mucinous ovarian cancer | Methylglutarylcarnitine | Maximum likelihood | 4 | -0.28 | 0.17 | 0.76 | 0.54 | 1.05 | 0.097 |  |  |
| Invasive mucinous ovarian cancer | Methylglutarylcarnitine | Simple median | 4 | -0.30 | 0.27 | 0.74 | 0.44 | 1.26 | 0.272 |  |  |
| Invasive mucinous ovarian cancer | Methylglutarylcarnitine | Weighted median | 4 | -0.27 | 0.19 | 0.76 | 0.52 | 1.12 | 0.167 |  |  |
| Invasive mucinous ovarian cancer | Methylglutarylcarnitine | MR Egger | 4 | -0.73 | 1.07 | 0.48 | 0.06 | 3.90 | 0.563 |  | 0.693 |
| Invasive mucinous ovarian cancer | Nonaylcarnitine | Inverse variance weighted (fixed effects) | 2 | -0.05 | 0.15 | 0.95 | 0.70 | 1.27 | 0.718 | 0.819 |  |
| Invasive mucinous ovarian cancer | Nonaylcarnitine | Maximum likelihood | 2 | -0.05 | 0.15 | 0.95 | 0.70 | 1.27 | 0.718 |  |  |
| Invasive mucinous ovarian cancer | Octadecandienylcarnitine | Inverse variance weighted (fixed effects) | 4 | -0.27 | 0.15 | 0.76 | 0.57 | 1.03 | 0.073 | 0.751 |  |
| Invasive mucinous ovarian cancer | Octadecandienylcarnitine | Maximum likelihood | 4 | -0.27 | 0.15 | 0.76 | 0.57 | 1.03 | 0.074 |  |  |
| Invasive mucinous ovarian cancer | Octadecandienylcarnitine | Simple median | 4 | -0.31 | 0.22 | 0.73 | 0.48 | 1.13 | 0.155 |  |  |
| Invasive mucinous ovarian cancer | Octadecandienylcarnitine | Weighted median | 4 | -0.30 | 0.16 | 0.74 | 0.54 | 1.01 | 0.055 |  |  |
| Invasive mucinous ovarian cancer | Octadecandienylcarnitine | MR Egger | 4 | -0.29 | 0.36 | 0.75 | 0.37 | 1.53 | 0.512 |  | 0.966 |
| Invasive mucinous ovarian cancer | Octadecanoylcarnitine | Inverse variance weighted (fixed effects) | 5 | 0.11 | 0.23 | 1.12 | 0.71 | 1.77 | 0.628 | 0.223 |  |
| Invasive mucinous ovarian cancer | Octadecanoylcarnitine | Maximum likelihood | 5 | 0.12 | 0.23 | 1.12 | 0.71 | 1.78 | 0.623 |  |  |
| Invasive mucinous ovarian cancer | Octadecanoylcarnitine | Simple median | 5 | -0.22 | 0.34 | 0.81 | 0.41 | 1.57 | 0.527 |  |  |
| Invasive mucinous ovarian cancer | Octadecanoylcarnitine | Weighted median | 5 | -0.24 | 0.30 | 0.79 | 0.44 | 1.43 | 0.432 |  |  |
| Invasive mucinous ovarian cancer | Octadecanoylcarnitine | MR Egger | 5 | -0.77 | 1.78 | 0.47 | 0.01 | 15.37 | 0.697 |  | 0.652 |
| Invasive mucinous ovarian cancer | Octadecenoylcarnitine | Inverse variance weighted (fixed effects) | 2 | -0.40 | 0.21 | 0.67 | 0.44 | 1.01 | 0.053 | 0.963 |  |
| Invasive mucinous ovarian cancer | Octadecenoylcarnitine | Maximum likelihood | 2 | -0.40 | 0.21 | 0.67 | 0.44 | 1.01 | 0.056 |  |  |
| Invasive mucinous ovarian cancer | Octanoylcarnitine | Inverse variance weighted (fixed effects) | 5 | -0.12 | 0.12 | 0.88 | 0.70 | 1.12 | 0.310 | 0.144 |  |
| Invasive mucinous ovarian cancer | Octanoylcarnitine | Maximum likelihood | 5 | -0.12 | 0.12 | 0.88 | 0.70 | 1.12 | 0.309 |  |  |
| Invasive mucinous ovarian cancer | Octanoylcarnitine | Simple median | 5 | -0.46 | 0.27 | 0.63 | 0.37 | 1.08 | 0.094 |  |  |
| Invasive mucinous ovarian cancer | Octanoylcarnitine | Weighted median | 5 | -0.15 | 0.15 | 0.86 | 0.63 | 1.16 | 0.315 |  |  |
| Invasive mucinous ovarian cancer | Octanoylcarnitine | MR Egger | 5 | 0.22 | 0.32 | 1.24 | 0.67 | 2.30 | 0.542 |  | 0.309 |
| Invasive mucinous ovarian cancer | Ornithine | Inverse variance weighted (fixed effects) | 6 | -0.06 | 0.24 | 0.94 | 0.58 | 1.53 | 0.816 | 0.769 |  |
| Invasive mucinous ovarian cancer | Ornithine | Maximum likelihood | 6 | -0.06 | 0.25 | 0.94 | 0.58 | 1.53 | 0.814 |  |  |
| Invasive mucinous ovarian cancer | Ornithine | Simple median | 6 | -0.09 | 0.32 | 0.91 | 0.49 | 1.70 | 0.768 |  |  |
| Invasive mucinous ovarian cancer | Ornithine | Weighted median | 6 | -0.01 | 0.30 | 0.99 | 0.55 | 1.78 | 0.967 |  |  |
| Invasive mucinous ovarian cancer | Ornithine | MR Egger | 6 | 0.67 | 0.61 | 1.96 | 0.59 | 6.49 | 0.332 |  | 0.262 |
| Invasive mucinous ovarian cancer | Phenylalanine | Inverse variance weighted (fixed effects) | 6 | 0.33 | 0.32 | 1.39 | 0.74 | 2.62 | 0.305 | 0.355 |  |
| Invasive mucinous ovarian cancer | Phenylalanine | Maximum likelihood | 6 | 0.33 | 0.32 | 1.40 | 0.74 | 2.64 | 0.303 |  |  |
| Invasive mucinous ovarian cancer | Phenylalanine | Simple median | 6 | 0.39 | 0.44 | 1.48 | 0.62 | 3.53 | 0.374 |  |  |
| Invasive mucinous ovarian cancer | Phenylalanine | Weighted median | 6 | 0.53 | 0.41 | 1.69 | 0.76 | 3.77 | 0.199 |  |  |
| Invasive mucinous ovarian cancer | Phenylalanine | MR Egger | 6 | 0.06 | 1.50 | 1.06 | 0.06 | 20.18 | 0.970 |  | 0.861 |
| Invasive mucinous ovarian cancer | Proline | Inverse variance weighted (fixed effects) | 2 | 0.07 | 0.15 | 1.07 | 0.80 | 1.44 | 0.628 | 0.379 |  |
| Invasive mucinous ovarian cancer | Proline | Maximum likelihood | 2 | 0.07 | 0.15 | 1.07 | 0.80 | 1.44 | 0.628 |  |  |
| Invasive mucinous ovarian cancer | Propionylcarnitine | Inverse variance weighted (fixed effects) | 4 | -0.20 | 0.15 | 0.82 | 0.62 | 1.09 | 0.170 | 0.693 |  |
| Invasive mucinous ovarian cancer | Propionylcarnitine | Maximum likelihood | 4 | -0.20 | 0.15 | 0.82 | 0.62 | 1.09 | 0.170 |  |  |
| Invasive mucinous ovarian cancer | Propionylcarnitine | Simple median | 4 | -0.39 | 0.24 | 0.68 | 0.42 | 1.10 | 0.112 |  |  |
| Invasive mucinous ovarian cancer | Propionylcarnitine | Weighted median | 4 | -0.17 | 0.15 | 0.85 | 0.63 | 1.14 | 0.278 |  |  |
| Invasive mucinous ovarian cancer | Propionylcarnitine | MR Egger | 4 | -0.04 | 0.34 | 0.97 | 0.50 | 1.88 | 0.927 |  | 0.647 |
| Invasive mucinous ovarian cancer | Sarcosine | Inverse variance weighted (fixed effects) | 2 | -0.41 | 0.34 | 0.66 | 0.34 | 1.30 | 0.228 | 0.597 |  |
| Invasive mucinous ovarian cancer | Sarcosine | Maximum likelihood | 2 | -0.41 | 0.34 | 0.66 | 0.34 | 1.30 | 0.229 |  |  |
| Invasive mucinous ovarian cancer | Serine | Inverse variance weighted (fixed effects) | 4 | -0.03 | 0.14 | 0.97 | 0.74 | 1.29 | 0.857 | 0.486 |  |
| Invasive mucinous ovarian cancer | Serine | Maximum likelihood | 4 | -0.03 | 0.14 | 0.97 | 0.74 | 1.29 | 0.857 |  |  |
| Invasive mucinous ovarian cancer | Serine | Simple median | 4 | -0.02 | 0.17 | 0.98 | 0.69 | 1.37 | 0.887 |  |  |
| Invasive mucinous ovarian cancer | Serine | Weighted median | 4 | -0.01 | 0.17 | 0.99 | 0.71 | 1.39 | 0.961 |  |  |
| Invasive mucinous ovarian cancer | Serine | MR Egger | 4 | -0.10 | 0.60 | 0.90 | 0.28 | 2.92 | 0.879 |  | 0.906 |
| Invasive mucinous ovarian cancer | Spermidine | Wald ratio | 1 | -0.31 | 0.24 | 0.74 | 0.46 | 1.18 | 0.204 | NA | NA |
| Invasive mucinous ovarian cancer | Symmetric dimethylarginine | Wald ratio | 1 | 0.17 | 0.34 | 1.19 | 0.61 | 2.32 | 0.609 | NA | NA |
| Invasive mucinous ovarian cancer | Taurine | Wald ratio | 1 | -0.24 | 0.29 | 0.79 | 0.44 | 1.41 | 0.422 | NA | NA |
| Invasive mucinous ovarian cancer | Tetradecanoylcarnitine | Inverse variance weighted (fixed effects) | 3 | -0.47 | 0.26 | 0.62 | 0.37 | 1.04 | 0.069 | 0.804 |  |
| Invasive mucinous ovarian cancer | Tetradecanoylcarnitine | Maximum likelihood | 3 | -0.47 | 0.26 | 0.62 | 0.37 | 1.04 | 0.071 |  |  |
| Invasive mucinous ovarian cancer | Tetradecanoylcarnitine | Simple median | 3 | -0.35 | 0.33 | 0.71 | 0.37 | 1.35 | 0.290 |  |  |
| Invasive mucinous ovarian cancer | Tetradecanoylcarnitine | Weighted median | 3 | -0.43 | 0.28 | 0.65 | 0.37 | 1.13 | 0.125 |  |  |
| Invasive mucinous ovarian cancer | Tetradecanoylcarnitine | MR Egger | 3 | -0.89 | 1.70 | 0.41 | 0.01 | 11.40 | 0.692 |  | 0.844 |
| Invasive mucinous ovarian cancer | Tetradecenoylcarnitine | Inverse variance weighted (fixed effects) | 2 | -0.93 | 0.44 | 0.39 | 0.17 | 0.93 | 0.034 | 0.502 |  |
| Invasive mucinous ovarian cancer | Tetradecenoylcarnitine | Maximum likelihood | 2 | -0.93 | 0.45 | 0.39 | 0.16 | 0.95 | 0.038 |  |  |
| Invasive mucinous ovarian cancer | Threonine | Inverse variance weighted (multiplicative random effects) | 3 | -0.14 | 0.50 | 0.87 | 0.33 | 2.31 | 0.780 | 0.024 |  |
| Invasive mucinous ovarian cancer | Threonine | Maximum likelihood | 3 | -0.14 | 0.26 | 0.87 | 0.52 | 1.45 | 0.585 |  |  |
| Invasive mucinous ovarian cancer | Threonine | Simple median | 3 | 0.12 | 0.34 | 1.12 | 0.58 | 2.18 | 0.733 |  |  |
| Invasive mucinous ovarian cancer | Threonine | Weighted median | 3 | 0.12 | 0.31 | 1.13 | 0.61 | 2.09 | 0.701 |  |  |
| Invasive mucinous ovarian cancer | Threonine | MR Egger | 3 | 0.55 | 3.02 | 1.73 | 0.00 | 638.74 | 0.885 |  | 0.853 |
| Invasive mucinous ovarian cancer | trans-Hydroxyproline | Wald ratio | 1 | 0.07 | 0.50 | 1.07 | 0.40 | 2.88 | 0.888 | NA | NA |
| Invasive mucinous ovarian cancer | Tryptophan | Inverse variance weighted (multiplicative random effects) | 2 | -0.26 | 0.67 | 0.77 | 0.21 | 2.89 | 0.699 | 0.028 |  |
| Invasive mucinous ovarian cancer | Tryptophan | Maximum likelihood | 2 | -0.27 | 0.31 | 0.77 | 0.42 | 1.41 | 0.392 |  |  |
| Invasive mucinous ovarian cancer | Tyrosine | Inverse variance weighted (fixed effects) | 2 | -0.45 | 0.30 | 0.64 | 0.36 | 1.14 | 0.130 | 0.493 |  |
| Invasive mucinous ovarian cancer | Tyrosine | Maximum likelihood | 2 | -0.45 | 0.30 | 0.64 | 0.36 | 1.14 | 0.130 |  |  |
| Invasive mucinous ovarian cancer | Valerylcarnitine | Inverse variance weighted (fixed effects) | 3 | -0.22 | 0.17 | 0.80 | 0.57 | 1.13 | 0.210 | 0.990 |  |
| Invasive mucinous ovarian cancer | Valerylcarnitine | Maximum likelihood | 3 | -0.22 | 0.17 | 0.80 | 0.57 | 1.13 | 0.211 |  |  |
| Invasive mucinous ovarian cancer | Valerylcarnitine | Simple median | 3 | -0.21 | 0.22 | 0.81 | 0.53 | 1.25 | 0.349 |  |  |
| Invasive mucinous ovarian cancer | Valerylcarnitine | Weighted median | 3 | -0.22 | 0.19 | 0.80 | 0.55 | 1.16 | 0.245 |  |  |
| Invasive mucinous ovarian cancer | Valerylcarnitine | MR Egger | 3 | -0.31 | 0.66 | 0.73 | 0.20 | 2.67 | 0.721 |  | 0.909 |
| Invasive mucinous ovarian cancer | Valine | Inverse variance weighted (fixed effects) | 4 | 0.27 | 0.37 | 1.31 | 0.64 | 2.69 | 0.463 | 0.905 |  |
| Invasive mucinous ovarian cancer | Valine | Maximum likelihood | 4 | 0.27 | 0.37 | 1.31 | 0.64 | 2.70 | 0.463 |  |  |
| Invasive mucinous ovarian cancer | Valine | Simple median | 4 | 0.41 | 0.46 | 1.51 | 0.62 | 3.68 | 0.367 |  |  |
| Invasive mucinous ovarian cancer | Valine | Weighted median | 4 | 0.24 | 0.41 | 1.27 | 0.57 | 2.84 | 0.558 |  |  |
| Invasive mucinous ovarian cancer | Valine | MR Egger | 4 | 1.03 | 1.85 | 2.79 | 0.07 | 104.20 | 0.634 |  | 0.717 |

| **Supplementary Table 7.** The association between exposure and Clear cell ovarian cancer. | | | | | | | | | | | |
| --- | --- | --- | --- | --- | --- | --- | --- | --- | --- | --- | --- |
| Outcome | Exposure | Method | No. | beta | se | OR | LCI | UCI | *P*-value | *P* for heterogenity | *P* for pleiotropy |
| Clear cell ovarian cancer | Acetylcarnitine | Inverse variance weighted (fixed effects) | 3 | -0.09 | 0.15 | 0.92 | 0.68 | 1.24 | 0.572 | 0.178 |  |
| Clear cell ovarian cancer | Acetylcarnitine | Maximum likelihood | 3 | -0.09 | 0.15 | 0.92 | 0.68 | 1.24 | 0.571 |  |  |
| Clear cell ovarian cancer | Acetylcarnitine | Simple median | 3 | 0.12 | 0.29 | 1.13 | 0.64 | 1.99 | 0.674 |  |  |
| Clear cell ovarian cancer | Acetylcarnitine | Weighted median | 3 | -0.13 | 0.15 | 0.88 | 0.65 | 1.18 | 0.398 |  |  |
| Clear cell ovarian cancer | Acetylcarnitine | MR Egger | 3 | -0.24 | 0.70 | 0.78 | 0.20 | 3.10 | 0.787 |  | 0.848 |
| Clear cell ovarian cancer | Acetylornithine | Inverse variance weighted (fixed effects) | 2 | -0.01 | 0.06 | 0.99 | 0.88 | 1.11 | 0.879 | 0.326 |  |
| Clear cell ovarian cancer | Acetylornithine | Maximum likelihood | 2 | -0.01 | 0.06 | 0.99 | 0.88 | 1.11 | 0.879 |  |  |
| Clear cell ovarian cancer | Alanine | Inverse variance weighted (fixed effects) | 9 | -0.11 | 0.33 | 0.89 | 0.47 | 1.70 | 0.729 | 0.941 |  |
| Clear cell ovarian cancer | Alanine | Maximum likelihood | 9 | -0.11 | 0.33 | 0.89 | 0.47 | 1.71 | 0.729 |  |  |
| Clear cell ovarian cancer | Alanine | Simple median | 9 | -0.15 | 0.44 | 0.86 | 0.36 | 2.01 | 0.723 |  |  |
| Clear cell ovarian cancer | Alanine | Weighted median | 9 | -0.16 | 0.43 | 0.85 | 0.37 | 1.99 | 0.715 |  |  |
| Clear cell ovarian cancer | Alanine | MR Egger | 9 | 0.11 | 1.31 | 1.11 | 0.09 | 14.41 | 0.936 |  | 0.865 |
| Clear cell ovarian cancer | alpha-Aminoadipic acid | Inverse variance weighted (fixed effects) | 2 | -0.03 | 0.35 | 0.97 | 0.49 | 1.95 | 0.935 | 0.274 |  |
| Clear cell ovarian cancer | alpha-Aminoadipic acid | Maximum likelihood | 2 | -0.03 | 0.36 | 0.97 | 0.48 | 1.95 | 0.935 |  |  |
| Clear cell ovarian cancer | Arginine | Inverse variance weighted (fixed effects) | 7 | -0.29 | 0.17 | 0.75 | 0.53 | 1.05 | 0.095 | 0.636 |  |
| Clear cell ovarian cancer | Arginine | Maximum likelihood | 7 | -0.29 | 0.18 | 0.75 | 0.53 | 1.05 | 0.094 |  |  |
| Clear cell ovarian cancer | Arginine | Simple median | 7 | -0.15 | 0.26 | 0.86 | 0.52 | 1.44 | 0.576 |  |  |
| Clear cell ovarian cancer | Arginine | Weighted median | 7 | -0.34 | 0.21 | 0.71 | 0.47 | 1.08 | 0.108 |  |  |
| Clear cell ovarian cancer | Arginine | MR Egger | 7 | -0.59 | 0.29 | 0.55 | 0.31 | 0.98 | 0.098 |  | 0.254 |
| Clear cell ovarian cancer | Asparagine | Inverse variance weighted (fixed effects) | 4 | 0.00 | 0.13 | 1.00 | 0.78 | 1.28 | 0.976 | 0.904 |  |
| Clear cell ovarian cancer | Asparagine | Maximum likelihood | 4 | 0.00 | 0.13 | 1.00 | 0.78 | 1.28 | 0.976 |  |  |
| Clear cell ovarian cancer | Asparagine | Simple median | 4 | -0.04 | 0.25 | 0.96 | 0.58 | 1.58 | 0.870 |  |  |
| Clear cell ovarian cancer | Asparagine | Weighted median | 4 | 0.02 | 0.13 | 1.02 | 0.79 | 1.30 | 0.902 |  |  |
| Clear cell ovarian cancer | Asparagine | MR Egger | 4 | 0.05 | 0.17 | 1.06 | 0.75 | 1.49 | 0.784 |  | 0.716 |
| Clear cell ovarian cancer | Aspartate | Inverse variance weighted (fixed effects) | 2 | -0.32 | 0.24 | 0.73 | 0.46 | 1.16 | 0.178 | 0.410 |  |
| Clear cell ovarian cancer | Aspartate | Maximum likelihood | 2 | -0.32 | 0.24 | 0.73 | 0.46 | 1.16 | 0.179 |  |  |
| Clear cell ovarian cancer | Butyrylcarnitine | Inverse variance weighted (fixed effects) | 2 | -0.48 | 0.22 | 0.62 | 0.40 | 0.95 | 0.029 | 0.116 |  |
| Clear cell ovarian cancer | Butyrylcarnitine | Maximum likelihood | 2 | -0.48 | 0.22 | 0.62 | 0.40 | 0.95 | 0.030 |  |  |
| Clear cell ovarian cancer | Carnitine | Inverse variance weighted (fixed effects) | 5 | -0.07 | 0.10 | 0.93 | 0.77 | 1.13 | 0.488 | 0.358 |  |
| Clear cell ovarian cancer | Carnitine | Maximum likelihood | 5 | -0.07 | 0.10 | 0.93 | 0.77 | 1.13 | 0.487 |  |  |
| Clear cell ovarian cancer | Carnitine | Simple median | 5 | 0.44 | 0.30 | 1.55 | 0.85 | 2.80 | 0.151 |  |  |
| Clear cell ovarian cancer | Carnitine | Weighted median | 5 | -0.14 | 0.10 | 0.87 | 0.72 | 1.06 | 0.180 |  |  |
| Clear cell ovarian cancer | Carnitine | MR Egger | 5 | -0.19 | 0.21 | 0.83 | 0.55 | 1.24 | 0.428 |  | 0.540 |
| Clear cell ovarian cancer | Citrulline | Inverse variance weighted (fixed effects) | 4 | -0.13 | 0.25 | 0.88 | 0.54 | 1.43 | 0.598 | 0.632 |  |
| Clear cell ovarian cancer | Citrulline | Maximum likelihood | 4 | -0.13 | 0.25 | 0.88 | 0.54 | 1.43 | 0.597 |  |  |
| Clear cell ovarian cancer | Citrulline | Simple median | 4 | -0.09 | 0.28 | 0.92 | 0.53 | 1.60 | 0.763 |  |  |
| Clear cell ovarian cancer | Citrulline | Weighted median | 4 | -0.13 | 0.29 | 0.88 | 0.50 | 1.55 | 0.653 |  |  |
| Clear cell ovarian cancer | Citrulline | MR Egger | 4 | 0.31 | 1.61 | 1.36 | 0.06 | 31.96 | 0.866 |  | 0.809 |
| Clear cell ovarian cancer | Creatinine | Inverse variance weighted (fixed effects) | 12 | 0.61 | 0.32 | 1.84 | 0.99 | 3.42 | 0.055 | 0.083 |  |
| Clear cell ovarian cancer | Creatinine | Maximum likelihood | 12 | 0.63 | 0.32 | 1.88 | 1.00 | 3.53 | 0.051 |  |  |
| Clear cell ovarian cancer | Creatinine | Simple median | 12 | 0.08 | 0.46 | 1.08 | 0.44 | 2.67 | 0.860 |  |  |
| Clear cell ovarian cancer | Creatinine | Weighted median | 12 | 0.39 | 0.45 | 1.48 | 0.61 | 3.60 | 0.383 |  |  |
| Clear cell ovarian cancer | Creatinine | MR Egger | 12 | 0.68 | 2.00 | 1.97 | 0.04 | 98.79 | 0.741 |  | 0.972 |
| Clear cell ovarian cancer | Decanoylcarnitine | Inverse variance weighted (fixed effects) | 2 | -0.05 | 0.16 | 0.95 | 0.70 | 1.29 | 0.751 | 0.489 |  |
| Clear cell ovarian cancer | Decanoylcarnitine | Maximum likelihood | 2 | -0.05 | 0.16 | 0.95 | 0.70 | 1.29 | 0.751 |  |  |
| Clear cell ovarian cancer | Decenoylcarnitine | Inverse variance weighted (fixed effects) | 3 | -0.19 | 0.19 | 0.83 | 0.58 | 1.19 | 0.310 | 0.147 |  |
| Clear cell ovarian cancer | Decenoylcarnitine | Maximum likelihood | 3 | -0.19 | 0.19 | 0.83 | 0.57 | 1.19 | 0.307 |  |  |
| Clear cell ovarian cancer | Decenoylcarnitine | Simple median | 3 | -0.26 | 0.28 | 0.77 | 0.44 | 1.35 | 0.363 |  |  |
| Clear cell ovarian cancer | Decenoylcarnitine | Weighted median | 3 | -0.10 | 0.21 | 0.91 | 0.60 | 1.38 | 0.653 |  |  |
| Clear cell ovarian cancer | Decenoylcarnitine | MR Egger | 3 | 1.08 | 0.68 | 2.95 | 0.77 | 11.27 | 0.359 |  | 0.304 |
| Clear cell ovarian cancer | Dodecanoylcarnitine | Wald ratio | 1 | 0.40 | 0.75 | 1.50 | 0.35 | 6.46 | 0.589 | NA | NA |
| Clear cell ovarian cancer | Glutamine | Inverse variance weighted (fixed effects) | 5 | -0.02 | 0.35 | 0.98 | 0.49 | 1.96 | 0.960 | 0.091 |  |
| Clear cell ovarian cancer | Glutamine | Maximum likelihood | 5 | -0.02 | 0.36 | 0.98 | 0.49 | 1.97 | 0.960 |  |  |
| Clear cell ovarian cancer | Glutamine | Simple median | 5 | 0.13 | 0.53 | 1.14 | 0.40 | 3.23 | 0.803 |  |  |
| Clear cell ovarian cancer | Glutamine | Weighted median | 5 | -0.05 | 0.46 | 0.96 | 0.39 | 2.36 | 0.922 |  |  |
| Clear cell ovarian cancer | Glutamine | MR Egger | 5 | -3.34 | 1.53 | 0.04 | 0.00 | 0.71 | 0.117 |  | 0.112 |
| Clear cell ovarian cancer | Glycine | Inverse variance weighted (fixed effects) | 12 | 0.03 | 0.09 | 1.04 | 0.87 | 1.24 | 0.697 | 0.591 |  |
| Clear cell ovarian cancer | Glycine | Maximum likelihood | 12 | 0.04 | 0.09 | 1.04 | 0.87 | 1.24 | 0.693 |  |  |
| Clear cell ovarian cancer | Glycine | Simple median | 12 | -0.45 | 0.28 | 0.64 | 0.36 | 1.11 | 0.113 |  |  |
| Clear cell ovarian cancer | Glycine | Weighted median | 12 | 0.10 | 0.10 | 1.10 | 0.91 | 1.33 | 0.315 |  |  |
| Clear cell ovarian cancer | Glycine | MR Egger | 12 | 0.16 | 0.11 | 1.17 | 0.94 | 1.46 | 0.197 |  | 0.109 |
| Clear cell ovarian cancer | Hexadecanoylcarnitine | Inverse variance weighted (fixed effects) | 2 | -0.07 | 0.29 | 0.93 | 0.53 | 1.65 | 0.812 | 0.186 |  |
| Clear cell ovarian cancer | Hexadecanoylcarnitine | Maximum likelihood | 2 | -0.07 | 0.29 | 0.93 | 0.53 | 1.65 | 0.811 |  |  |
| Clear cell ovarian cancer | Hexadecenoylcarnitine | Wald ratio | 1 | 0.18 | 0.45 | 1.19 | 0.49 | 2.91 | 0.697 | NA | NA |
| Clear cell ovarian cancer | Hexanoylcarnitine | Inverse variance weighted (fixed effects) | 3 | -0.12 | 0.14 | 0.89 | 0.67 | 1.16 | 0.384 | 0.461 |  |
| Clear cell ovarian cancer | Hexanoylcarnitine | Maximum likelihood | 3 | -0.12 | 0.14 | 0.89 | 0.67 | 1.16 | 0.384 |  |  |
| Clear cell ovarian cancer | Hexanoylcarnitine | Simple median | 3 | -0.34 | 0.20 | 0.71 | 0.48 | 1.06 | 0.094 |  |  |
| Clear cell ovarian cancer | Hexanoylcarnitine | Weighted median | 3 | -0.15 | 0.15 | 0.86 | 0.64 | 1.16 | 0.322 |  |  |
| Clear cell ovarian cancer | Hexanoylcarnitine | MR Egger | 3 | 0.06 | 0.20 | 1.06 | 0.71 | 1.57 | 0.824 |  | 0.432 |
| Clear cell ovarian cancer | Hexose | Wald ratio | 1 | 0.68 | 0.45 | 1.98 | 0.82 | 4.77 | 0.129 | NA | NA |
| Clear cell ovarian cancer | Histidine | Inverse variance weighted (fixed effects) | 7 | -0.21 | 0.29 | 0.81 | 0.46 | 1.43 | 0.465 | 0.877 |  |
| Clear cell ovarian cancer | Histidine | Maximum likelihood | 7 | -0.21 | 0.29 | 0.81 | 0.45 | 1.43 | 0.464 |  |  |
| Clear cell ovarian cancer | Histidine | Simple median | 7 | -0.11 | 0.40 | 0.90 | 0.41 | 1.98 | 0.794 |  |  |
| Clear cell ovarian cancer | Histidine | Weighted median | 7 | -0.31 | 0.37 | 0.74 | 0.36 | 1.51 | 0.403 |  |  |
| Clear cell ovarian cancer | Histidine | MR Egger | 7 | 0.33 | 1.62 | 1.40 | 0.06 | 33.30 | 0.844 |  | 0.744 |
| Clear cell ovarian cancer | Isoleucine | Inverse variance weighted (fixed effects) | 3 | 0.10 | 0.60 | 1.11 | 0.34 | 3.56 | 0.866 | 0.144 |  |
| Clear cell ovarian cancer | Isoleucine | Maximum likelihood | 3 | 0.10 | 0.60 | 1.11 | 0.34 | 3.61 | 0.864 |  |  |
| Clear cell ovarian cancer | Isoleucine | Simple median | 3 | 0.40 | 0.77 | 1.49 | 0.33 | 6.81 | 0.605 |  |  |
| Clear cell ovarian cancer | Isoleucine | Weighted median | 3 | 0.50 | 0.74 | 1.64 | 0.39 | 6.95 | 0.500 |  |  |
| Clear cell ovarian cancer | Isoleucine | MR Egger | 3 | 4.85 | 2.69 | 127.88 | 0.65 | 25153.21 | 0.323 |  | 0.322 |
| Clear cell ovarian cancer | Kynurenine | Inverse variance weighted (fixed effects) | 3 | -0.09 | 0.19 | 0.91 | 0.63 | 1.33 | 0.637 | 0.561 |  |
| Clear cell ovarian cancer | Kynurenine | Maximum likelihood | 3 | -0.09 | 0.19 | 0.91 | 0.63 | 1.33 | 0.637 |  |  |
| Clear cell ovarian cancer | Kynurenine | Simple median | 3 | -0.16 | 0.28 | 0.85 | 0.49 | 1.47 | 0.560 |  |  |
| Clear cell ovarian cancer | Kynurenine | Weighted median | 3 | -0.04 | 0.21 | 0.96 | 0.64 | 1.44 | 0.837 |  |  |
| Clear cell ovarian cancer | Kynurenine | MR Egger | 3 | 0.29 | 0.55 | 1.34 | 0.45 | 3.97 | 0.692 |  | 0.597 |
| Clear cell ovarian cancer | Leucine | Inverse variance weighted (fixed effects) | 4 | 0.47 | 0.53 | 1.60 | 0.56 | 4.55 | 0.379 | 0.670 |  |
| Clear cell ovarian cancer | Leucine | Maximum likelihood | 4 | 0.47 | 0.54 | 1.60 | 0.56 | 4.59 | 0.378 |  |  |
| Clear cell ovarian cancer | Leucine | Simple median | 4 | 0.42 | 0.67 | 1.53 | 0.41 | 5.69 | 0.528 |  |  |
| Clear cell ovarian cancer | Leucine | Weighted median | 4 | 0.61 | 0.66 | 1.85 | 0.50 | 6.75 | 0.354 |  |  |
| Clear cell ovarian cancer | Leucine | MR Egger | 4 | 2.20 | 1.72 | 9.06 | 0.31 | 265.53 | 0.329 |  | 0.401 |
| Clear cell ovarian cancer | Lysine | Inverse variance weighted (fixed effects) | 6 | -0.04 | 0.16 | 0.96 | 0.70 | 1.31 | 0.787 | 0.742 |  |
| Clear cell ovarian cancer | Lysine | Maximum likelihood | 6 | -0.04 | 0.16 | 0.96 | 0.70 | 1.31 | 0.787 |  |  |
| Clear cell ovarian cancer | Lysine | Simple median | 6 | -0.04 | 0.22 | 0.96 | 0.62 | 1.48 | 0.843 |  |  |
| Clear cell ovarian cancer | Lysine | Weighted median | 6 | 0.03 | 0.20 | 1.03 | 0.70 | 1.52 | 0.889 |  |  |
| Clear cell ovarian cancer | Lysine | MR Egger | 6 | 0.37 | 0.38 | 1.45 | 0.69 | 3.03 | 0.378 |  | 0.288 |
| Clear cell ovarian cancer | Methionine | Wald ratio | 1 | 0.63 | 0.68 | 1.87 | 0.49 | 7.17 | 0.359 | NA | NA |
| Clear cell ovarian cancer | Methioninesulfoxide | Wald ratio | 1 | -1.27 | 0.57 | 0.28 | 0.09 | 0.85 | 0.025 | NA | NA |
| Clear cell ovarian cancer | Methylglutarylcarnitine | Inverse variance weighted (fixed effects) | 4 | 0.14 | 0.17 | 1.15 | 0.83 | 1.60 | 0.392 | 0.217 |  |
| Clear cell ovarian cancer | Methylglutarylcarnitine | Maximum likelihood | 4 | 0.14 | 0.17 | 1.15 | 0.83 | 1.60 | 0.391 |  |  |
| Clear cell ovarian cancer | Methylglutarylcarnitine | Simple median | 4 | 0.19 | 0.25 | 1.21 | 0.74 | 1.98 | 0.448 |  |  |
| Clear cell ovarian cancer | Methylglutarylcarnitine | Weighted median | 4 | 0.24 | 0.19 | 1.27 | 0.87 | 1.86 | 0.208 |  |  |
| Clear cell ovarian cancer | Methylglutarylcarnitine | MR Egger | 4 | 0.57 | 0.65 | 1.76 | 0.49 | 6.30 | 0.474 |  | 0.558 |
| Clear cell ovarian cancer | Nonaylcarnitine | Inverse variance weighted (fixed effects) | 2 | 0.14 | 0.15 | 1.15 | 0.85 | 1.56 | 0.355 | 0.868 |  |
| Clear cell ovarian cancer | Nonaylcarnitine | Maximum likelihood | 2 | 0.14 | 0.15 | 1.15 | 0.85 | 1.56 | 0.356 |  |  |
| Clear cell ovarian cancer | Octadecandienylcarnitine | Inverse variance weighted (fixed effects) | 4 | 0.10 | 0.15 | 1.11 | 0.82 | 1.50 | 0.508 | 0.294 |  |
| Clear cell ovarian cancer | Octadecandienylcarnitine | Maximum likelihood | 4 | 0.10 | 0.15 | 1.11 | 0.82 | 1.50 | 0.507 |  |  |
| Clear cell ovarian cancer | Octadecandienylcarnitine | Simple median | 4 | 0.14 | 0.19 | 1.15 | 0.79 | 1.68 | 0.458 |  |  |
| Clear cell ovarian cancer | Octadecandienylcarnitine | Weighted median | 4 | 0.10 | 0.17 | 1.10 | 0.79 | 1.53 | 0.557 |  |  |
| Clear cell ovarian cancer | Octadecandienylcarnitine | MR Egger | 4 | 0.22 | 0.49 | 1.24 | 0.47 | 3.27 | 0.702 |  | 0.820 |
| Clear cell ovarian cancer | Octadecanoylcarnitine | Inverse variance weighted (fixed effects) | 5 | -0.08 | 0.24 | 0.92 | 0.58 | 1.46 | 0.723 | 0.330 |  |
| Clear cell ovarian cancer | Octadecanoylcarnitine | Maximum likelihood | 5 | -0.08 | 0.24 | 0.92 | 0.58 | 1.46 | 0.721 |  |  |
| Clear cell ovarian cancer | Octadecanoylcarnitine | Simple median | 5 | 0.05 | 0.32 | 1.05 | 0.56 | 1.96 | 0.877 |  |  |
| Clear cell ovarian cancer | Octadecanoylcarnitine | Weighted median | 5 | 0.06 | 0.31 | 1.07 | 0.58 | 1.97 | 0.838 |  |  |
| Clear cell ovarian cancer | Octadecanoylcarnitine | MR Egger | 5 | 0.64 | 1.64 | 1.91 | 0.08 | 47.80 | 0.721 |  | 0.683 |
| Clear cell ovarian cancer | Octadecenoylcarnitine | Inverse variance weighted (fixed effects) | 2 | 0.14 | 0.21 | 1.15 | 0.76 | 1.74 | 0.522 | 0.797 |  |
| Clear cell ovarian cancer | Octadecenoylcarnitine | Maximum likelihood | 2 | 0.14 | 0.21 | 1.15 | 0.76 | 1.74 | 0.522 |  |  |
| Clear cell ovarian cancer | Octanoylcarnitine | Inverse variance weighted (fixed effects) | 5 | -0.08 | 0.12 | 0.92 | 0.73 | 1.17 | 0.518 | 0.160 |  |
| Clear cell ovarian cancer | Octanoylcarnitine | Maximum likelihood | 5 | -0.08 | 0.12 | 0.92 | 0.73 | 1.18 | 0.517 |  |  |
| Clear cell ovarian cancer | Octanoylcarnitine | Simple median | 5 | -0.17 | 0.20 | 0.84 | 0.56 | 1.25 | 0.396 |  |  |
| Clear cell ovarian cancer | Octanoylcarnitine | Weighted median | 5 | -0.05 | 0.14 | 0.96 | 0.73 | 1.25 | 0.740 |  |  |
| Clear cell ovarian cancer | Octanoylcarnitine | MR Egger | 5 | 0.42 | 0.26 | 1.52 | 0.91 | 2.52 | 0.205 |  | 0.117 |
| Clear cell ovarian cancer | Ornithine | Inverse variance weighted (fixed effects) | 6 | 0.07 | 0.25 | 1.07 | 0.66 | 1.73 | 0.785 | 0.456 |  |
| Clear cell ovarian cancer | Ornithine | Maximum likelihood | 6 | 0.07 | 0.25 | 1.07 | 0.66 | 1.74 | 0.783 |  |  |
| Clear cell ovarian cancer | Ornithine | Simple median | 6 | -0.09 | 0.33 | 0.92 | 0.48 | 1.76 | 0.797 |  |  |
| Clear cell ovarian cancer | Ornithine | Weighted median | 6 | -0.04 | 0.32 | 0.96 | 0.52 | 1.79 | 0.903 |  |  |
| Clear cell ovarian cancer | Ornithine | MR Egger | 6 | 0.90 | 0.60 | 2.47 | 0.76 | 7.98 | 0.205 |  | 0.200 |
| Clear cell ovarian cancer | Phenylalanine | Inverse variance weighted (fixed effects) | 6 | -0.23 | 0.33 | 0.79 | 0.42 | 1.50 | 0.475 | 0.835 |  |
| Clear cell ovarian cancer | Phenylalanine | Maximum likelihood | 6 | -0.23 | 0.33 | 0.79 | 0.42 | 1.50 | 0.474 |  |  |
| Clear cell ovarian cancer | Phenylalanine | Simple median | 6 | -0.25 | 0.41 | 0.78 | 0.35 | 1.75 | 0.550 |  |  |
| Clear cell ovarian cancer | Phenylalanine | Weighted median | 6 | -0.24 | 0.40 | 0.78 | 0.36 | 1.71 | 0.540 |  |  |
| Clear cell ovarian cancer | Phenylalanine | MR Egger | 6 | -0.04 | 1.30 | 0.96 | 0.08 | 12.13 | 0.974 |  | 0.888 |
| Clear cell ovarian cancer | Proline | Inverse variance weighted (fixed effects) | 2 | 0.22 | 0.15 | 1.24 | 0.93 | 1.65 | 0.139 | 0.810 |  |
| Clear cell ovarian cancer | Proline | Maximum likelihood | 2 | 0.22 | 0.15 | 1.24 | 0.93 | 1.65 | 0.140 |  |  |
| Clear cell ovarian cancer | Propionylcarnitine | Inverse variance weighted (fixed effects) | 4 | -0.11 | 0.15 | 0.90 | 0.67 | 1.19 | 0.451 | 0.394 |  |
| Clear cell ovarian cancer | Propionylcarnitine | Maximum likelihood | 4 | -0.11 | 0.15 | 0.90 | 0.67 | 1.19 | 0.450 |  |  |
| Clear cell ovarian cancer | Propionylcarnitine | Simple median | 4 | -0.09 | 0.22 | 0.92 | 0.59 | 1.41 | 0.690 |  |  |
| Clear cell ovarian cancer | Propionylcarnitine | Weighted median | 4 | -0.15 | 0.16 | 0.86 | 0.63 | 1.18 | 0.351 |  |  |
| Clear cell ovarian cancer | Propionylcarnitine | MR Egger | 4 | -0.08 | 0.42 | 0.92 | 0.41 | 2.11 | 0.870 |  | 0.940 |
| Clear cell ovarian cancer | Sarcosine | Inverse variance weighted (fixed effects) | 2 | -0.18 | 0.35 | 0.84 | 0.42 | 1.65 | 0.607 | 0.584 |  |
| Clear cell ovarian cancer | Sarcosine | Maximum likelihood | 2 | -0.18 | 0.35 | 0.84 | 0.42 | 1.65 | 0.607 |  |  |
| Clear cell ovarian cancer | Serine | Inverse variance weighted (fixed effects) | 4 | -0.03 | 0.14 | 0.97 | 0.74 | 1.29 | 0.852 | 0.389 |  |
| Clear cell ovarian cancer | Serine | Maximum likelihood | 4 | -0.03 | 0.14 | 0.97 | 0.74 | 1.29 | 0.852 |  |  |
| Clear cell ovarian cancer | Serine | Simple median | 4 | -0.10 | 0.20 | 0.91 | 0.62 | 1.33 | 0.618 |  |  |
| Clear cell ovarian cancer | Serine | Weighted median | 4 | -0.11 | 0.18 | 0.90 | 0.63 | 1.29 | 0.559 |  |  |
| Clear cell ovarian cancer | Serine | MR Egger | 4 | 0.01 | 0.67 | 1.01 | 0.27 | 3.78 | 0.985 |  | 0.956 |
| Clear cell ovarian cancer | Spermidine | Wald ratio | 1 | 0.06 | 0.24 | 1.06 | 0.66 | 1.71 | 0.796 | NA | NA |
| Clear cell ovarian cancer | Symmetric dimethylarginine | Wald ratio | 1 | 0.22 | 0.35 | 1.25 | 0.64 | 2.46 | 0.517 | NA | NA |
| Clear cell ovarian cancer | Taurine | Wald ratio | 1 | -0.07 | 0.29 | 0.93 | 0.52 | 1.66 | 0.812 | NA | NA |
| Clear cell ovarian cancer | Tetradecanoylcarnitine | Inverse variance weighted (fixed effects) | 3 | 0.01 | 0.26 | 1.01 | 0.60 | 1.70 | 0.959 | 0.393 |  |
| Clear cell ovarian cancer | Tetradecanoylcarnitine | Maximum likelihood | 3 | 0.01 | 0.26 | 1.01 | 0.60 | 1.70 | 0.959 |  |  |
| Clear cell ovarian cancer | Tetradecanoylcarnitine | Simple median | 3 | 0.17 | 0.32 | 1.18 | 0.63 | 2.21 | 0.596 |  |  |
| Clear cell ovarian cancer | Tetradecanoylcarnitine | Weighted median | 3 | 0.18 | 0.29 | 1.19 | 0.68 | 2.11 | 0.545 |  |  |
| Clear cell ovarian cancer | Tetradecanoylcarnitine | MR Egger | 3 | 2.32 | 1.70 | 10.13 | 0.36 | 286.25 | 0.404 |  | 0.402 |
| Clear cell ovarian cancer | Tetradecenoylcarnitine | Inverse variance weighted (fixed effects) | 2 | -0.05 | 0.44 | 0.95 | 0.40 | 2.27 | 0.910 | 0.368 |  |
| Clear cell ovarian cancer | Tetradecenoylcarnitine | Maximum likelihood | 2 | -0.05 | 0.45 | 0.95 | 0.40 | 2.28 | 0.909 |  |  |
| Clear cell ovarian cancer | Threonine | Inverse variance weighted (fixed effects) | 3 | -0.14 | 0.26 | 0.87 | 0.52 | 1.45 | 0.586 | 0.483 |  |
| Clear cell ovarian cancer | Threonine | Maximum likelihood | 3 | -0.14 | 0.26 | 0.87 | 0.52 | 1.45 | 0.585 |  |  |
| Clear cell ovarian cancer | Threonine | Simple median | 3 | -0.05 | 0.32 | 0.95 | 0.51 | 1.78 | 0.874 |  |  |
| Clear cell ovarian cancer | Threonine | Weighted median | 3 | -0.05 | 0.30 | 0.95 | 0.53 | 1.72 | 0.867 |  |  |
| Clear cell ovarian cancer | Threonine | MR Egger | 3 | 0.63 | 1.14 | 1.87 | 0.20 | 17.49 | 0.681 |  | 0.615 |
| Clear cell ovarian cancer | trans-Hydroxyproline | Wald ratio | 1 | 0.29 | 0.50 | 1.33 | 0.51 | 3.53 | 0.560 | NA | NA |
| Clear cell ovarian cancer | Tryptophan | Inverse variance weighted (fixed effects) | 2 | 0.01 | 0.30 | 1.01 | 0.56 | 1.82 | 0.979 | 0.986 |  |
| Clear cell ovarian cancer | Tryptophan | Maximum likelihood | 2 | 0.01 | 0.30 | 1.01 | 0.56 | 1.82 | 0.979 |  |  |
| Clear cell ovarian cancer | Tyrosine | Inverse variance weighted (fixed effects) | 2 | 0.15 | 0.29 | 1.16 | 0.65 | 2.07 | 0.607 | 0.791 |  |
| Clear cell ovarian cancer | Tyrosine | Maximum likelihood | 2 | 0.15 | 0.29 | 1.16 | 0.65 | 2.07 | 0.607 |  |  |
| Clear cell ovarian cancer | Valerylcarnitine | Inverse variance weighted (multiplicative random effects) | 3 | 0.18 | 0.35 | 1.19 | 0.61 | 2.34 | 0.610 | 0.021 |  |
| Clear cell ovarian cancer | Valerylcarnitine | Maximum likelihood | 3 | 0.18 | 0.18 | 1.20 | 0.85 | 1.70 | 0.311 |  |  |
| Clear cell ovarian cancer | Valerylcarnitine | Simple median | 3 | 0.13 | 0.23 | 1.14 | 0.73 | 1.77 | 0.570 |  |  |
| Clear cell ovarian cancer | Valerylcarnitine | Weighted median | 3 | 0.07 | 0.21 | 1.08 | 0.72 | 1.61 | 0.724 |  |  |
| Clear cell ovarian cancer | Valerylcarnitine | MR Egger | 3 | -0.90 | 1.49 | 0.41 | 0.02 | 7.61 | 0.656 |  | 0.593 |
| Clear cell ovarian cancer | Valine | Inverse variance weighted (fixed effects) | 4 | 0.00 | 0.37 | 1.00 | 0.48 | 2.06 | 0.998 | 0.097 |  |
| Clear cell ovarian cancer | Valine | Maximum likelihood | 4 | 0.00 | 0.37 | 1.00 | 0.48 | 2.07 | 0.998 |  |  |
| Clear cell ovarian cancer | Valine | Simple median | 4 | 0.07 | 0.53 | 1.07 | 0.38 | 3.02 | 0.895 |  |  |
| Clear cell ovarian cancer | Valine | Weighted median | 4 | 0.24 | 0.44 | 1.27 | 0.53 | 3.02 | 0.591 |  |  |
| Clear cell ovarian cancer | Valine | MR Egger | 4 | 2.53 | 2.69 | 12.57 | 0.06 | 2434.47 | 0.446 |  | 0.437 |

| **Supplementary Table 8.** The association between exposure and Endometrioid ovarian cancer. | | | | | | | | | | | |
| --- | --- | --- | --- | --- | --- | --- | --- | --- | --- | --- | --- |
| Outcome | Exposure | Method | No. | beta | se | OR | LCI | UCI | *P*-value | *P* for heterogenity | *P* for pleiotropy |
| Endometrioid ovarian cancer | Acetylcarnitine | Inverse variance weighted (fixed effects) | 3 | -0.14 | 0.11 | 0.87 | 0.70 | 1.08 | 0.195 | 0.913 |  |
| Endometrioid ovarian cancer | Acetylcarnitine | Maximum likelihood | 3 | -0.14 | 0.11 | 0.87 | 0.70 | 1.08 | 0.195 |  |  |
| Endometrioid ovarian cancer | Acetylcarnitine | Simple median | 3 | -0.13 | 0.16 | 0.88 | 0.64 | 1.21 | 0.434 |  |  |
| Endometrioid ovarian cancer | Acetylcarnitine | Weighted median | 3 | -0.16 | 0.11 | 0.86 | 0.69 | 1.07 | 0.167 |  |  |
| Endometrioid ovarian cancer | Acetylcarnitine | MR Egger | 3 | -0.24 | 0.28 | 0.78 | 0.46 | 1.35 | 0.540 |  | 0.758 |
| Endometrioid ovarian cancer | Acetylornithine | Inverse variance weighted (fixed effects) | 2 | -0.04 | 0.04 | 0.96 | 0.89 | 1.04 | 0.343 | 0.935 |  |
| Endometrioid ovarian cancer | Acetylornithine | Maximum likelihood | 2 | -0.04 | 0.04 | 0.96 | 0.89 | 1.04 | 0.343 |  |  |
| Endometrioid ovarian cancer | Alanine | Inverse variance weighted (fixed effects) | 9 | -0.40 | 0.24 | 0.67 | 0.42 | 1.06 | 0.088 | 0.963 |  |
| Endometrioid ovarian cancer | Alanine | Maximum likelihood | 9 | -0.41 | 0.24 | 0.67 | 0.42 | 1.06 | 0.088 |  |  |
| Endometrioid ovarian cancer | Alanine | Simple median | 9 | -0.35 | 0.30 | 0.70 | 0.39 | 1.26 | 0.240 |  |  |
| Endometrioid ovarian cancer | Alanine | Weighted median | 9 | -0.31 | 0.29 | 0.74 | 0.41 | 1.31 | 0.299 |  |  |
| Endometrioid ovarian cancer | Alanine | MR Egger | 9 | -0.01 | 0.94 | 0.99 | 0.16 | 6.24 | 0.995 |  | 0.674 |
| Endometrioid ovarian cancer | alpha-Aminoadipic acid | Inverse variance weighted (fixed effects) | 2 | -0.11 | 0.26 | 0.90 | 0.54 | 1.48 | 0.666 | 0.106 |  |
| Endometrioid ovarian cancer | alpha-Aminoadipic acid | Maximum likelihood | 2 | -0.11 | 0.26 | 0.89 | 0.54 | 1.49 | 0.662 |  |  |
| Endometrioid ovarian cancer | Arginine | Inverse variance weighted (fixed effects) | 7 | -0.12 | 0.13 | 0.88 | 0.69 | 1.14 | 0.338 | 0.348 |  |
| Endometrioid ovarian cancer | Arginine | Maximum likelihood | 7 | -0.12 | 0.13 | 0.88 | 0.69 | 1.14 | 0.335 |  |  |
| Endometrioid ovarian cancer | Arginine | Simple median | 7 | 0.16 | 0.20 | 1.17 | 0.79 | 1.75 | 0.431 |  |  |
| Endometrioid ovarian cancer | Arginine | Weighted median | 7 | -0.11 | 0.16 | 0.89 | 0.65 | 1.23 | 0.496 |  |  |
| Endometrioid ovarian cancer | Arginine | MR Egger | 7 | -0.27 | 0.24 | 0.76 | 0.48 | 1.20 | 0.295 |  | 0.457 |
| Endometrioid ovarian cancer | Asparagine | Inverse variance weighted (fixed effects) | 4 | 0.12 | 0.09 | 1.13 | 0.95 | 1.35 | 0.176 | 0.729 |  |
| Endometrioid ovarian cancer | Asparagine | Maximum likelihood | 4 | 0.12 | 0.09 | 1.13 | 0.95 | 1.35 | 0.176 |  |  |
| Endometrioid ovarian cancer | Asparagine | Simple median | 4 | 0.13 | 0.18 | 1.14 | 0.80 | 1.62 | 0.485 |  |  |
| Endometrioid ovarian cancer | Asparagine | Weighted median | 4 | 0.10 | 0.09 | 1.11 | 0.92 | 1.33 | 0.281 |  |  |
| Endometrioid ovarian cancer | Asparagine | MR Egger | 4 | 0.06 | 0.13 | 1.06 | 0.83 | 1.36 | 0.677 |  | 0.552 |
| Endometrioid ovarian cancer | Aspartate | Inverse variance weighted (fixed effects) | 2 | 0.32 | 0.17 | 1.37 | 0.98 | 1.92 | 0.066 | 0.429 |  |
| Endometrioid ovarian cancer | Aspartate | Maximum likelihood | 2 | 0.32 | 0.17 | 1.37 | 0.98 | 1.92 | 0.067 |  |  |
| Endometrioid ovarian cancer | Butyrylcarnitine | Inverse variance weighted (fixed effects) | 2 | -0.10 | 0.16 | 0.91 | 0.67 | 1.23 | 0.523 | 0.117 |  |
| Endometrioid ovarian cancer | Butyrylcarnitine | Maximum likelihood | 2 | -0.10 | 0.16 | 0.91 | 0.67 | 1.23 | 0.522 |  |  |
| Endometrioid ovarian cancer | Carnitine | Inverse variance weighted (fixed effects) | 5 | -0.10 | 0.07 | 0.91 | 0.79 | 1.04 | 0.168 | 0.825 |  |
| Endometrioid ovarian cancer | Carnitine | Maximum likelihood | 5 | -0.10 | 0.07 | 0.91 | 0.79 | 1.04 | 0.168 |  |  |
| Endometrioid ovarian cancer | Carnitine | Simple median | 5 | 0.06 | 0.16 | 1.07 | 0.78 | 1.46 | 0.683 |  |  |
| Endometrioid ovarian cancer | Carnitine | Weighted median | 5 | -0.11 | 0.08 | 0.90 | 0.78 | 1.04 | 0.162 |  |  |
| Endometrioid ovarian cancer | Carnitine | MR Egger | 5 | -0.13 | 0.13 | 0.88 | 0.68 | 1.14 | 0.403 |  | 0.811 |
| Endometrioid ovarian cancer | Citrulline | Inverse variance weighted (fixed effects) | 4 | 0.50 | 0.18 | 1.65 | 1.17 | 2.34 | 0.005 | 0.259 |  |
| Endometrioid ovarian cancer | Citrulline | Maximum likelihood | 4 | 0.51 | 0.18 | 1.66 | 1.17 | 2.38 | 0.005 |  |  |
| Endometrioid ovarian cancer | Citrulline | Simple median | 4 | 0.49 | 0.21 | 1.64 | 1.08 | 2.49 | 0.021 |  |  |
| Endometrioid ovarian cancer | Citrulline | Weighted median | 4 | 0.59 | 0.22 | 1.80 | 1.16 | 2.80 | 0.008 |  |  |
| Endometrioid ovarian cancer | Citrulline | MR Egger | 4 | -0.83 | 1.36 | 0.44 | 0.03 | 6.32 | 0.606 |  | 0.428 |
| Endometrioid ovarian cancer | Creatinine | Inverse variance weighted (fixed effects) | 12 | -0.17 | 0.23 | 0.84 | 0.54 | 1.32 | 0.458 | 0.880 |  |
| Endometrioid ovarian cancer | Creatinine | Maximum likelihood | 12 | -0.17 | 0.23 | 0.85 | 0.54 | 1.32 | 0.464 |  |  |
| Endometrioid ovarian cancer | Creatinine | Simple median | 12 | -0.18 | 0.28 | 0.83 | 0.48 | 1.46 | 0.524 |  |  |
| Endometrioid ovarian cancer | Creatinine | Weighted median | 12 | -0.21 | 0.29 | 0.81 | 0.46 | 1.43 | 0.472 |  |  |
| Endometrioid ovarian cancer | Creatinine | MR Egger | 12 | -0.32 | 1.07 | 0.72 | 0.09 | 5.91 | 0.770 |  | 0.887 |
| Endometrioid ovarian cancer | Decanoylcarnitine | Inverse variance weighted (fixed effects) | 2 | -0.06 | 0.11 | 0.94 | 0.75 | 1.17 | 0.580 | 0.392 |  |
| Endometrioid ovarian cancer | Decanoylcarnitine | Maximum likelihood | 2 | -0.06 | 0.11 | 0.94 | 0.75 | 1.17 | 0.580 |  |  |
| Endometrioid ovarian cancer | Decenoylcarnitine | Inverse variance weighted (fixed effects) | 3 | -0.06 | 0.13 | 0.95 | 0.73 | 1.23 | 0.675 | 0.649 |  |
| Endometrioid ovarian cancer | Decenoylcarnitine | Maximum likelihood | 3 | -0.06 | 0.13 | 0.95 | 0.73 | 1.23 | 0.675 |  |  |
| Endometrioid ovarian cancer | Decenoylcarnitine | Simple median | 3 | -0.01 | 0.19 | 0.99 | 0.69 | 1.43 | 0.971 |  |  |
| Endometrioid ovarian cancer | Decenoylcarnitine | Weighted median | 3 | 0.01 | 0.15 | 1.01 | 0.75 | 1.35 | 0.960 |  |  |
| Endometrioid ovarian cancer | Decenoylcarnitine | MR Egger | 3 | 0.10 | 0.49 | 1.11 | 0.42 | 2.90 | 0.868 |  | 0.794 |
| Endometrioid ovarian cancer | Dodecanoylcarnitine | Wald ratio | 1 | -0.16 | 0.54 | 0.85 | 0.30 | 2.42 | 0.759 | NA | NA |
| Endometrioid ovarian cancer | Glutamine | Inverse variance weighted (fixed effects) | 5 | -0.05 | 0.25 | 0.95 | 0.58 | 1.56 | 0.845 | 0.176 |  |
| Endometrioid ovarian cancer | Glutamine | Maximum likelihood | 5 | -0.05 | 0.25 | 0.95 | 0.58 | 1.56 | 0.844 |  |  |
| Endometrioid ovarian cancer | Glutamine | Simple median | 5 | 0.11 | 0.35 | 1.12 | 0.56 | 2.23 | 0.758 |  |  |
| Endometrioid ovarian cancer | Glutamine | Weighted median | 5 | 0.12 | 0.30 | 1.12 | 0.62 | 2.04 | 0.701 |  |  |
| Endometrioid ovarian cancer | Glutamine | MR Egger | 5 | 1.68 | 1.20 | 5.34 | 0.51 | 55.85 | 0.256 |  | 0.235 |
| Endometrioid ovarian cancer | Glycine | Inverse variance weighted (fixed effects) | 12 | -0.08 | 0.06 | 0.92 | 0.81 | 1.05 | 0.213 | 0.067 |  |
| Endometrioid ovarian cancer | Glycine | Maximum likelihood | 12 | -0.08 | 0.06 | 0.92 | 0.81 | 1.05 | 0.207 |  |  |
| Endometrioid ovarian cancer | Glycine | Simple median | 12 | -0.40 | 0.20 | 0.67 | 0.45 | 1.00 | 0.049 |  |  |
| Endometrioid ovarian cancer | Glycine | Weighted median | 12 | -0.02 | 0.07 | 0.98 | 0.86 | 1.13 | 0.799 |  |  |
| Endometrioid ovarian cancer | Glycine | MR Egger | 12 | 0.07 | 0.08 | 1.07 | 0.92 | 1.26 | 0.402 |  | 0.012 |
| Endometrioid ovarian cancer | Hexadecanoylcarnitine | Inverse variance weighted (fixed effects) | 2 | -0.21 | 0.21 | 0.81 | 0.54 | 1.22 | 0.308 | 0.371 |  |
| Endometrioid ovarian cancer | Hexadecanoylcarnitine | Maximum likelihood | 2 | -0.21 | 0.21 | 0.81 | 0.54 | 1.22 | 0.308 |  |  |
| Endometrioid ovarian cancer | Hexadecenoylcarnitine | Wald ratio | 1 | -0.44 | 0.32 | 0.65 | 0.34 | 1.21 | 0.174 | NA | NA |
| Endometrioid ovarian cancer | Hexanoylcarnitine | Inverse variance weighted (fixed effects) | 3 | 0.01 | 0.09 | 1.01 | 0.84 | 1.22 | 0.896 | 0.055 |  |
| Endometrioid ovarian cancer | Hexanoylcarnitine | Maximum likelihood | 3 | 0.01 | 0.10 | 1.01 | 0.84 | 1.22 | 0.896 |  |  |
| Endometrioid ovarian cancer | Hexanoylcarnitine | Simple median | 3 | -0.28 | 0.18 | 0.75 | 0.53 | 1.07 | 0.118 |  |  |
| Endometrioid ovarian cancer | Hexanoylcarnitine | Weighted median | 3 | -0.02 | 0.10 | 0.98 | 0.81 | 1.19 | 0.855 |  |  |
| Endometrioid ovarian cancer | Hexanoylcarnitine | MR Egger | 3 | 0.23 | 0.13 | 1.26 | 0.97 | 1.63 | 0.330 |  | 0.252 |
| Endometrioid ovarian cancer | Hexose | Wald ratio | 1 | -0.07 | 0.32 | 0.94 | 0.50 | 1.76 | 0.838 | NA | NA |
| Endometrioid ovarian cancer | Histidine | Inverse variance weighted (fixed effects) | 7 | -0.17 | 0.21 | 0.84 | 0.56 | 1.27 | 0.411 | 0.056 |  |
| Endometrioid ovarian cancer | Histidine | Maximum likelihood | 7 | -0.18 | 0.21 | 0.84 | 0.56 | 1.27 | 0.407 |  |  |
| Endometrioid ovarian cancer | Histidine | Simple median | 7 | -0.01 | 0.33 | 0.99 | 0.51 | 1.89 | 0.965 |  |  |
| Endometrioid ovarian cancer | Histidine | Weighted median | 7 | 0.07 | 0.30 | 1.07 | 0.60 | 1.92 | 0.814 |  |  |
| Endometrioid ovarian cancer | Histidine | MR Egger | 7 | -0.97 | 1.77 | 0.38 | 0.01 | 12.04 | 0.605 |  | 0.663 |
| Endometrioid ovarian cancer | Isoleucine | Inverse variance weighted (fixed effects) | 3 | 0.07 | 0.43 | 1.07 | 0.47 | 2.47 | 0.869 | 0.249 |  |
| Endometrioid ovarian cancer | Isoleucine | Maximum likelihood | 3 | 0.07 | 0.43 | 1.07 | 0.46 | 2.49 | 0.868 |  |  |
| Endometrioid ovarian cancer | Isoleucine | Simple median | 3 | -0.25 | 0.60 | 0.78 | 0.24 | 2.55 | 0.680 |  |  |
| Endometrioid ovarian cancer | Isoleucine | Weighted median | 3 | 0.00 | 0.51 | 1.00 | 0.37 | 2.74 | 0.993 |  |  |
| Endometrioid ovarian cancer | Isoleucine | MR Egger | 3 | 0.45 | 3.17 | 1.57 | 0.00 | 787.28 | 0.910 |  | 0.922 |
| Endometrioid ovarian cancer | Kynurenine | Inverse variance weighted (fixed effects) | 3 | 0.04 | 0.14 | 1.04 | 0.79 | 1.35 | 0.796 | 0.339 |  |
| Endometrioid ovarian cancer | Kynurenine | Maximum likelihood | 3 | 0.04 | 0.14 | 1.04 | 0.79 | 1.35 | 0.796 |  |  |
| Endometrioid ovarian cancer | Kynurenine | Simple median | 3 | -0.04 | 0.18 | 0.96 | 0.67 | 1.37 | 0.821 |  |  |
| Endometrioid ovarian cancer | Kynurenine | Weighted median | 3 | -0.04 | 0.15 | 0.96 | 0.72 | 1.29 | 0.789 |  |  |
| Endometrioid ovarian cancer | Kynurenine | MR Egger | 3 | -0.38 | 0.40 | 0.68 | 0.31 | 1.48 | 0.511 |  | 0.463 |
| Endometrioid ovarian cancer | Leucine | Inverse variance weighted (fixed effects) | 4 | 0.26 | 0.38 | 1.29 | 0.61 | 2.72 | 0.499 | 0.718 |  |
| Endometrioid ovarian cancer | Leucine | Maximum likelihood | 4 | 0.26 | 0.38 | 1.29 | 0.61 | 2.73 | 0.498 |  |  |
| Endometrioid ovarian cancer | Leucine | Simple median | 4 | 0.12 | 0.46 | 1.13 | 0.46 | 2.78 | 0.796 |  |  |
| Endometrioid ovarian cancer | Leucine | Weighted median | 4 | 0.44 | 0.45 | 1.55 | 0.64 | 3.72 | 0.329 |  |  |
| Endometrioid ovarian cancer | Leucine | MR Egger | 4 | 0.15 | 1.23 | 1.16 | 0.11 | 12.83 | 0.914 |  | 0.935 |
| Endometrioid ovarian cancer | Lysine | Inverse variance weighted (fixed effects) | 6 | -0.13 | 0.12 | 0.88 | 0.70 | 1.10 | 0.265 | 0.379 |  |
| Endometrioid ovarian cancer | Lysine | Maximum likelihood | 6 | -0.13 | 0.12 | 0.88 | 0.70 | 1.10 | 0.264 |  |  |
| Endometrioid ovarian cancer | Lysine | Simple median | 6 | -0.12 | 0.17 | 0.88 | 0.64 | 1.23 | 0.469 |  |  |
| Endometrioid ovarian cancer | Lysine | Weighted median | 6 | -0.11 | 0.14 | 0.90 | 0.68 | 1.18 | 0.433 |  |  |
| Endometrioid ovarian cancer | Lysine | MR Egger | 6 | -0.22 | 0.31 | 0.80 | 0.43 | 1.47 | 0.509 |  | 0.751 |
| Endometrioid ovarian cancer | Methionine | Wald ratio | 1 | 0.38 | 0.49 | 1.46 | 0.56 | 3.83 | 0.437 | NA | NA |
| Endometrioid ovarian cancer | Methioninesulfoxide | Wald ratio | 1 | -0.38 | 0.41 | 0.68 | 0.31 | 1.51 | 0.349 | NA | NA |
| Endometrioid ovarian cancer | Methylglutarylcarnitine | Inverse variance weighted (fixed effects) | 4 | 0.02 | 0.12 | 1.02 | 0.81 | 1.30 | 0.839 | 0.982 |  |
| Endometrioid ovarian cancer | Methylglutarylcarnitine | Maximum likelihood | 4 | 0.02 | 0.12 | 1.02 | 0.81 | 1.30 | 0.839 |  |  |
| Endometrioid ovarian cancer | Methylglutarylcarnitine | Simple median | 4 | 0.03 | 0.15 | 1.04 | 0.76 | 1.40 | 0.824 |  |  |
| Endometrioid ovarian cancer | Methylglutarylcarnitine | Weighted median | 4 | 0.03 | 0.13 | 1.03 | 0.80 | 1.33 | 0.793 |  |  |
| Endometrioid ovarian cancer | Methylglutarylcarnitine | MR Egger | 4 | 0.07 | 0.35 | 1.08 | 0.54 | 2.13 | 0.852 |  | 0.894 |
| Endometrioid ovarian cancer | Nonaylcarnitine | Inverse variance weighted (fixed effects) | 2 | 0.17 | 0.11 | 1.18 | 0.95 | 1.46 | 0.130 | 0.249 |  |
| Endometrioid ovarian cancer | Nonaylcarnitine | Maximum likelihood | 2 | 0.17 | 0.11 | 1.18 | 0.95 | 1.47 | 0.131 |  |  |
| Endometrioid ovarian cancer | Octadecandienylcarnitine | Inverse variance weighted (fixed effects) | 4 | -0.16 | 0.11 | 0.86 | 0.69 | 1.06 | 0.153 | 0.445 |  |
| Endometrioid ovarian cancer | Octadecandienylcarnitine | Maximum likelihood | 4 | -0.16 | 0.11 | 0.85 | 0.69 | 1.06 | 0.153 |  |  |
| Endometrioid ovarian cancer | Octadecandienylcarnitine | Simple median | 4 | -0.13 | 0.14 | 0.88 | 0.67 | 1.15 | 0.340 |  |  |
| Endometrioid ovarian cancer | Octadecandienylcarnitine | Weighted median | 4 | -0.16 | 0.12 | 0.85 | 0.67 | 1.08 | 0.190 |  |  |
| Endometrioid ovarian cancer | Octadecandienylcarnitine | MR Egger | 4 | -0.10 | 0.30 | 0.90 | 0.50 | 1.62 | 0.764 |  | 0.862 |
| Endometrioid ovarian cancer | Octadecanoylcarnitine | Inverse variance weighted (fixed effects) | 5 | -0.08 | 0.17 | 0.92 | 0.66 | 1.28 | 0.633 | 0.648 |  |
| Endometrioid ovarian cancer | Octadecanoylcarnitine | Maximum likelihood | 5 | -0.08 | 0.17 | 0.92 | 0.66 | 1.28 | 0.632 |  |  |
| Endometrioid ovarian cancer | Octadecanoylcarnitine | Simple median | 5 | -0.08 | 0.24 | 0.93 | 0.58 | 1.48 | 0.750 |  |  |
| Endometrioid ovarian cancer | Octadecanoylcarnitine | Weighted median | 5 | -0.06 | 0.20 | 0.94 | 0.63 | 1.40 | 0.768 |  |  |
| Endometrioid ovarian cancer | Octadecanoylcarnitine | MR Egger | 5 | 0.40 | 0.98 | 1.49 | 0.22 | 10.12 | 0.712 |  | 0.654 |
| Endometrioid ovarian cancer | Octadecenoylcarnitine | Inverse variance weighted (fixed effects) | 2 | -0.20 | 0.15 | 0.82 | 0.61 | 1.10 | 0.191 | 0.625 |  |
| Endometrioid ovarian cancer | Octadecenoylcarnitine | Maximum likelihood | 2 | -0.20 | 0.15 | 0.82 | 0.61 | 1.11 | 0.193 |  |  |
| Endometrioid ovarian cancer | Octanoylcarnitine | Inverse variance weighted (fixed effects) | 5 | -0.07 | 0.09 | 0.93 | 0.78 | 1.11 | 0.414 | 0.581 |  |
| Endometrioid ovarian cancer | Octanoylcarnitine | Maximum likelihood | 5 | -0.07 | 0.09 | 0.93 | 0.78 | 1.11 | 0.414 |  |  |
| Endometrioid ovarian cancer | Octanoylcarnitine | Simple median | 5 | -0.17 | 0.16 | 0.84 | 0.62 | 1.15 | 0.278 |  |  |
| Endometrioid ovarian cancer | Octanoylcarnitine | Weighted median | 5 | -0.05 | 0.10 | 0.95 | 0.78 | 1.15 | 0.581 |  |  |
| Endometrioid ovarian cancer | Octanoylcarnitine | MR Egger | 5 | 0.18 | 0.18 | 1.20 | 0.83 | 1.72 | 0.405 |  | 0.221 |
| Endometrioid ovarian cancer | Ornithine | Inverse variance weighted (multiplicative random effects) | 6 | 0.05 | 0.31 | 1.05 | 0.57 | 1.93 | 0.870 | 0.010 |  |
| Endometrioid ovarian cancer | Ornithine | Maximum likelihood | 6 | 0.05 | 0.18 | 1.05 | 0.74 | 1.51 | 0.771 |  |  |
| Endometrioid ovarian cancer | Ornithine | Simple median | 6 | -0.33 | 0.26 | 0.72 | 0.44 | 1.19 | 0.202 |  |  |
| Endometrioid ovarian cancer | Ornithine | Weighted median | 6 | -0.26 | 0.26 | 0.77 | 0.46 | 1.30 | 0.329 |  |  |
| Endometrioid ovarian cancer | Ornithine | MR Egger | 6 | 1.12 | 0.62 | 3.08 | 0.91 | 10.36 | 0.144 |  | 0.131 |
| Endometrioid ovarian cancer | Phenylalanine | Inverse variance weighted (fixed effects) | 6 | 0.13 | 0.23 | 1.14 | 0.72 | 1.80 | 0.570 | 0.131 |  |
| Endometrioid ovarian cancer | Phenylalanine | Maximum likelihood | 6 | 0.13 | 0.23 | 1.14 | 0.72 | 1.81 | 0.566 |  |  |
| Endometrioid ovarian cancer | Phenylalanine | Simple median | 6 | 0.37 | 0.34 | 1.45 | 0.74 | 2.83 | 0.278 |  |  |
| Endometrioid ovarian cancer | Phenylalanine | Weighted median | 6 | 0.20 | 0.33 | 1.22 | 0.64 | 2.32 | 0.549 |  |  |
| Endometrioid ovarian cancer | Phenylalanine | MR Egger | 6 | -1.03 | 1.21 | 0.36 | 0.03 | 3.85 | 0.443 |  | 0.378 |
| Endometrioid ovarian cancer | Proline | Inverse variance weighted (fixed effects) | 2 | 0.13 | 0.11 | 1.13 | 0.92 | 1.40 | 0.239 | 0.431 |  |
| Endometrioid ovarian cancer | Proline | Maximum likelihood | 2 | 0.13 | 0.11 | 1.13 | 0.92 | 1.40 | 0.239 |  |  |
| Endometrioid ovarian cancer | Propionylcarnitine | Inverse variance weighted (fixed effects) | 4 | -0.15 | 0.10 | 0.86 | 0.70 | 1.06 | 0.158 | 0.902 |  |
| Endometrioid ovarian cancer | Propionylcarnitine | Maximum likelihood | 4 | -0.15 | 0.10 | 0.86 | 0.70 | 1.06 | 0.158 |  |  |
| Endometrioid ovarian cancer | Propionylcarnitine | Simple median | 4 | -0.12 | 0.15 | 0.89 | 0.66 | 1.19 | 0.418 |  |  |
| Endometrioid ovarian cancer | Propionylcarnitine | Weighted median | 4 | -0.15 | 0.11 | 0.86 | 0.69 | 1.06 | 0.162 |  |  |
| Endometrioid ovarian cancer | Propionylcarnitine | MR Egger | 4 | -0.15 | 0.25 | 0.86 | 0.53 | 1.39 | 0.603 |  | 0.992 |
| Endometrioid ovarian cancer | Sarcosine | Inverse variance weighted (fixed effects) | 2 | 0.13 | 0.26 | 1.14 | 0.69 | 1.88 | 0.615 | 0.391 |  |
| Endometrioid ovarian cancer | Sarcosine | Maximum likelihood | 2 | 0.13 | 0.26 | 1.14 | 0.69 | 1.88 | 0.615 |  |  |
| Endometrioid ovarian cancer | Serine | Inverse variance weighted (fixed effects) | 4 | -0.08 | 0.10 | 0.92 | 0.75 | 1.12 | 0.404 | 0.717 |  |
| Endometrioid ovarian cancer | Serine | Maximum likelihood | 4 | -0.08 | 0.10 | 0.92 | 0.75 | 1.12 | 0.403 |  |  |
| Endometrioid ovarian cancer | Serine | Simple median | 4 | -0.03 | 0.12 | 0.97 | 0.76 | 1.23 | 0.789 |  |  |
| Endometrioid ovarian cancer | Serine | Weighted median | 4 | -0.05 | 0.12 | 0.95 | 0.75 | 1.19 | 0.644 |  |  |
| Endometrioid ovarian cancer | Serine | MR Egger | 4 | -0.24 | 0.39 | 0.78 | 0.36 | 1.69 | 0.597 |  | 0.714 |
| Endometrioid ovarian cancer | Spermidine | Wald ratio | 1 | -0.30 | 0.17 | 0.74 | 0.53 | 1.04 | 0.085 | NA | NA |
| Endometrioid ovarian cancer | Symmetric dimethylarginine | Wald ratio | 1 | 0.38 | 0.25 | 1.46 | 0.91 | 2.37 | 0.120 | NA | NA |
| Endometrioid ovarian cancer | Taurine | Wald ratio | 1 | 0.19 | 0.21 | 1.21 | 0.80 | 1.83 | 0.367 | NA | NA |
| Endometrioid ovarian cancer | Tetradecanoylcarnitine | Inverse variance weighted (fixed effects) | 3 | -0.33 | 0.19 | 0.72 | 0.50 | 1.04 | 0.083 | 0.653 |  |
| Endometrioid ovarian cancer | Tetradecanoylcarnitine | Maximum likelihood | 3 | -0.33 | 0.19 | 0.72 | 0.50 | 1.05 | 0.084 |  |  |
| Endometrioid ovarian cancer | Tetradecanoylcarnitine | Simple median | 3 | -0.42 | 0.25 | 0.66 | 0.41 | 1.07 | 0.091 |  |  |
| Endometrioid ovarian cancer | Tetradecanoylcarnitine | Weighted median | 3 | -0.32 | 0.21 | 0.72 | 0.48 | 1.09 | 0.124 |  |  |
| Endometrioid ovarian cancer | Tetradecanoylcarnitine | MR Egger | 3 | 0.50 | 1.22 | 1.66 | 0.15 | 18.22 | 0.751 |  | 0.617 |
| Endometrioid ovarian cancer | Tetradecenoylcarnitine | Inverse variance weighted (fixed effects) | 2 | -0.29 | 0.32 | 0.75 | 0.40 | 1.39 | 0.359 | 0.604 |  |
| Endometrioid ovarian cancer | Tetradecenoylcarnitine | Maximum likelihood | 2 | -0.29 | 0.32 | 0.75 | 0.40 | 1.40 | 0.360 |  |  |
| Endometrioid ovarian cancer | Threonine | Inverse variance weighted (fixed effects) | 3 | -0.10 | 0.19 | 0.90 | 0.63 | 1.30 | 0.587 | 0.291 |  |
| Endometrioid ovarian cancer | Threonine | Maximum likelihood | 3 | -0.10 | 0.19 | 0.90 | 0.62 | 1.30 | 0.586 |  |  |
| Endometrioid ovarian cancer | Threonine | Simple median | 3 | -0.01 | 0.27 | 0.99 | 0.58 | 1.69 | 0.969 |  |  |
| Endometrioid ovarian cancer | Threonine | Weighted median | 3 | 0.03 | 0.21 | 1.03 | 0.69 | 1.54 | 0.898 |  |  |
| Endometrioid ovarian cancer | Threonine | MR Egger | 3 | 0.53 | 1.12 | 1.69 | 0.19 | 15.06 | 0.719 |  | 0.666 |
| Endometrioid ovarian cancer | trans-Hydroxyproline | Wald ratio | 1 | -0.23 | 0.37 | 0.79 | 0.39 | 1.63 | 0.529 | NA | NA |
| Endometrioid ovarian cancer | Tryptophan | Inverse variance weighted (fixed effects) | 2 | 0.54 | 0.22 | 1.72 | 1.12 | 2.64 | 0.013 | 0.698 |  |
| Endometrioid ovarian cancer | Tryptophan | Maximum likelihood | 2 | 0.54 | 0.22 | 1.72 | 1.11 | 2.66 | 0.015 |  |  |
| Endometrioid ovarian cancer | Tyrosine | Inverse variance weighted (fixed effects) | 2 | -0.23 | 0.22 | 0.80 | 0.52 | 1.22 | 0.295 | 0.196 |  |
| Endometrioid ovarian cancer | Tyrosine | Maximum likelihood | 2 | -0.23 | 0.22 | 0.80 | 0.52 | 1.22 | 0.295 |  |  |
| Endometrioid ovarian cancer | Valerylcarnitine | Inverse variance weighted (fixed effects) | 3 | -0.12 | 0.13 | 0.89 | 0.70 | 1.14 | 0.353 | 0.511 |  |
| Endometrioid ovarian cancer | Valerylcarnitine | Maximum likelihood | 3 | -0.12 | 0.13 | 0.89 | 0.69 | 1.14 | 0.352 |  |  |
| Endometrioid ovarian cancer | Valerylcarnitine | Simple median | 3 | -0.07 | 0.16 | 0.94 | 0.68 | 1.29 | 0.684 |  |  |
| Endometrioid ovarian cancer | Valerylcarnitine | Weighted median | 3 | -0.09 | 0.14 | 0.92 | 0.70 | 1.20 | 0.531 |  |  |
| Endometrioid ovarian cancer | Valerylcarnitine | MR Egger | 3 | -0.18 | 0.55 | 0.84 | 0.29 | 2.47 | 0.803 |  | 0.930 |
| Endometrioid ovarian cancer | Valine | Inverse variance weighted (fixed effects) | 4 | 0.07 | 0.26 | 1.07 | 0.64 | 1.80 | 0.793 | 0.447 |  |
| Endometrioid ovarian cancer | Valine | Maximum likelihood | 4 | 0.07 | 0.26 | 1.07 | 0.64 | 1.80 | 0.792 |  |  |
| Endometrioid ovarian cancer | Valine | Simple median | 4 | -0.17 | 0.37 | 0.84 | 0.41 | 1.72 | 0.636 |  |  |
| Endometrioid ovarian cancer | Valine | Weighted median | 4 | 0.08 | 0.30 | 1.08 | 0.60 | 1.95 | 0.791 |  |  |
| Endometrioid ovarian cancer | Valine | MR Egger | 4 | -0.28 | 1.48 | 0.75 | 0.04 | 13.85 | 0.867 |  | 0.832 |
